# Utility of novel 2-furanones in synthesis of other heterocyclic compounds having anti-inflammatory activity with dual COX2/LOX inhibition

**DOI:** 10.1080/14756366.2021.1908277

**Published:** 2021-05-06

**Authors:** Rania H. Abd El-Hameed, Shahenda Mahgoub, Hend M. El-Shanbaky, Mosaad S. Mohamed, Sahar A. Ali

**Affiliations:** a Pharmaceutical Organic Chemistry Department, Faculty of Pharmacy, Helwan University, Cairo, Egypt; b Department of Biochemistry and Molecular Biology, Faculty of Pharmacy, Helwan University, Cairo, Egypt

**Keywords:** Pyridazinone, selective COX-2 inhibitor, 15-LOX inhibitors, TNF-α inhibitor, anti-inflammatory

## Abstract

Inflammation is associated with the development of several diseases comprising cancer and cardiovascular disease. Agents that suppress cyclooxygenase (COX) and lipoxygenase (LOX) enzymes, besides chemokines have been suggested to minimise inflammation. Here, a variety of novel heterocyclic and non-heterocyclic compounds were prepared from novel three furanone derivatives. The structures of all synthesised compounds were confirmed by elemental and spectral analysis including mass, IR, and ^1^H-NMR spectroscopy. Anti-inflammatory activities of these synthesised compounds were examined *in vitro* against COX enzymes, 15-LOX, and tumour necrosis factor-α (TNF-α), using inhibition screening assays. The majority of these derivatives showed significant to high activities, with three pyridazinone derivatives (**5b, 8b,** and **8c**) being the most promising anti-inflammatory agents with dual COX-2/15-LOX inhibition activities along with high TNF-α inhibition activity.

## 1. Introduction

Inflammation is a protective physiological defence mechanism provided by the body immune system in response to toxins, infectious pathogens, and local injury^1^. It occurs as a result of biosynthesis of pro-inflammatory mediators (leukotrienes [LTs] and prostaglandins [PGs]) from arachidonic acid (AA) by the action of the enzymes lipoxygenase (LOX) and cyclooxygenase (COX), respectively. Although inflammation is a normal defence mechanism, persistent/untreated inflammation leads to complicated events with release of many mediators that can turn the condition to be harmful and may lead to the development of certain diseases, as asthma, rheumatoid arthritis, atherosclerosis, diabetes, and cancer^2^. Therefore, anti-inflammatory agents may be helpful in the management of inflammatory disorders^3^. Anti-inflammatory effects of various compounds may result mainly from their ability to inhibit some of the key enzymes involved in inflammation and/or cell signalling pathways such as COX and LOX^4^.

Thus, inhibition of these enzymes may be valuable treatment for inflammatory conditions. In the human system, COX occurs in two isoforms: COX-1 and COX-2^5^. Both isoforms catalyse a COX reaction in which they act on AA as substrate. COX isoforms are haem containing enzymes that demonstrate distinctive expression profiles and roles in numerous physiological processes.^6^ COX-1 is constitutive isoform and is found in the gastrointestinal (GI)-tract, renal collecting tubules, and platelets and is believed to be responsible for the maintenance of physiological homeostasis such as renal function and GI integrity through production of gastroprotective PGs. On the other hand, the inducible isoform; COX-2 is released during tissue injury and induced by many kinds of inflammatory mediators; playing an important role in the proinflammatory PGs biosynthesis^7^.

Inhibition of both isoforms of COX by classical nonsteroidal anti-inflammatory drugs (NSAIDs) leads to inhibition of gastroprotective PGs produced *via* the COX-1 pathway; which occurs along with suppression of the pathological COX effects; resulting in the GI toxicities accompanying the use of numerous NSAIDs as GI irritation, bleeding, and ulceration^8^. Several studies revealed that COX-2 is highly expressed in a wide range of cancer tissues, such as colon, breast, and prostate, suggesting that it may control several cellular processes. Thus, selective COX-2 inhibitors have been extensively investigated for the treatment and prevention of a variety of cancers^9^.

Also, COX-2 is found in wide range of tissues e.g. brain, spinal cord, and kidneys, as well as many cells like vascular endothelium, suggesting that this isoenzyme may play a more complex physiological role than was expected^10^^,^^11^.

However, potent selective COX-2 inhibitors, which were used instead of NSAIDs; also showed disadvantages as incidence of vascular-diseases^12^. AA; which is the substrate of COX enzymes, is also converted by LOX enzyme to several lipid mediators recognised as eicosanoids^13^. LOXs are an exceptional group of non-haem iron-containing enzymes that catalyse the peroxidation of polyunsaturated fatty acids *viz*. AA and linoleic acid to their hydroperoxides^14^. 15-LOXs are implicated in a variety of human diseases, like the oxidative alteration of low-density lipoproteins and thus, the progression of atherosclerosis^15^. In addition to many neurodegenerative diseases as Alzheimer’s disease^16^, 15-LOX-1 inhibition has been reported to be a focal point to decrease the biosynthesis of eoxines, which are known to be pro-inflammatory mediators^17^ and promotors for cancer disease^18^. Thus, some literature work has been targeting 15-LOX-1.

Yet, the use of LOX-inhibitors might represent an insufficient single therapeutic model in inflammatory diseases other than asthma^19^^,^^20^. It was discovered that dual inhibition of the COX and LOX pathways could produce a wider spectrum of anti-inflammatory effects and can limit the vascular-changes seen during inflammation and leukocyte-induced GI damage^21^.

**Figure 1.**
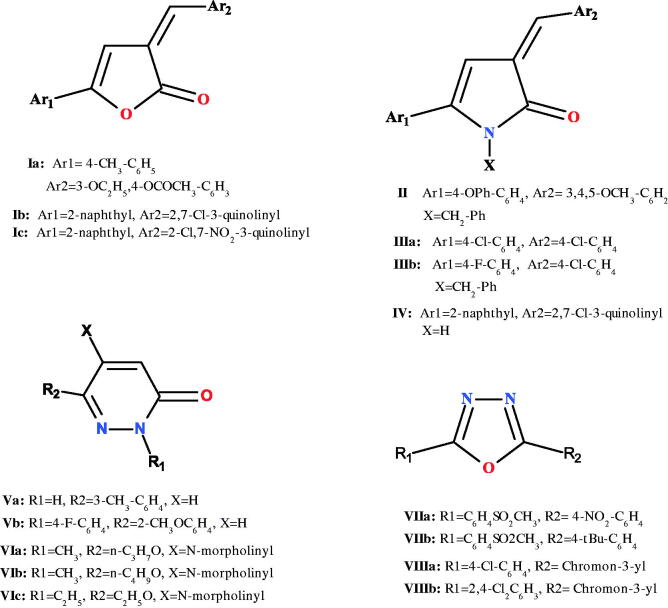
2-furanones, 2-pyrrolones, pyridazinones, and oxadiazoles as potent anti-inflammatory agents.

2-Furanones; well-known heterocyclic derivatives; had attracted a great attention during the last decade due to facile ring opening and conversion to other heterocycles; pyrrolones, pyridazinones, pyrazoles, and oxadizoles. These heterocycles acquired an obvious medicinal interest as antimicrobial^22–24^, antiviral^25–27^, antimycobacterial^28^^,^^29^, and anti-cancer agents^30–32^. Literature is enriched with different 2-furanones subjected to ring opening to form 2-pyrrolone, pyridazinone, and oxadiazole derivatives, all have high activity as anti-inflammatory^24^^,^^33–41^. As shown in Figure 1, 2-furanone derivatives **Ia–c** were reported to exhibit comparable anti-inflammatory activity to that of diclofenac^33^^,^^35^^,^^36^, while 2-pyrrolone derivatives **II, IIIa–b,** and **IV** were reported to have comparable anti-inflammatory activity to that of indomethacin, ibuprofen, and diclofenac, respectively^24^^,^^34–36^ .

Many pyridazinone derivatives were also reported as **Va,b**; which have superior anti-inflammatory activities over celecoxib and indomethacin, while derivatives **VIa–c** were more potent than aminopyrine, mebirizole, phenylbutazone, and mefenamic acid^37^^,^^38^. Some of oxadiazole derivatives **VIIa,b** showed superior activity over celecoxib besides compounds **VIIIa,b**; which were reported to be comparable with ibuprofen as dual COX/LOX inhibition activity^42^^,^^43^.

The anti-inflammatory activity shown by the above derivatives has drawn our interest to continue our research^44–46^ for development of new anti-inflammatory agents. The synthesis and biological evaluation of new 2-furanone derivatives are reported herein. Also 2-furanone derivatives were used for synthesis of other heterocyclic and non-heterocyclic derivatives, which were tested as for their potential as anti-inflammatory agents against COX enzymes, LOX, and tumour necrosis factor-α (TNF-α).

## 2. Materials and methods

### 2.1. Synthesis of lead compounds

All commercial chemicals used as starting materials and reagents in this study were purchased from Merck (Darmstadt, Germany) and were of reagent grade. All melting points were uncorrected and measured using Electro-thermal IA 9100 apparatus (Shimadzu, Japan); IR spectra were recorded as potassium bromide pellets on a Perkin-Elmer 1650 spectrophotometer (Waltham MA, Faculty of Science, Cairo University, Cairo, Egypt. ^1^H-NMR spectra were determined on a Varian Mercury (300 MHz) spectrometer (Varian, Crawley, UK) and chemical shifts were expressed as ppm against TMS as internal reference (The Main Chemical warfare Laboratories, Almaza, Cairo, Egypt). Mass spectra were recorded on 70 eV (EI Ms-QP 1000 EX, Shimadzu, Japan), Faculty of Science, Cairo University, Cairo, Egypt. Microanalyses were operated using Vario, Elmentar apparatus (Shimadzu, Japan), Organic Microanalysis Unit, Faculty of Science, Cairo University, Cairo, Egypt. Column Chromatography was performed on (Merck) Silica gel 60 (particle size 0.06–0.20 mm). All the listed compounds are new except compound **1** was previously reported^47^^,^^48^.

#### 2.1.1. General procedure for the synthesis of compounds 2a–c

A mixture of compound **1** (0.03 mol) and equimolar amount of aromatic aldehyde was refluxed in acetic anhydride (15 ml) with triethylamine (3–4 drops) for 4 h. After completion of reaction, the product was filtered, washed with ethanol, and recrystallised from ethanol to obtain compounds **2a–c**.

##### 2.1.1.1. 5-(3,4-Dichlorophenyl)3-(2-nitrobenzylidene)-furan-2-one (2a)

Yield: 57%; m.p.: 259–261 °C; IR (KBr) *ν* (cm^−1^): 1769 (C=O), 1515,1337 (NO_2_); MS (EI) *m/z*: 363 (M + 2, 9.35%), 361 (M, 14.24%); ^1^H-NMR (DMSO-d_6_, 300 MHz) *δ* (ppm): 7.51–8.20 (m, 9H, Ar-H + CH methyne); Anal. Calcd. for C_17_H_9_Cl_2_NO_4_ (361.05): C, 56.51; H, 2.49; N, 3.88%. Found: C, 56.69; H, 2.81; N, 3.54%.

##### 2.1.1.2. 5-(3,4-Dichlorophenyl)3-(3-nitrobenzylidene)-furan-2-one (2b)

Yield: 90%; m.p.: 230–232 °C; IR (KBr) *ν* (cm^−1^): 1781 (C=O), 1519,1345 (NO_2_); MS (EI) *m/z*: 363 (M + 2, 23.28%), 361 (M, 34.48%), 173 (benzoyl, 100%); ^1^H-NMR (DMSO-d_6_, 300 MHz) *δ* (ppm): 7.23–8.67 (m, 9H, Ar-H + CH methyne); Anal. Calcd. for C_17_H_9_Cl_2_NO_4_ (361.05): C, 56.51; H, 2.49; N, 3.88%. Found: C, 56.26; H, 2.35; N, 3.76%.

##### 2.1.1.3. 5-(3,4-Dichlorophenyl)3-(4-dimethylaminobenzylidene)-furan-2-one (2c)

Yield: 73%; m.p.: 204–206 °C; IR (KBr) *ν* (cm^−1^): 1748 (C=O); MS (EI) *m/z*: 361 (M + 2, 12.99%), 359 (M, 19.98%); ^1^H-NMR (DMSO-d_6_, 300 MHz) *δ* (ppm): 3.05 (s, 6H, N(CH_3_)_2_), 6.75–8.11 (m, 9H, Ar-H + CH methyne); Anal. Calcd. for C_19_H_15_Cl_2_NO_2_ (359.05): C, 63.51; H, 4.18; N, 3.90%. Found: C, 63.29; H, 3.96; N, 3.98%.

#### 2.1.2. General procedure for the synthesis of compounds 3a–c

A solution of the furanone derivatives **2a–c** (0.01 mol) and ammonium acetate (7.7 g, 0.1 mol) in acetic acid (10 ml) was refluxed for 3 h. The reaction mixture was left to cool at room temperature and the product obtained was filtered off, recrystallised from ethanol to give compounds **3a–c**.

##### 2.1.2.1. 5-(3,4-Dichlorophenyl)-3-(2-nitrobenzylidene)-1H-pyrrol-2-one (3a)

Yield: 80%; m.p.: >300 °C; IR (KBr) *ν* (cm^−1^): 3140 (NH), 1698 (C=O), 1514, 1350 (NO_2_); MS (EI) *m/z*: 362 (M + 2, 10.78%), 360 (M^+^, 16.29%); ^1^H-NMR (DMSO-d_6_, 300 MHz) *δ* (ppm): 6.80–8.17 (m, 9H, Ar-H + CH methyne), 10.72 (s, 1H, NH, D_2_O-exchangable); Anal. Calcd. for C_17_H_10_Cl_2_N_2_O_3_ (360.02): C, 56.67; H, 2.78; N, 7.78%. Found: C, 56.81; H, 2.55; N, 7.46%.

##### 2.1.2.2. 5-(3,4-Dichlorophenyl)-3-(3-nitrobenzylidene)-1H-pyrrol-2-one (3b)

Yield: 70%; m.p.: >300 °C; IR (KBr) *ν* (cm^−1^): 3143 (NH), 1704 (C=O), 1517, 1355 (NO_2_); MS (EI) *m/z*: 362 (M + 2, 67.3%), 360 (M^+^, 100%); ^1^H-NMR (DMSO-d_6_, 300 MHz) *δ* (ppm): 7.05–8.57 (m, 9H, Ar-H + CH methyne), 10.68 (s, 1H, NH, D_2_O-exchangable); Anal. Calcd. for C_17_H_10_Cl_2_N_2_O_3_ (360.02): C, 56.67; H, 2.78; N, 7.78%. Found: C, 56.52; H, 2.45; N, 7.43%.

##### 2.1.2.3. 5-(3,4-Dichlorophenyl)-3-(4-dimethylaminobenzylidene)-1H-pyrrol-2-one (3c)

Yield: 56%; m.p.: >300 °C; IR (KBr) *ν* (cm^−1^): 3697 (NH), 1673 (C=O); MS (EI) *m/z*: 360 (M + 2, 43.58%), 358 (M^+^, 62%); ^1^H-NMR (DMSO-d_6_, 300 MHz) *δ* (ppm): 2.98 (s, 6H, N(CH_3_)_2_), 6.46–8.37 (m, 9H, Ar-H + CH methyne), 10.37 (s, 1H, NH, D_2_O-exchangable); Anal. Calcd. for C_19_H_16_Cl_2_N_2_O (358.05): C, 63.52; H, 4.49; N, 7.80%. Found: C, 63.21; H, 4.56; N, 7.34%.

#### 2.1.3. General procedure for the synthesis of compounds 4a–c

To a solution of the furanone **2a–c** (0.01 mol) in absolute ethanol (20 ml), benzylamine (1.07 ml, 0.01 mol) was added and the reaction mixture was refluxed for 5 h. The product was filtered off, washed with ethanol, and finally recrystallised from ethanol to give the amides **4a–c**.

##### 2.1.3.1. N-benzyl-2-(2-nitrobenzylidene)-4-(3,4-dichlorophenyl)-4-oxobutanamide (4a)

Yield: 30%; m.p.: 250–252 °C; IR (KBr) *ν* (cm^−1^): 3310 (NH), 1681, 1647 (2 C=O), 1559, 1336 (NO_2_); MS (EI) *m/z*: 468 (M^+^, 0.33%), 91 (tropylium, 100%); ^1^H-NMR (DMSO-d_6_, 300 MHz) *δ* (ppm): 3.40 (s, 2H, CH_2_–C=O), 4.36 (dd, 2H, CH_2_–NH), 7.10 (s, 1H, NH, D_2_O-exchangable), 7.11–8.80 (m, 13H, Ar-H + CH-methyne); Anal. Calcd. for C_24_H_18_Cl_2_N_2_O_4_ (468.07): C, 61.42; H, 3.87; N, 5.97%. Found: C, 61.32; H, 3.51; N, 5.82%.

##### 2.1.3.2. N-benzyl-2-(3-nitrobenzylidene)-4-(3,4-dichlorophenyl)-4-oxobutanamide (4b)

Yield: 70%; m.p.: 124–126 °C; IR (KBr) *ν* (cm^−1^): 3312 (NH), 1676, 1648 (2 C=O), 1530, 1350 (NO_2_); MS (EI) *m/z*: 468 (M^+^, 0.29%); ^1^H-NMR (DMSO-d_6_, 300 MHz) *δ* (ppm): 3.61 (s, 2H, CH_2_-CO), 4.21 (dd, 2H, CH_2_–NH), 7.05–8.35 (m, 12H, Ar-H + CH-methyne + s, 1H, NH, D_2_O-exchangable); Anal. Calcd. for C_24_H_18_Cl_2_N_2_O_4_ (468.07): C, 61.42; H, 3.87; N, 5.97%. Found: C, 61.35; H, 3.74; N, 5.82%.

##### 2.1.3.3. N-benzyl-2-(4-dimethylaminobenzylidene]-4-(3,4-dichlorophenyl)-4-oxobutanamide (4c)

Yield: 40%; m.p.: 241–243 °C; IR (KBr) *ν* (cm^−1^): 3300 (NH), 1756, 1670 (2 C=O); MS (EI) *m/z*: 468 (M + 2, 1.73%), 466 (M, 3.15%), 448 (M^+^-H_2_O, 56.15%), 91 (tropylium, 100%); ^1^H-NMR (DMSO-d_6_, 300 MHz) *δ* (ppm): 2.88 (s,6H, N(CH_3_)_2_), 3.26 (s, 2H, CH_2_-CO), 4.20 (dd, 2H, CH_2_–NH), 6.93 (s, 1H, NH, D_2_O-exchangable), 6.69–7.43 (m, 13H, Ar-H + CH-methyne); Anal. Calcd. for C_26_H_24_Cl_2_N_2_O_2_ (466.09): C, 66.81; H, 5.18; N, 5.99%. Found: C, 66.75; H, 5.24; N, 5.86%.

#### 2.1.4. General procedure for the synthesis of compounds 5a–c

A solution of the furanone **2a–c** (0.01 mol) and phenyl hydrazine 3 ml in Na ethoxide (10 ml) was refluxed for 3 h. The product obtained was filtered, washed with water, and recrystallised from ethanol to give compounds **5a–c**.

##### 2.1.4.1. 6-(3,4-Dichlorophenyl)-4-(2-nitrobenzylidene)-1-phenyl-1,4-dihydropyridazin-3(2H)-one (5a)

Yield: 72%; m.p.: >300 °C; IR (KBr) *ν* (cm^−1^): 3317 (NH), 1629 (C=O), 1556, 1320 (NO_2_); MS (EI) *m/z*: 451 (M^+^, 0.49%); ^1^H-NMR (DMSO-d_6_, 300 MHz) *δ* (ppm): 7.46–8.77 (m, 15H, Ar-H + CH-methyne + NH-D_2_O exchangeable); Anal. Calcd. for C_23_H_15_Cl_2_N_3_O_3_ (451.04): C, 61.08; H, 3.34; N, 9.29%. Found: C, 61.29; H, 3.74; N, 9.35%.

##### 2.1.4.2. 6-(3,4-Dichlorophenyl)-4-(3-nitrobenzylidene)-1-phenyl-1,4-dihydropyridazin-3(2H)-one (5b)

Yield: 69%; m.p.: >300 °C; IR (KBr) *ν* (cm^−1^): 3456 (NH), 1671 (C=O), 1549, 1328 (NO_2_); MS (EI) *m/z*: 451 (M^+^, 1.56%); ^1^H-NMR (DMSO-d_6_, 300 MHz) *δ* (ppm): 7.16–8.49 (m, 15H, Ar-H + CH-methyne + NH-D_2_O exchangeable); Anal. Calcd. for C_23_H_15_Cl_2_N_3_O_3_ (451.04): C, 61.08; H, 3.34; N, 9.29%. Found: C, 61.28; H, 3.61; N, 9.35%.

##### 2.1.4.3. 6-(3,4-Dichlorophenyl)-4-(4-dimethylaminobenzylidene)-1-phenyl-1,4-dihydropyridazin-3(2H)-one (5c)

Yield: 43%; m.p.: >300 °C; IR (KBr) *ν* (cm^−1^): 3300 (NH), 1655 (C=O); MS (EI) *m/z*: 449 (M^+^, 1.63%); ^1^H-NMR (DMSO-d_6_, 300 MHz) *δ* (ppm): 2.86 (s,6H, N(CH_3_)_2_), 6.55–8.51 (m, 15H, Ar-H + CH-methyne + NH-D_2_O exchangeable); Anal. Calcd. for C_25_H_21_Cl_2_N_3_O (449.04): C, 66.67; H, 4.70; N, 9.33%. Found: C, 66.46; H, 5.12; N, 9.17%.

#### 2.1.5. General procedure for the synthesis of compounds 6a–c

To a solution of the furanones **2a–c** (0.01 mol) in absolute ethanol (20 ml), hydrazine hydrate (3.5 ml, 0.11 mol) was added. The reaction mixture was left at room temperature with occasional shaking until complete dissolving and poured onto ice water. The product obtained **6a–c** was filtered off, washed with hexane.

##### 2.1.5.1. 4-(3,4-Dichlorophenyl)-2-(2-nitrobenzylidene)-4-oxo-butanehydrazide (6a)

Yield: 65%; m.p.: 164–166 °C; IR (KBr) *ν* (cm^−1^): 3322 (NH), 3250 (NH_2_), 1703,1670 (2 C=O), 1521, 1343 (NO_2_); MS (EI) *m/z*: 395 (M + 2, 0.37%), 393 (M^+^, 0.47%), 364 (M + 2 – NHNH_2_, 21.29%), 362 (M – NHNH_2_, 31.7%); ^1^H-NMR (DMSO-d_6_, 300 MHz) *δ* (ppm): 3.17 (s, 2H, CH_2_), 4.55 (s, 2H, NH_2_, D_2_O-exchangable), 6.87 (s, 1H, NHCO, D_2_O-exchangable), 7.32–8.06 (m, 8H, Ar-H + CH-methyne); Anal. Calcd. for C_17_H_13_Cl_2_N_3_O_4_ (393.04): C, 51.91; H, 4.33; N, 10.69%. Found: C, 51.77; H, 4.51; N, 10.58%.

##### 2.1.5.2. 4-(3,4-Dichlorophenyl)-2-(3-nitrobenzylidene)-4-oxo-butanehydrazide (6b)

Yield: 58%; m.p.: 160–162 °C; IR (KBr) *ν* (cm^−1^): 3315 (NH), 3257 (NH_2_), 1697,1656 (2 C=O), 1528, 1360 (NO_2_); MS (EI) *m/z*: 393 (M^+^, 0.27%), 375 (M – H_2_O, 100%); ^1^H-NMR (DMSO-d_6_, 300 MHz) *δ* (ppm): 3.24 (s, 2H, CH_2_), 4.57 (s, 2H, NH_2_, D_2_O-exchangable), 6.92 (s, 1H, NHCO, D_2_O-exchangable), 7.35–8.32 (m, 8H, Ar-H + CH-methyne); Anal. Calcd. for C_17_H_13_Cl_2_N_3_O_4_ (393.04): C, 51.91; H, 4.33; N, 10.69%. Found: C, 51.75; H, 4.55; N, 10.74%.

##### 2.1.5.3. 4-(3,4-Dichlorophenyl)-2-(4-dimethylaminobenzylidene)-4-oxo-butanehydrazide (6c)

Yield: 56%; m.p.: 154–156 °C; IR (KBr) *ν* (cm^−1^): 3310 (NH), 3258 (NH_2_), 1751,1672 (2 C=O); MS (EI) *m/z*: 393 (M + 2, 9.38%), 391 (M^+^, 14.69%), 364 (M + 2 – NHNH_2_, 32.60%), 360 (M – NHNH_2_, 47.87%); ^1^H-NMR (DMSO-d_6_, 300 MHz) *δ* (ppm): 2.82 (s, 2H, CH_2_), 2.92 (s, 6H, N(CH_3_)_2_), 4.39 (s, 2H, NH_2_, D_2_O-exchangable), 6.73 (s, 1H, NHCO, D_2_O-exchangable), 6.67–7.61 (m, 8H, Ar-H + CH-methyne); Anal. Calcd. for C_19_H_19_Cl_2_N_3_O_2_ (391.01): C, 58.31; H, 4.86; N, 10.74%. Found: C, 58.66; H, 5.05; N, 10.77%.

#### 2.1.6. General procedure for the synthesis of compounds 7a–c

Method 1: A solution of hydrazides **6a–**c (0.01 mol) in HCl/AcOH (1:3) was refluxed for 3 h. The solid that separated after concentration and cooling was recrystallised from ethanol to obtain compounds **7a–c**.

Method 2: To a solution of the furanones **2a–c** (0.01 mol) in absolute ethanol (20 ml), hydrazine hydrate (3.5 ml, 0.11 mol) was added. The reaction mixture was refluxed for 4 h, then cooled and poured onto ice water. The product obtained **7a–c** was filtered off, washed with hexane.

##### 2.1.6.1. 3-(3,4-Dichlorophenyl)-5-[(2-nitropheny)l-methyl]-1H-pyridazin-6-one (7a)

Yield: 85%; m.p.: 224–226 °C; IR (KBr) *ν* (cm^−1^): 3187 (NH), 1655 (C=O), 1517 + 1343 (NO2); MS (EI) *m/z*: 377 (M + 2, 2.63%), 375 (M^+^, 3.8%), 330 (M + 2-NO_2_, 63.21%), 329 (M – NO_2_, 100%); ^1^H-NMR (DMSO-d_6_, 300 MHz) *δ* (ppm): 4.13 (s, 2H, CH_2_), 7.49–8.00 (m, 8H, Ar-H), 13.32 (s, 1H, NH-pyridazinone, D_2_O-exchangable); Anal. Calcd. for C_17_H_11_Cl_2_N_3_O_3_(375.04): C, 54.40; H, 2.93; N, 11.20%. Found: C, 54.72; H, 3.05; N, 11.08%.

##### 2.1.6.2. 3-(3,4-Dichlorophenyl)-5-[(3-nitropheny)l-methyl]-1H-pyridazin-6-one (7b)

Yield: 43%; m.p.: 257–259 °C; IR (KBr) *ν* (cm^−1^): 3203 (NH), 1653 (C=O), 1527 + 1346 (NO2); MS (EI) *m/z*: 377 (M + 2, 6.56%), 375 (M^+^, 10.06%); ^1^H-NMR (DMSO-d_6_, 300 MHz) *δ* (ppm): 4.01 (s, 2H, CH_2_), 7.59–8.25 (m, 8H, Ar-H), 13.34 (s, 1H, NH-pyridazinone, D_2_O-exchangable); Anal. Calcd. for C_17_H_11_Cl_2_N_3_O_3_(375.04): C, 54.40; H, 2.93; N, 11.20%. Found: C, 54.32; H, 2.83; N, 11.32%.

##### 2.1.6.3. 3-(3,4-Dichlorophenyl)-5-[(4-dimethylaminopheny)l-methyl]-1H-pyridazin-6-one (7c)

Yield: 87%; m.p.: 229–231 °C; IR (KBr) *ν* (cm^−1^): 3164 (NH), 1651 (C=O); MS (EI) *m/z*: 377 (M + 4, 12.06%), 375 (M + 2, 67.79%), 373 (M^+^, 100%); ^1^H-NMR (DMSO-d_6_, 300 MHz) *δ* (ppm): 2.81 (s, 6H, N(CH_3_)_2_), 3.70 (s, 2H, CH_2_), 6.62–8.06 (m, 8H, Ar-H), 13.19 (s, 1H, NH-pyridazinone, D_2_O-exchangable); Anal. Calcd. for C_19_H_17_Cl_2_N_3_O (373.09): C, 61.13; H, 4.56; N, 11.26%. Found: C, 61.33; H, 4.54; N, 10.99%.

#### 2.1.7. General procedure for the synthesis of compounds 8a–c

To a solution of the hydrazides **6a–c** (0.01 mol) in dry benzene (20 ml), benzoyl chloride (1.4 ml, 0.01 mol) was added. The reaction mixture was heated under reflux for 2 h. The solvent was evaporated, and the solid obtained was washed thoroughly with ethanol, drained, and recrystallised from hexane to give compounds **8a–c**.

##### 2.1.7.1. 2-Benzoyl-5-(2-nitrobenzylidene)-3-(3,4-dichlorophenyl)-1H-pyridazin-6-one (8a)

Yield: 84%; m.p.: 222–224 °C; IR (KBr) *ν* (cm^−1^): 3275 (NH), 1740,1660 (2 C=O),1512, 1343 (NO2); MS (EI) *m/z*: 481 (M + 2, 1.17%), 479 (M^+^, 1.69%), 105 (benzoyl, 100%); ^1^H-NMR (DMSO-d_6_, 300 MHz) *δ* (ppm): 6.69 (s, 1H, CH-methyne),7.48–8.20 (m, 13H, Ar-H), 11.30 (s, 1H, NH, D_2_O-exchangable); Anal. Calcd. for C_24_H_15_Cl_2_N_3_O_4_ (479.04): C, 60.02; H, 3.15; N, 8.75%. Found: C, 60.34; H, 3.01; N, 8.94%.

##### 2.1.7.2. 2-Benzoyl-5-(3-nitrobenzylidene)-3-(3,4-dichlorophenyl)-1H-pyridazin-6-one (8b)

Yield: 40%; m.p.: 150–152 °C; IR (KBr) *ν* (cm^−1^): 3250 (NH), 1717,1677 (2 C=O),1523, 1350 (NO2); MS (EI) *m/z*: 479 (M^+^, 2.82%), 105 (benzoyl, 100%); ^1^H-NMR (DMSO-d_6_, 300 MHz) *δ* (ppm): 7.03–8.59 (m, 13H, Ar-H + CH-methyne), 11.34 (s, 1H, NH, D_2_O-exchangable); Anal. Calcd. for C_24_H_15_Cl_2_N_3_O_4_ (479.04): C, 60.02; H, 3.15; N, 8.75%. Found: C, 60.12; H, 3.56; N, 8.91%.

##### 2.1.7.3. 2-Benzoyl-5-(4-dimethylaminobenzylidene)-3-(3,4-dichlorophenyl)-1H-pyridazin-6-one (8c)

Yield: 37%; m.p.: 229–231 °C; IR (KBr) *ν* (cm^−1^): 3225 (NH), 1687,1664 (2 C=O); MS (EI) *m/z*: 479 (M + 2, 33.09%), 474 (M^+^, 49.44%), 105 (benzoyl, 100%); ^1^H-NMR (DMSO-d_6_, 300 MHz) *δ* (ppm): 3.03 (s, 6H, N(CH_3_)_2_), 6.76–8.01 (m, 14H, Ar-H + CH-methyne), 11.17 (s, 1H, NH, D_2_O-exchangable); Anal. Calcd. for C_26_H_21_Cl_2_N_3_O_2_ (477.09): C, 65.28; H, 4.42; N, 8.78%. Found: C, 65.31; H, 4.68; N, 8.23%.

#### 2.1.8. General procedure for the synthesis of compounds 9a–c

A solution of hydrazides **6a–c** (0.01 mol) and carbon disulphide (3 ml) in pyridine (10 ml) was refluxed for 3 h. The reaction mixture was left to cool at room temperature and poured onto ice water; the product obtained was filtered, washed with water and recrystallised from ethanol to give compounds **9a–c**.

##### 2.1.8.1. 1-(3,4-Dichlorophenyl)-4-(2-nitrophenyl)-3-(2-thioxo-4,5-dihydro-1,3,4-oxadiazol-5-yl)but-3-en-1-one (9a)

Yield: 40%; m.p.: 214–216 °C; IR (KBr) *ν* (cm^−1^): 3196 (NH), 1653 (C=O),1504, 1340 (NO_2_), 1254 (C=S); MS (EI) *m/z*: 435 (M^+^, 0.31%); ^1^H-NMR (DMSO-d_6_, 300 MHz) *δ* (ppm): 4.13 (s, 2H, CH_2_), 7.36–8.00 (m, 8H, Ar-H + CH-methyne), 13.32 (s, 1H, NH, D_2_O-exchangable); Anal. Calcd. for C_18_H_11_Cl_2_N_3_O_4_S (435.04): C, 49.56; H, 2.54; N, 9.63%. Found: C, 49.38; H, 2.76; N, 9.54%.

##### 2.1.8.2. 1-(3,4-Dichlorophenyl)-4-(3-nitrophenyl)-3-(2-thioxo-4,5-dihydro-1,3,4-oxadiazol-5-yl)but-3-en-1-one (9b)

Yield: 58%; m.p.: 242–244 °C; IR (KBr) *ν* (cm^−1^): 3195 (NH), 1653 (C=O),1514, 1346 (NO_2_), 1238 (C=S); MS (EI) *m/z*: 435 (M^+^, 0.07%); ^1^H-NMR (DMSO-d_6_, 300 MHz) *δ* (ppm): 3.99 (s, 2H, CH_2_), 7.38–8.57 (m, 8H, Ar-H + CH-methyne), 13.32 (s, 1H, NH, D_2_O-exchangable); Anal. Calcd. for C_18_H_11_Cl_2_N_3_O_4_S (435.04): C, 49.56; H, 2.54; N, 9.63%. Found: C, 49.37; H, 2.34; N, 9.41%.

##### 2.1.8.3. 1-(3,4-Dichlorophenyl)-4-(4-dimethylaminophenyl)-3-(2-thioxo-4,5-dihydro-1,3,4-oxadiazol-5-yl)but-3-en-1-one (9c)

Yield: 89%; m.p.: 162–164 °C; IR (KBr) *ν* (cm^−1^): 3200 (NH), 1655 (C=O), 1230 (C=S); MS (EI) *m/z*: 433 (M^+^, 1.4%); ^1^H-NMR (DMSO-d_6_, 300 MHz) *δ* (ppm): 2.84 (s, 6H, N(CH_3_)_2_), 3.72 (s, 2H, CH_2_), 6.65–8.59 (m, 8H, Ar-H + CH-methyne), 13.22 (s, 1H, NH, D_2_O-exchangable); Anal. Calcd. for C_20_H_17_Cl_2_N_3_O_2_S (433.04): C, 55.31; H, 3.95; N, 9.67%. Found: C, 55.64; H, 3.47; N, 9.21%.

### 2.2. Biological evaluation of anti-inflammatory activity

#### 2.2.1. Cyclooxygenase (COX-1 and COX-2) inhibition assay

Tested compounds were dissolved in DMSO. Each compound was tested in triplicates using a COX inhibitory screening assay kit according to the manufacturer (Cayman test kit-560131, Cayman Chemical, Ann Arbor, MI). The COX inhibitor screening assay depends on direct measurement of the amount of PG2α produced in the COX reaction. Celecoxib, rofecoxib, and indomethacin were used as the positive control for inhibition of COX-1 and COX-2. An aliquot of 20 µl of each test compound or solvent (100% initial activity) was added to 950 µl of Reaction Buffer (0.1 M Tris–HCl, pH 8.0, containing 5 mM EDTA and 2 mM phenol), 10 µl of haem, and 10 µl of COX-1 or COX-2, then incubated with the enzymes at 37 °C for 10 min. The reaction was initiated by addition of 10 µl AA to all the test tubes and incubation at 37 °C for an additional 2 min. Enzyme catalysis was terminated by addition of 50 µl 1 M HCl and 100 µl of the saturated stannous chloride solution. The PGs are quantified by enzyme immunoassay (EIA) at 410 nm.

#### 2.2.2. Lipoxygenase (15-LOX) inhibition assay

The experiment was performed in triplicates using Cayman’s LOX inhibitor screening assay kit (Cayman test kit-760700, Cayman Chemical, Ann Arbor, MI) according to the manufacturer’s protocol. DMSO was used as 100% initial activity and Quercetin was used as the positive control. Briefly, in a 96-well plate, 10 µl of each test compound (dissolved in DMSO) or vehicle were pre-incubated with 90 µl of 15-LOX enzyme. The reaction was started by addition of 10 µl of substrate (AA) and the plate was shaken for at least 5 min. Then, 100 µl of chromogen supplied with the kit was added to each well to stop the enzymatic reaction and develop the colour. The absorbance was measured at 490 nm using microplate reader.

#### 2.2.3. *TNF-*α *[biotinylated] inhibition assay*

The assay was performed in triplicates using TNFR2: TNF-α [biotinylated] Inhibitor Screening Assay Kit (BPS bioscience, San Diego, CA, Catalog #79756), following the manufacturer’s protocol. First, TNFR2 is coated on a 96-well plate. Next, biotinylated TNF-α is incubated with TNFR2 on the plate. Finally, the plate was treated with streptavidin-HRP followed by addition of an HRP substrate to produce chemiluminescence, which can be measured using a chemiluminescence reader.

## 3. Results and discussion

### 3.1. Chemistry

Overall, 22 new compounds and two reported compounds were synthesised as revealed in Schemes A and B. 2-Furanone derivatives **2a–c** were synthesised from 4-oxobutanoic derivative **1** by reacting with aromatic aldehydes in acetic anhydride following modified Perkin reaction conditions^23^^,^^27–29^^,^^35^.

The required 4-oxobutanoic derivative **1** was prepared by condensing dry dichlorobenzene with succinic anhydride in presence of anhydrous aluminium chloride, following Friedel–Crafts acylation reaction conditions.

As revealed in Scheme A, 2-furanone derivatives **2a–c** proved to be useful precursors in the synthesis of several heterocyclic and non-heterocyclic derivatives. When they were allowed to react, separately, with ammonium acetate; pyrrol-2-one derivatives **3a–c** were produced. 4-oxobutanamides **4a–c** were prepared by refluxing 2-furanones **2a–c** with benzylamine and 1-phenylpyridazinone **5a–c** were also prepared from 2-furanones by refluxing with phenyl hydrazine according to the reported procedure^29^^,^^31^.

Pyridazinone derivatives **7a–c** can be prepared by first stirring of 2-furanone derivatives **2a–c** with hydrazine hydrate to obtain hydrazide derivatives **6a–c** which can be cyclised by refluxing in HCl to obtain the desired pyridazinone derivatives **7a–c**. They can be prepared directly by refluxing 2-furanone derivatives **2a–c** with hydrazine hydrate.

Hydrazide derivatives **6a–c** were refluxed with benzoyl chloride to obtain 2-benzoylpyridazinone derivatives **8a–c**.

Finally, oxadiazole-thione derivatives **9a–c** were prepared *via* refluxing hydrazide derivatives **6a–c** with carbon disulphide in pyridine^2^^,^^22^^,^^23^^,^^27^^,^^49^ as revealed in Scheme B.

### 3.2. Anti-inflammatory activity results

Inflammation is a protective defence response of the body. However, during inflammation, several pathological changes occur, which involve the release of common mediators of inflammation like PGs, histamine, nitric oxide, leukotrienes (LTB4), platelet-activation factor, lipoxins, and cytokines^50^. The inflammatory response must be terminated; using anti-inflammatory compounds, when no more needed to prevent avoidable harmful biological processes^51^. Inhibition of eicosanoids generation, in addition to the release of the pro-inflammatory cytokine; TNF-α from macrophages are used for *in vitro* inflammation tests^52^.

Thus, the newly synthesised compounds were tested for their anti-inflammatory activity against isozymes COX-1 and COX-2, which were determined by the COX-catalysed transformation of AA into PGH2 that was reduced to PGF2α and detected by the EIA^53^, as shown in Table 1.

**Table 1.** COX-1, COX-2, and 15-LOX enzymes inhibition activities of the synthesised compounds.

Compound number	COX1	COX 2	Selectivity indexes	LOX
	IC_50_ (µm)	IC_50_ (µm)	(SI)	IC_50_ (µm)
**2a**	4.53 ± 0.12	0.35 ± 0.01	12.94	5.10 ± 0.10
**2b**	6.97 ± 0.12	0.28 ± 0.01	24.89	4.40 ± 0.10
**2c**	3.93 ± 0.06	0.42 ± 0.02	9.36	6.13 ± 0.12
**3a**	8.37 ± 0.12	0.19 ± 0.01	44.05	2.33 ± 0.12
**3b**	6.87 ± 0.12	0.39 ± 0.01	17.62	1.87 ± 0.15
**3c**	5.93 ± 0.15	0.34 ± 0.01	17.44	2.21 ± 0.98
**4a**	11.07 ± 0.15	0.05 ± 0.00	221.40	5.47 ± 0.12
**4b**	13.33 ± 0.15	0.06 ± 0.00	222.17	5.87 ± 0.06
**4c**	11.90 ± 0.10	0.05 ± 0.00	238.00	4.67 ± 0.06
**5a**	12.73 ± 0.12	0.06 ± 0.00	212.17	2.70 ± 0.95
**5b**	13.00 ± 0.10	0.04 ± 0.00	325.00	2.43 ± 0.12
**5c**	11.73 ± 0.12	0.09 ± 0.00	130.33	2.97 ± 0.12
**6a**	9.67 ± 0.06	0.09 ± 0.00	107.44	2.83 ± 0.06
**6b**	10.07 ± 0.15	0.08 ± 0.00	125.88	2.47 ± 0.06
**6c**	8.83 ± 0.06	0.10 ± 0.01	88.30	3.17 ± 0.06
**7a**	7.03 ± 0.15	0.17 ± 0.01	41.35	5.47 ± 0.12
**7b**	6.67 ± 0.12	0.25 ± 0.01	26.68	5.87 ± 0.06
**7c**	8.33 ± 0.06	0.12 ± 0.01	69.42	4.67 ± 0.06
**8a**	10.77 ± 0.15	0.07 ± 0.00	153.86	2.33 ± 0.51
**8b**	12.67 ± 0.12	0.04 ± 0.00	316.75	2.47 ± 0.06
**8c**	13.07 ± 0.12	0.04 ± 0.00	326.75	3.17 ± 0.06
**9a**	9.43 ± 0.12	0.28 ± 0.01	33.68	2.07 ± 0.15
**9b**	10.43 ± 0.12	0.08 ± 0.00	130.38	1.63 ± 0.15
**9c**	8.93 ± 0.06	0.22 ± 0.01	40.59	2.00 ± 0.17
Celecoxib	14.70 ± 0.06	0.05 ± 0.00	326.67	ND
Rofecoxib	14.50 ± 0.10	0.03 ± 0.00	580.00	ND
Indomethacin	0.10 ± 0.00	0.08 ± 0.00	1.25	ND
Quercetin	ND	ND	ND	3.34 ± 0.05

Data are presented as mean ± SD of three experiments.

ND: not determined; SI = IC_50_ COX-1/COX-2 ratios.

Compound **2c** was the most potent COX-1 inhibitor (IC_50_ 3.93 ± 0.06 µM) among test compounds, being 39 times less active than the reference drug indomethacin (IC_50_=0.10 µM) indicating the selective COX-2 inhibition activity of all test compounds. Pyridazinone derivatives **5b, 8b,** and **8c** were the most potent selective COX-2 inhibitors (IC_50_=0.04 µM for the three compounds), being superior to the reference drug (celecoxib IC_50_=0.05 µM). While amide derivatives; **4a–c,** hydrazides; **6a, 6b,** N-phenyl and N-benzoylpyridazinones; **5a, 5c, 8a** and oxadiazole; **9b** (IC_50_=0.05–0.09 µM range), showed comparable activity to celecoxib as selective COX-2 inhibitors. These results indicate the high activity of hydrazides, pyridazinones, and oxadiazole; which is in consistent with previously reported activities of similar scaffolds as selective COX-2 inhibitors^37^^,^^42^^,^^54–57^.

Studies showed that soybean LOX enzyme has a wide substrate specificity, and that the oxygenation sites for the soybean LOX has been demonstrated to be at C-13 of α-linolenic acid and C-15 of AA^58^. Therefore, the soybean LOX is most like the mammalian 15-LOX^59^.

Consequently, the *in vitro* inhibitory effect of all synthesised compounds against 15-LOX was determined using a lipoxygenation reaction to transform AA into hydroperoxides, which were detected by the addition of a chromogen, whose results are displayed in Table 1^60^.

Results also revealed that all the synthesised derivatives of pyrrolones **3a–c**, hydrazides **6a–c**, N-phenyl and N-benzoylpyridazinones; **5a–c**, **8a–c,** and oxadiazoles **9a–c** inhibited 15-LOX (IC_50_ =1.63 − 3.17 µM range), **9b** being the most potent 15-LOX inhibitor (IC_50_=1.63 ± 0.15 µM), compared to the positive control Quercetin (IC_50_=3.34 µM). However, furanones **2a–c**, amides **4a–c**, and pyridazinones **7a–c** exhibited lower 15-LOX inhibitory effect.

By evaluating the COX and 15-LOX inhibition results, three compounds namely 6–(3,4-dichlorophenyl)-4–(3-nitrobenzylidene)-1-phenyl-1,4-dihydropyridazin-3(2H)-one **(5b)**, 2-benzoyl-5–(3-nitrobenzylidene)-3–(3,4-dichlorophenyl)-1H-pyridazin-6-one **(8b),** and 2-benzoyl-5–(4-dimethylaminobenzylidene)-3–(3,4-dichlorophenyl)-1H-pyridazin-6-one **(8c)** seemed to be the best candidates as a COX-2/15-LOX dual inhibitors, which is a current subject of interest in the development of anti-inflammatory agents^60^. This declares the expected activity of N-phenyl and N-benzoylpyridazinones derivatives as reported for some other derivatives with the same scaffold^61^ . On the other hand, compounds **5a, 5c, 6a, 6b, 8a,** and **9b** exhibited a little lower COX-2 inhibition but still show high dual COX-2/15-LOX inhibition activity.

Moreover, the ability to inhibit TNF-α, which is a potent pro-inflammatory chemokine was tested; results are shown in Table 2. The 15-LOX pathway was reported to induce inflammation through increased expression of IL-6, IFN-γ, IL-12, and TNF-α^62^.

**Table 2.** Inhibitory effects of tested compounds against TNF-α.

Compound number	TNF-α
	IC_50_ (nM)
**2a**	7.47 ± 0.12
**2b**	7.77 ± 1.68
**2c**	8.20 ± 0.10
**3a**	5.93 ± 0.15
**3b**	8.27 ± 0.12
**3c**	6.77 ± 0.12
**4a**	4.77 ± 0.12
**4b**	3.53 ± 0.12
**4c**	5.47 ± 0.12
**5a**	4.93 ± 0.06
**5b**	3.27 ± 0.12
**5c**	3.57 ± 0.12
**6a**	5.87 ± 0.12
**6b**	4.10 ± 0.10
**6c**	6.17 ± 0.06
**7a**	8.53 ± 0.12
**7b**	8.17 ± 0.06
**7c**	7.57 ± 0.06
**8a**	3.83 ± 1.44
**8b**	3.47 ± 0.06
**8c**	2.90 ± 0.10
**9a**	5.57 ± 0.06
**9b**	4.33 ± 0.12
**9c**	6.27 ± 0.15
Certolizumab	6.70 ± 0.12

Data are presented as mean ± SD of three experiments.

Sixteen compounds of our newly synthesised tested compounds; pyrrolone **3a,** amides **4a–c**, N-phenylpyridazinones **5a–c**, hydrazides **6a–c**, N-benzoylpyridazinones **8a–c**, and oxadiazoles **9a–c** showed higher activities (IC_50_ = 2.90 − 6.27 µM range) than the used reference inhibitor Certolizumab (IC_50_=6.70 µm). While compound **3c** showed comparable activity with IC_50_=6.77 µM. Compound **8c** showed the lowest IC_50_ value for TNF-α inhibition, indicating that this activity may be a consequence of 15-LOX inhibition.

**Figure 2.**
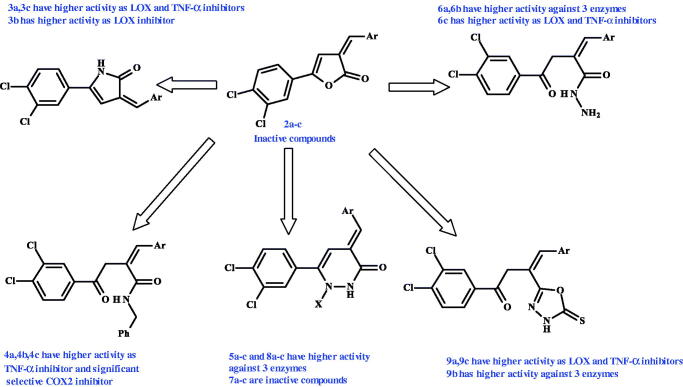
Structure–activity relationship of the tested compounds against the tested enzymes.

To analyse the structure–activity relationship (SAR) of the tested compounds from the previous results, it is obvious that our three newly synthesised furanones are totally inactive towards all the tested enzymes. But upon their conversion to other heterocyclic and non-heterocyclic compounds the biological activities appear as revealed in Figure 2 and explained as follows:

3 Pyrrolone derivatives **3a–c** acquired high activity as LOX-inhibitors, only one of them having 3-nitrobenzylidene substituent **(3b)** showed high activity as TNF-α inhibitor. They still have no significant activities as COX-2 inhibitors.

Upon ring opening and formation of amide derivatives **4a–c**, a high TNF-α inhibition appears with significant selective COX-2 inhibition, but no significant LOX-inhibition observed.

Also, ring opening and hydrazides formation in **6a–c** showed activity against 15-LOX and TNF-α, while **6a,b** having 2-nitrobenzylidene and 3-nitrobenzylidene substituents showed high activity as selective COX-2 inhibitors.

Conversion of furanones to pyridazinines **7a–c** did not affect their biological activities as they were still inactive. But formation of N-phenylpyridazinones **5a–c** and N-benzoylpyridazinones **8a–c** was a perfect pathway for highly potent derivatives against all the tested enzymes with desirable dual COX-2/15-LOX inhibition activities for compounds **5b**, **8b**, and **8c** indicating the important influence of presence of 3-nitrobenzylidene substituent in both pyridazinones (**5b, 8b**) and 4-dimethylaminobenzylidene in N-benzoylpyridazinone (**8c**).

Finally, three oxadiazole derivatives **9a–c** showed high activity against 15-LOX and TNF-α, while two derivatives, having 2-nitrobenzylidene and 4-dimethylaminobenzylidene substituents, **9a** and **9c,** respectively, showed high activity as selective COX-2 inhibitors.

**Scheme A.**
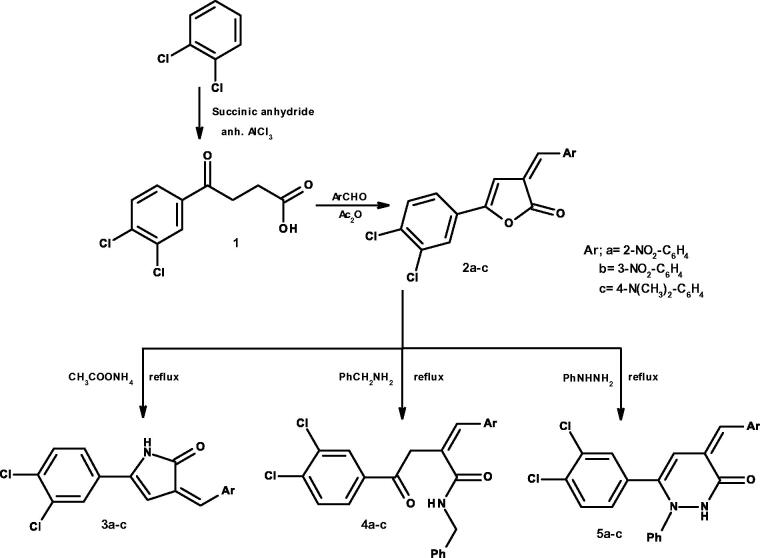
Synthesis of compounds **2a–c** to **5a–c.**

**Scheme B.**
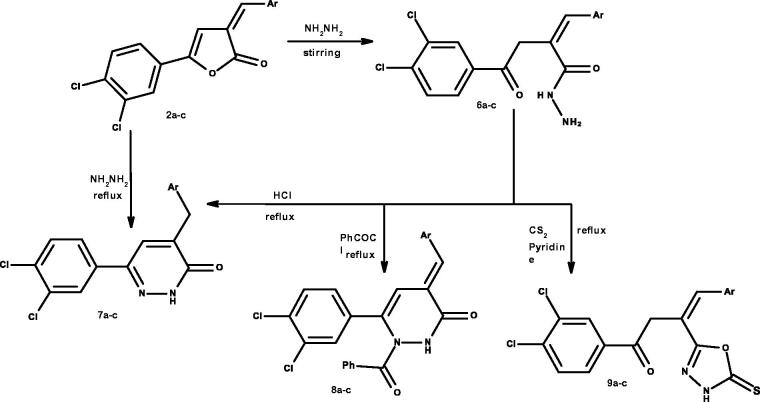
Synthesis of compounds **6a–c** to **9a–c.**

## Conclusion

In conclusion, as presented in this study, novel 2-furanone derivatives were synthesised and used to prepare novel hydrazides, 2-pyrrolone, 2-pyridazinone, and oxadiazole derivatives. All the synthesised compounds were investigated for their anti-inflammatory activity; the biological results revealed that N-phenylpyridazinone **5b,** N-benzoylpyridazinones **8b** and **8c** showed promising activity as dual COX-2/15-LOX inhibitors along with high TNF-α inhibition activity. Thus, these compounds might be promising anti-inflammatory candidates and may need further studies to be used clinically.

## Supplementary Material

Supplemental Material
