# Supplementary material for: Utility of novel 2-furanones in synthesis of other heterocyclic compounds having anti-inflammatory activity with dual COX2/LOX inhibition
Source: J Enzyme Inhib Med Chem. 2021 May 6;36(1):977–86. doi: 10.1080/14756366.2021.1908277 (PMC8118430; doi:10.1080/14756366.2021.1908277)
Supplement: Supplemental Material [file IENZ_A_1908277_SM1304.pdf]

Utility of Novel 2-Furanones in Synthesis of Other Heterocyclic Compounds and Evaluation of Their Anti-Inflammatory Activity

Rania H. Abd El-Hameed[*a], Shahenda Mahgoub[b], Hend M. El-Shanbaky [a], Mosaad S. Mohamed[a] , Sahar A. Ali[b]

a) Pharmaceutical Organic Chemistry Department, Faculty of pharmacy, Helwan University, Helwan, Cairo, Egypt

b) Biochemistry Department, Faculty of pharmacy, Helwan University, Helwan, Cairo, Egypt

****Correspondence**: **Rania Helmy Abd El-Hameed**,

Pharmaceutical organic chemistry department, Faculty of Pharmacy, Helwan University, Ain-Helwan, Helwan, Cairo. Egypt.

Tel (Fax); 00202-2554-1601 E-mail; zeiadomar@yahoo.com Postal code: 11795

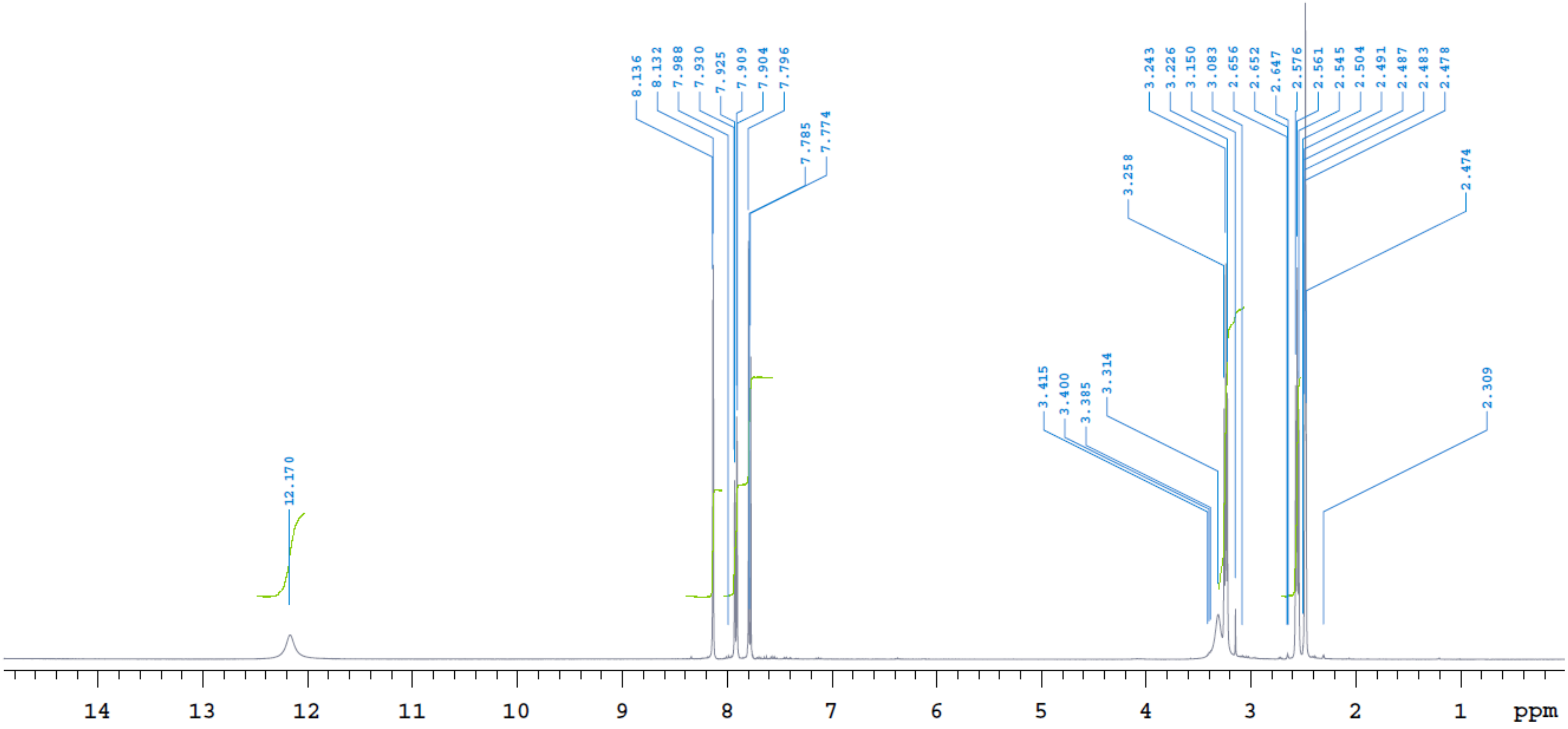

^{1}H-NMR spectrum of compound 1

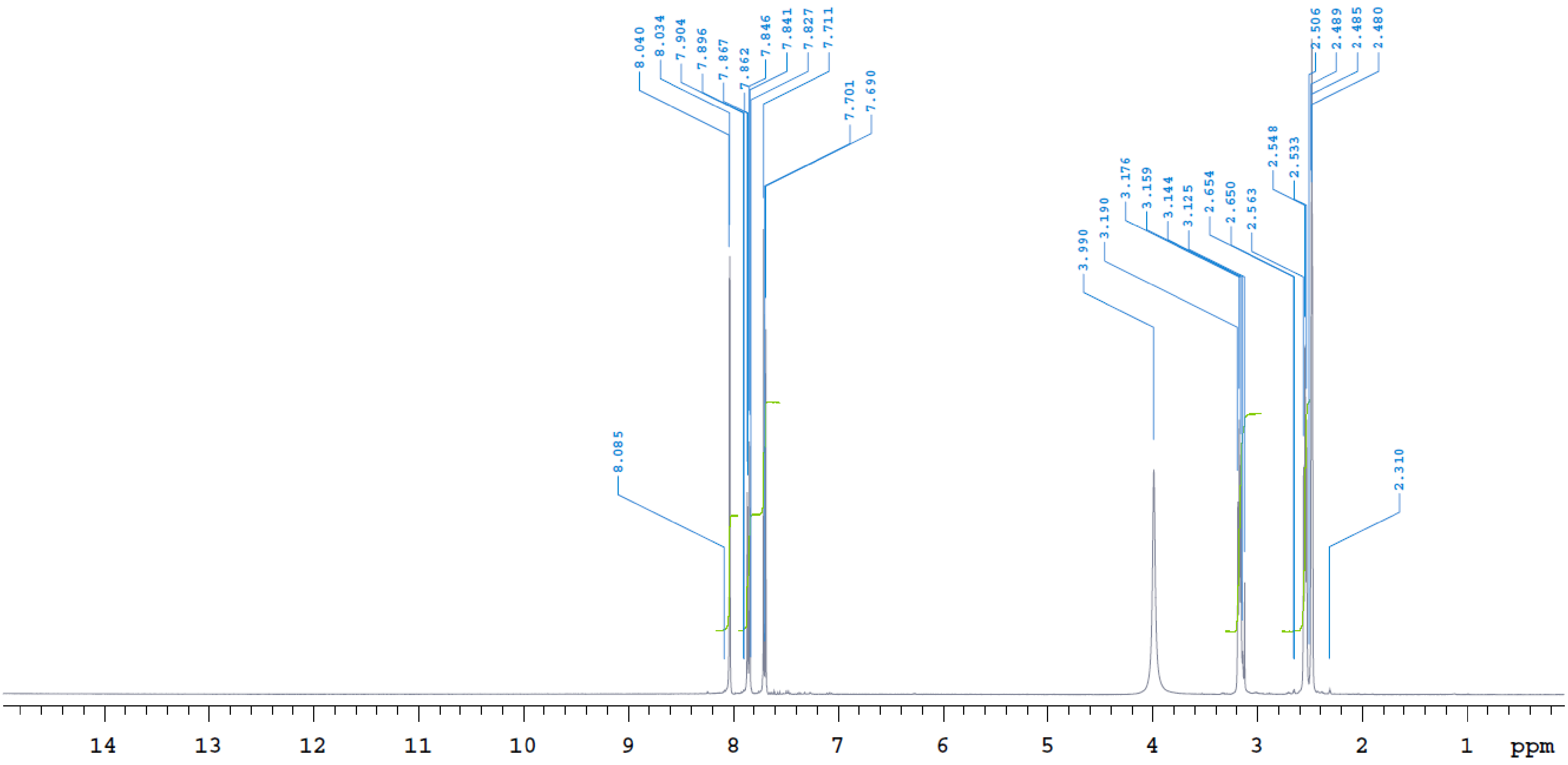

^{1}H-NMR spectrum of compound 1- D_2O

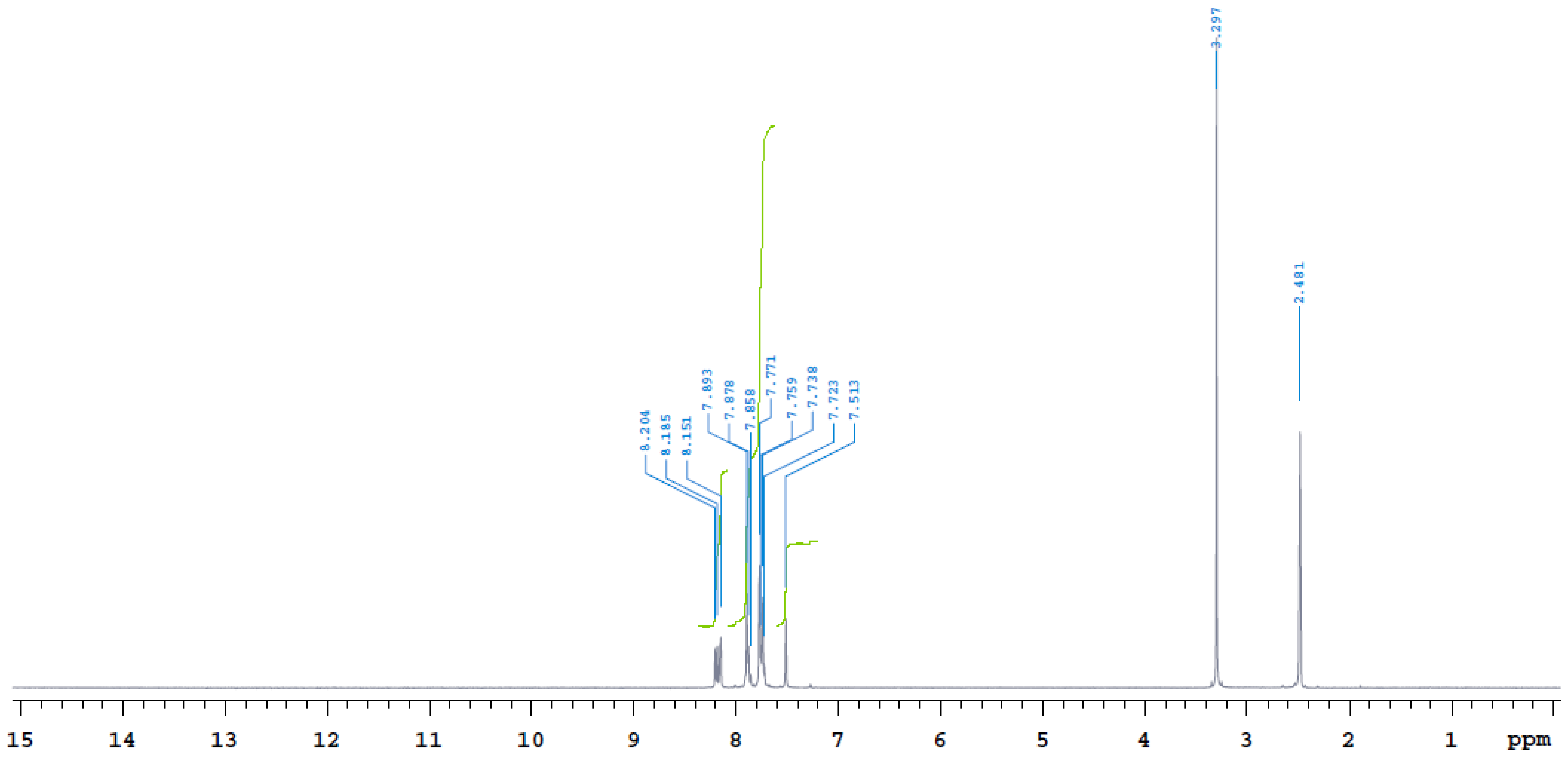

^{1}H-NMR spectrum of compound 2a

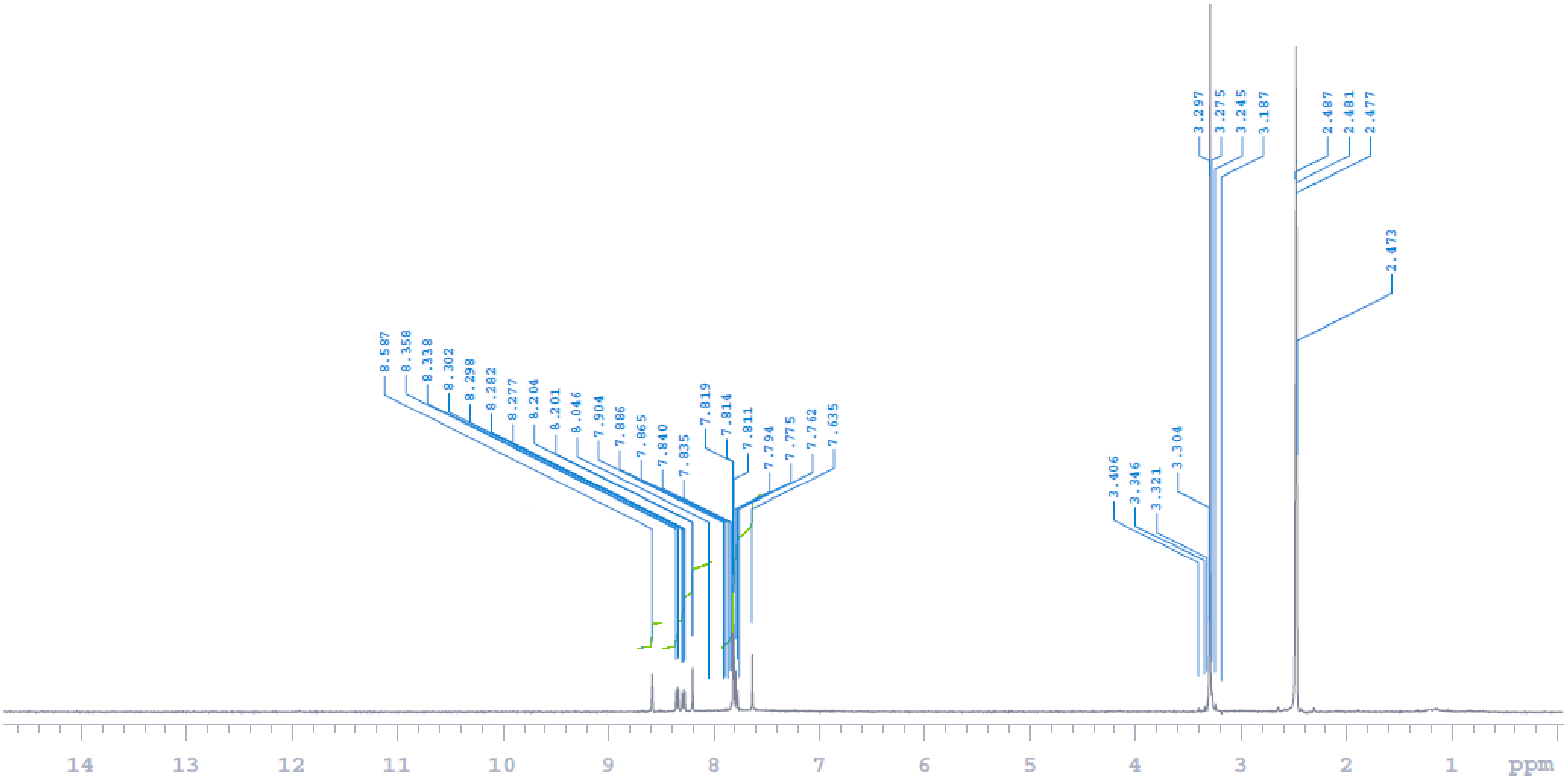

^{1}H-NMR spectrum of compound 2b

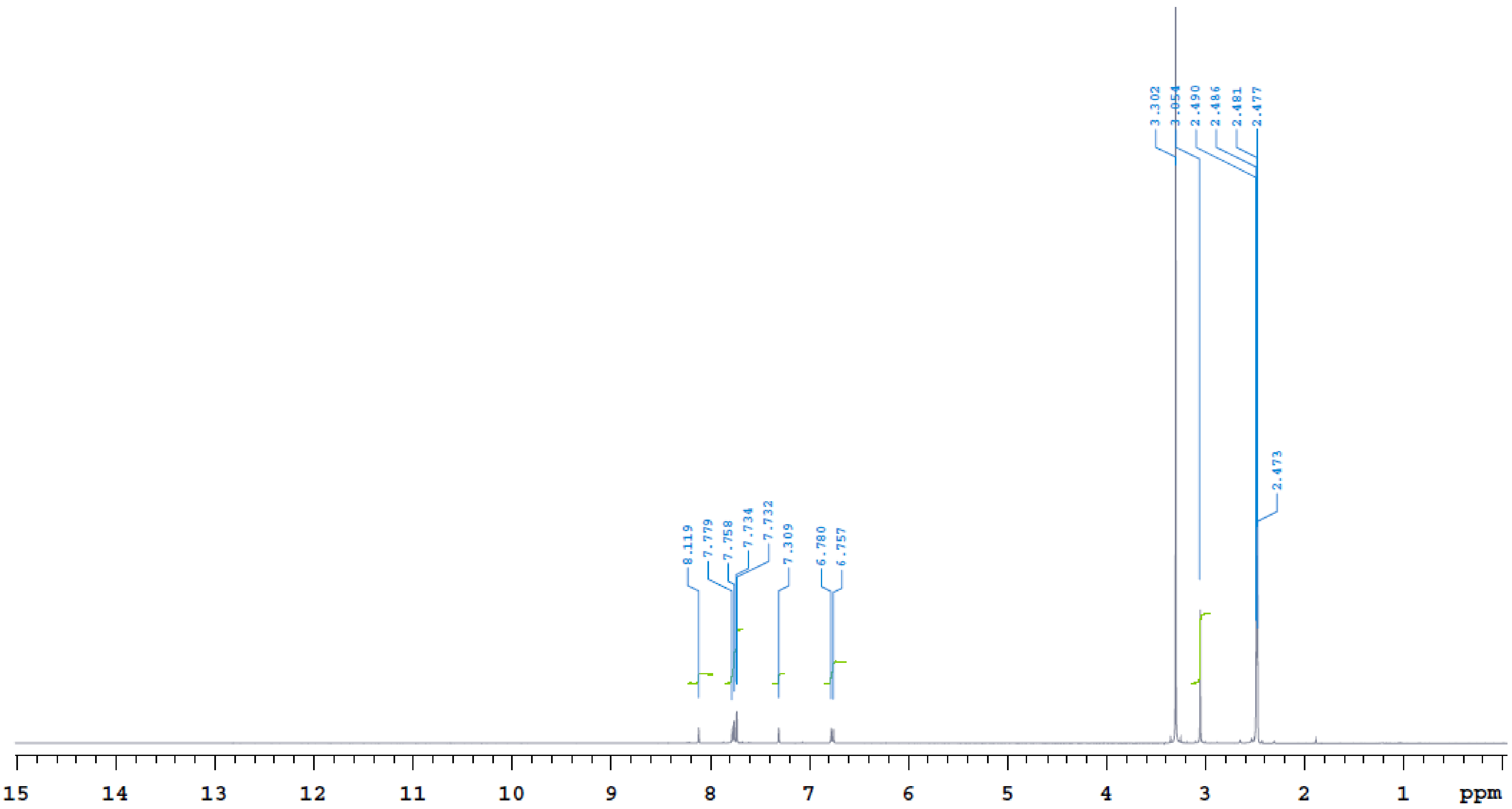

^{1}H-NMR spectrum of compound 2c

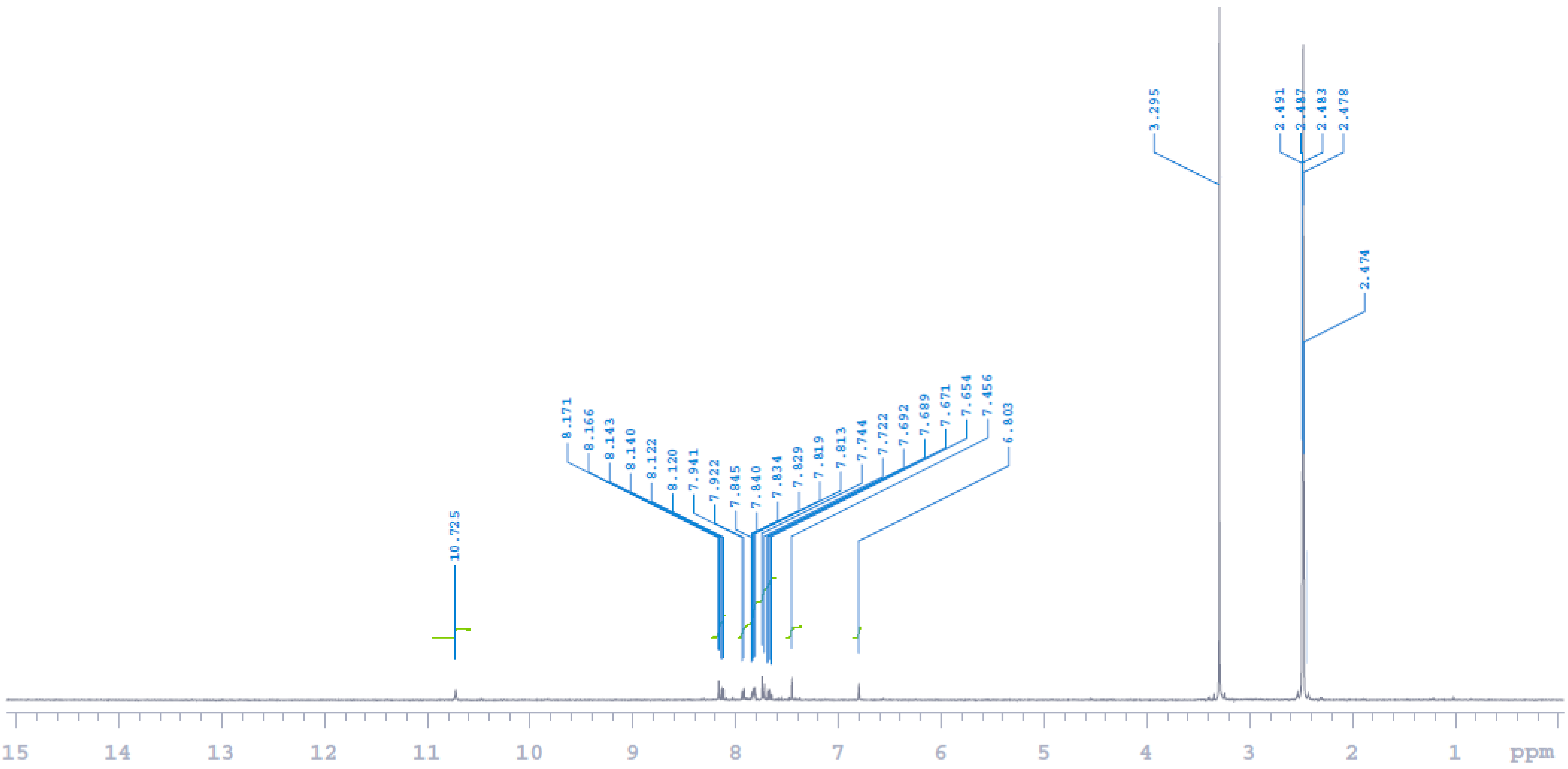

^{1}H-NMR spectrum of compound 3a

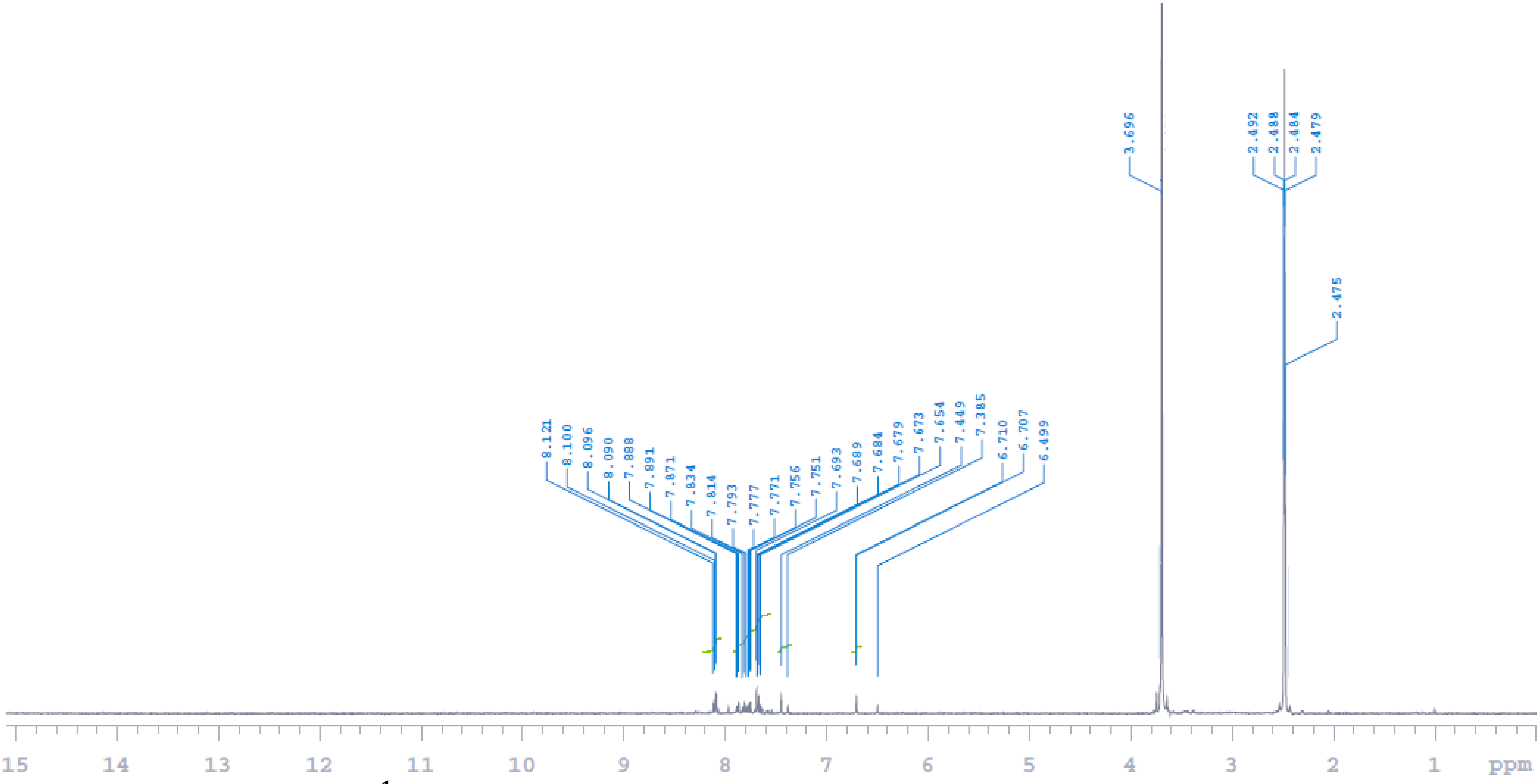

^{1}H-NMR spectrum of compound 3a- D_2O

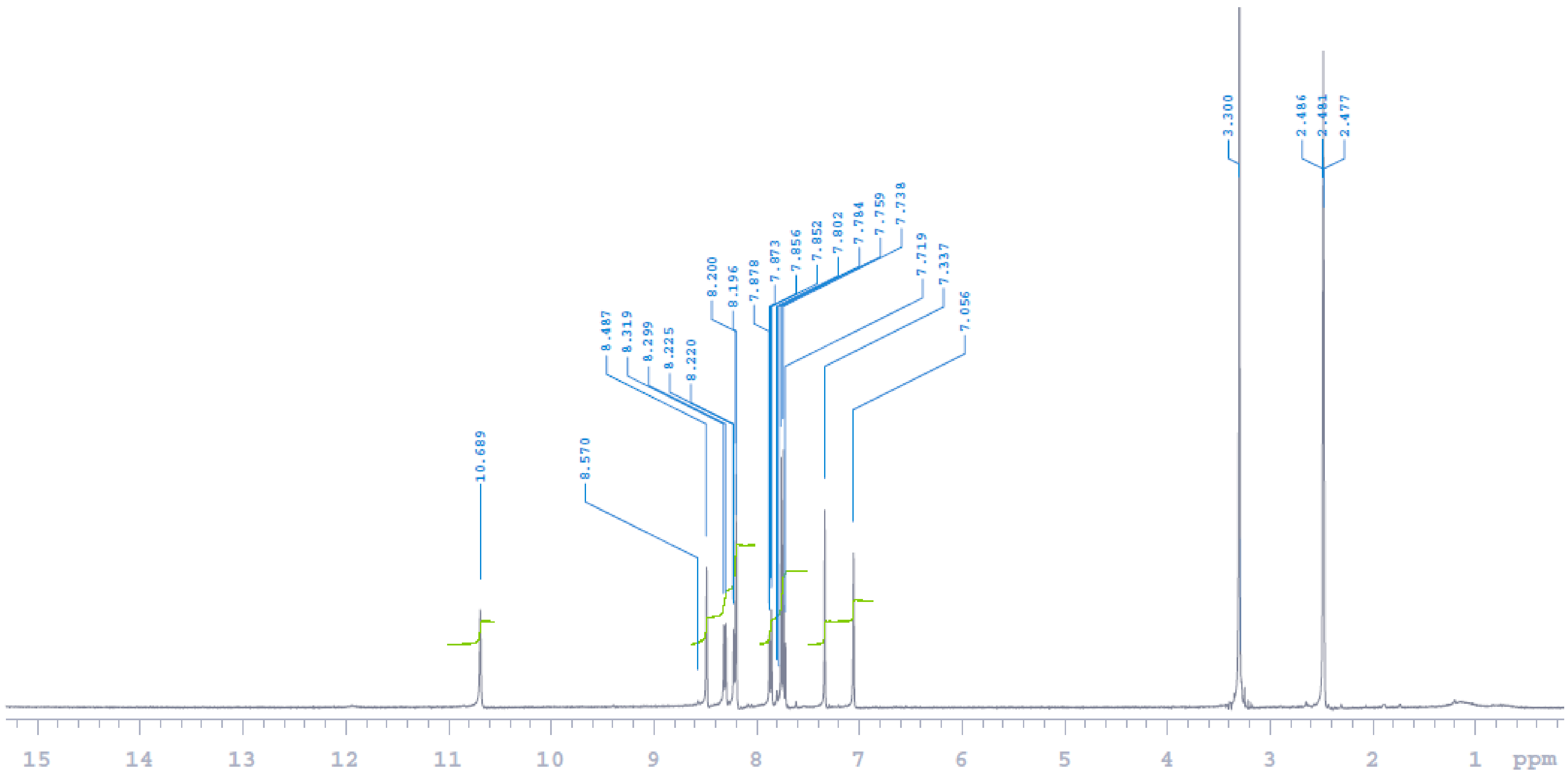

^{1}H-NMR spectrum of compound 3b

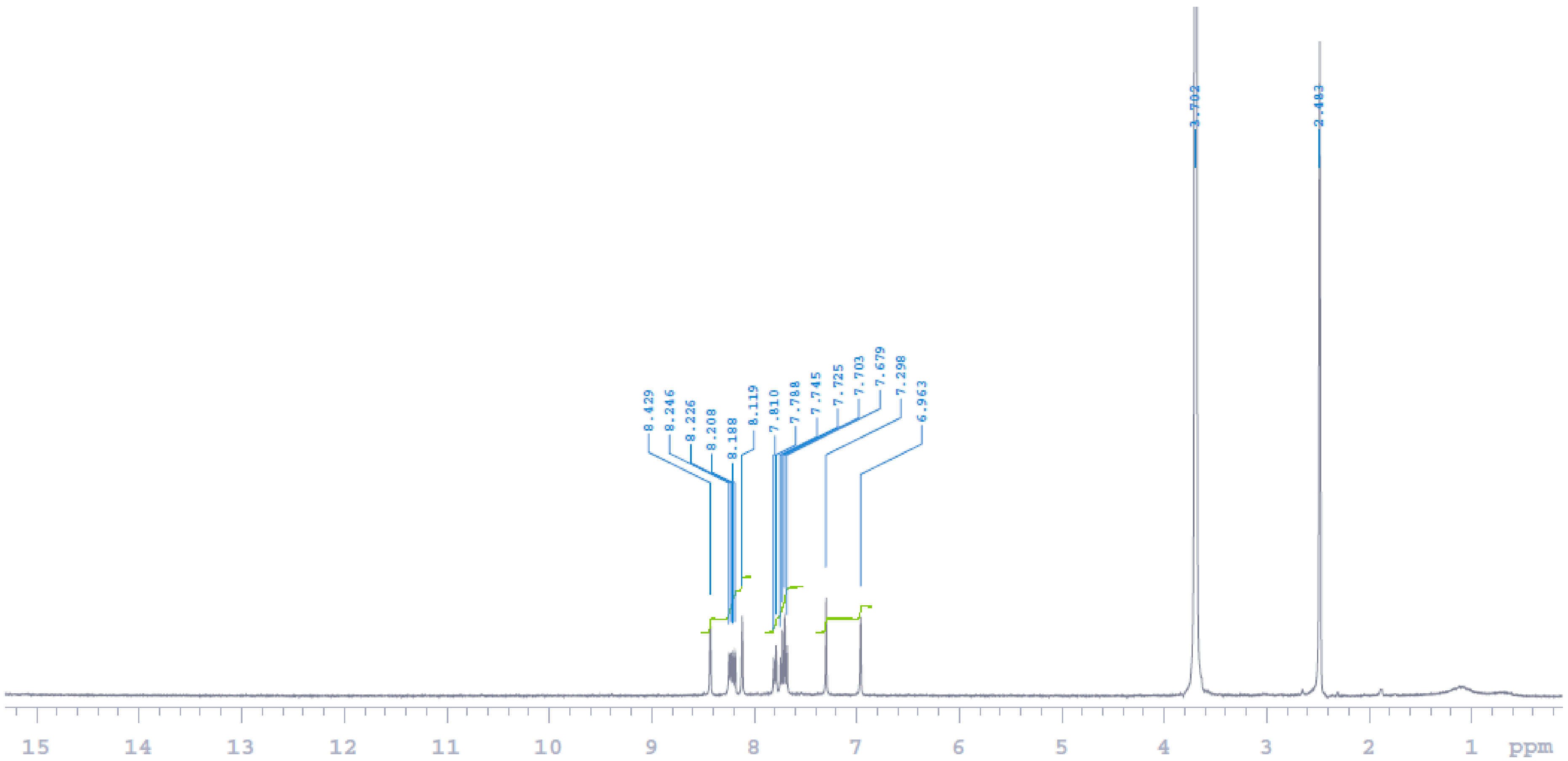

^{1}H-NMR spectrum of compound 3b- D_2O

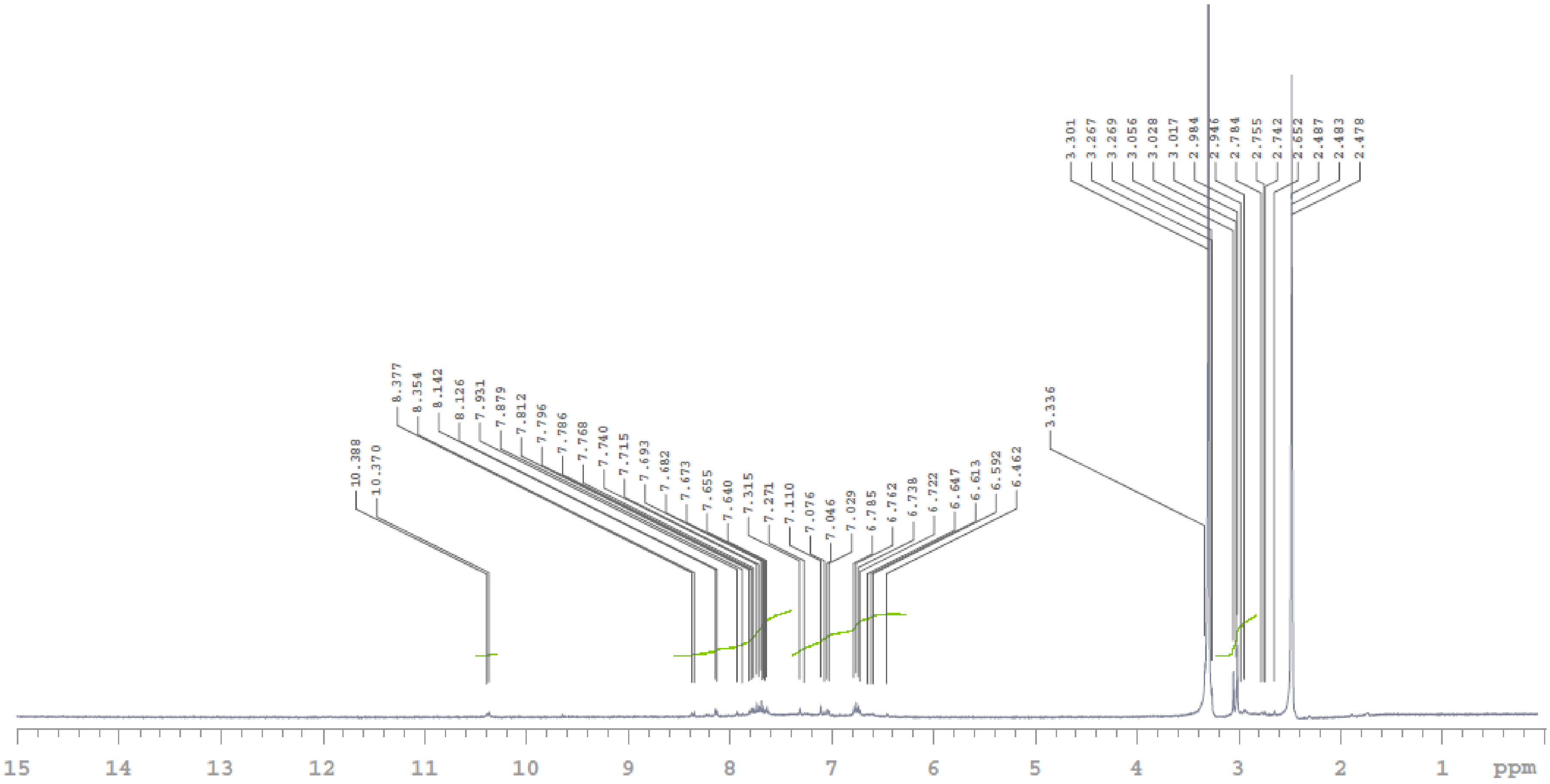

^{1}H-NMR spectrum of compound 3c

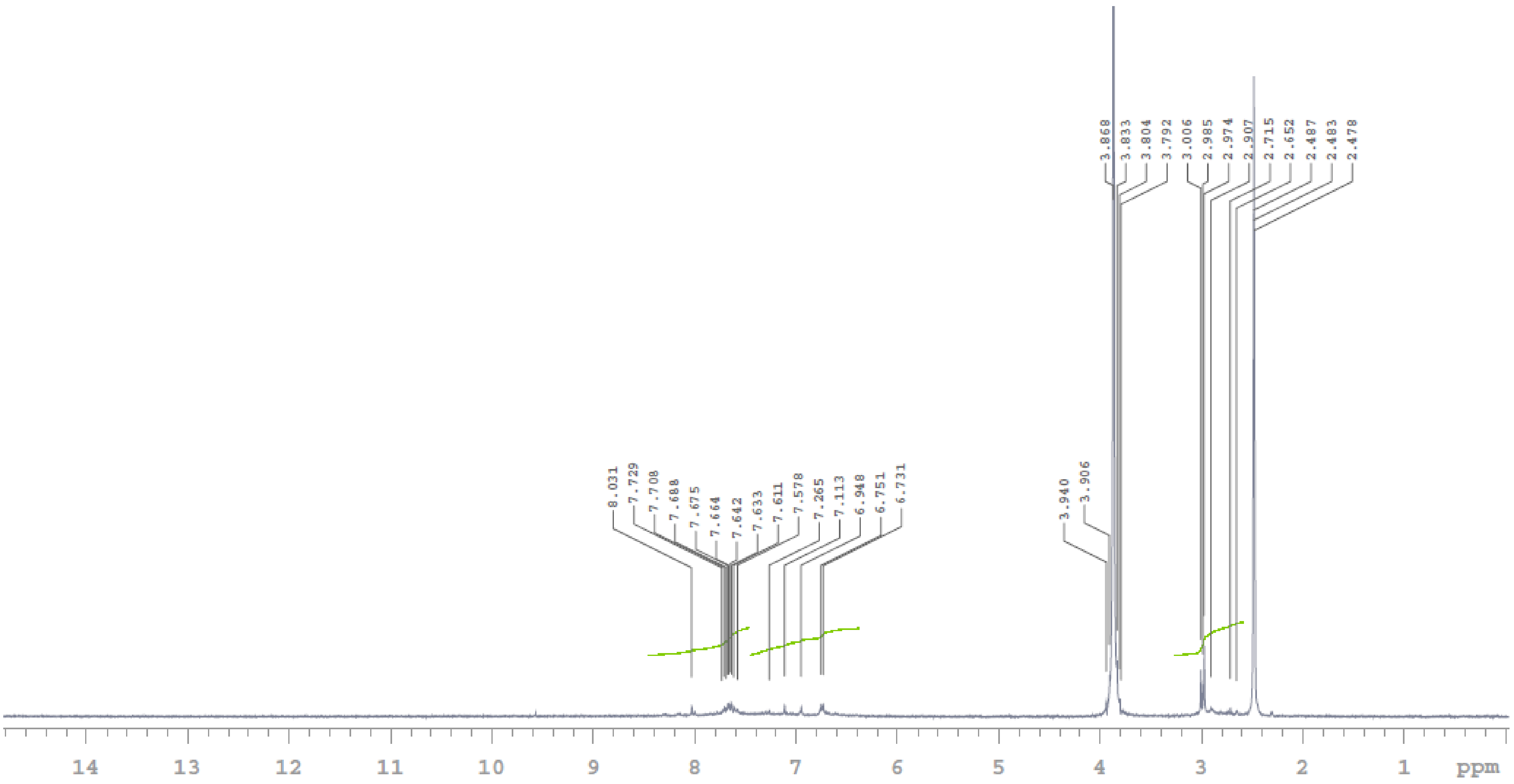

^{1}H-NMR spectrum of compound 3c- D_2O

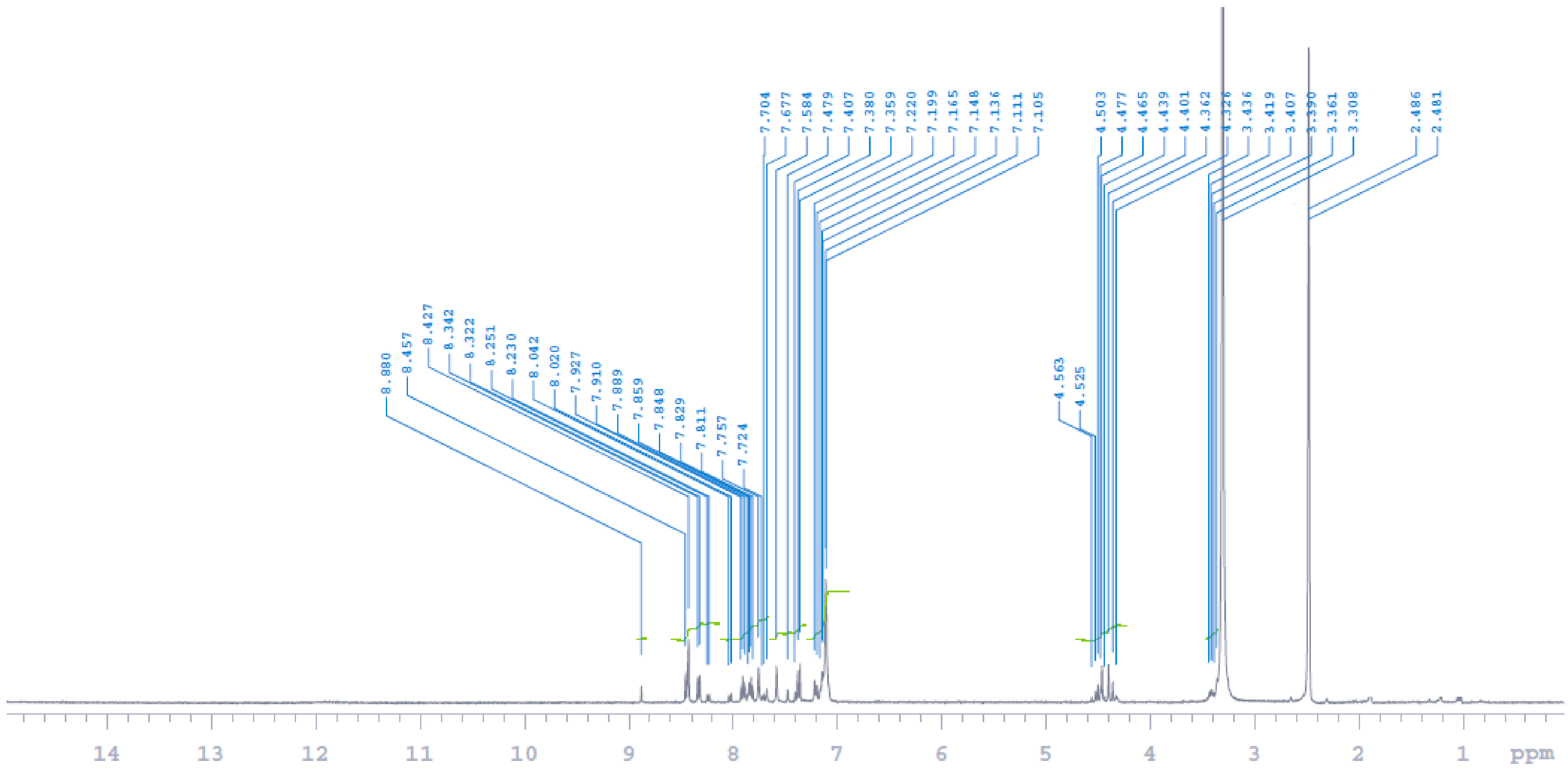

^{1}H-NMR spectrum of compound 4a

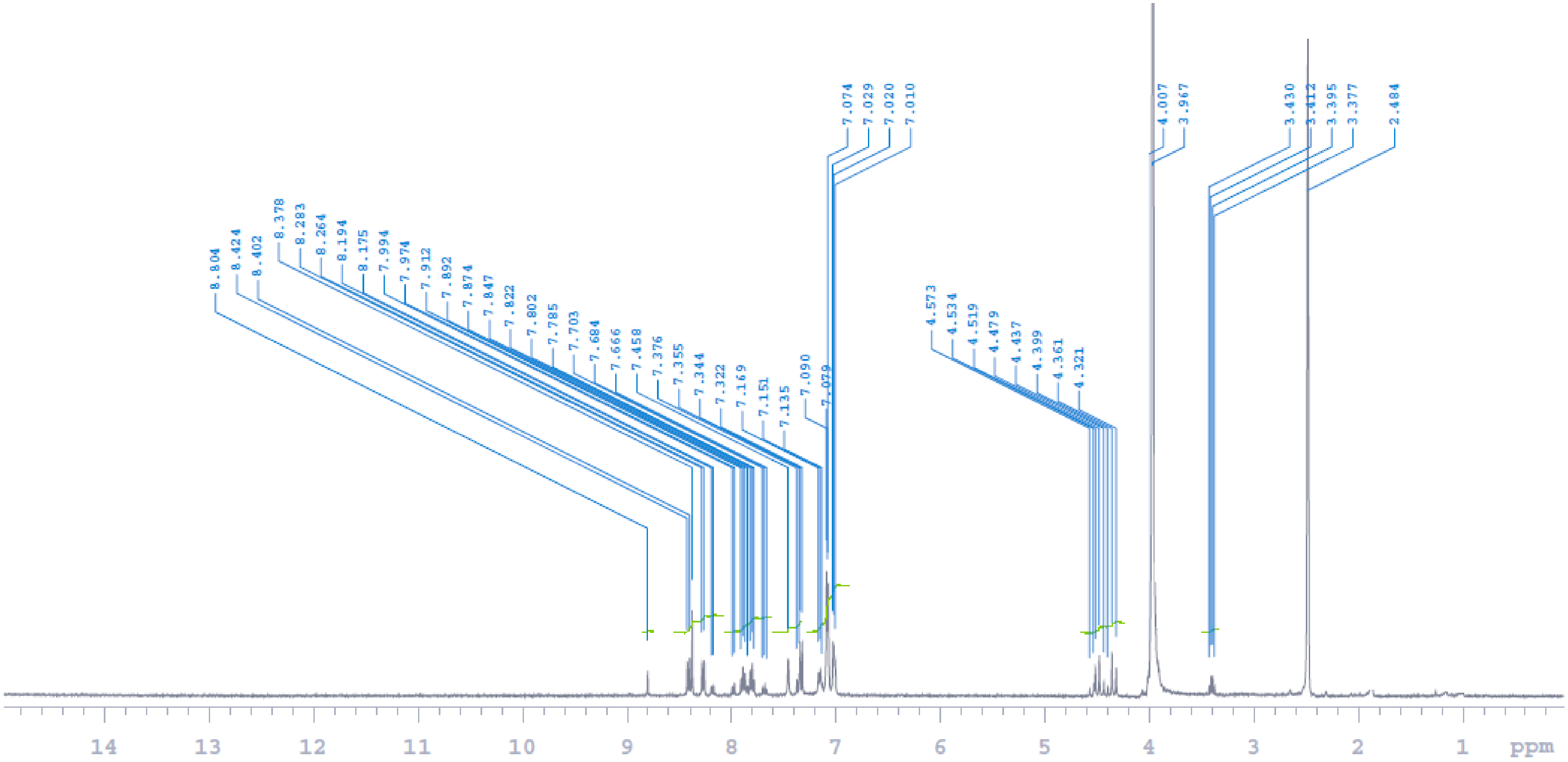

^{1}H-NMR spectrum of compound 4a- D_2O

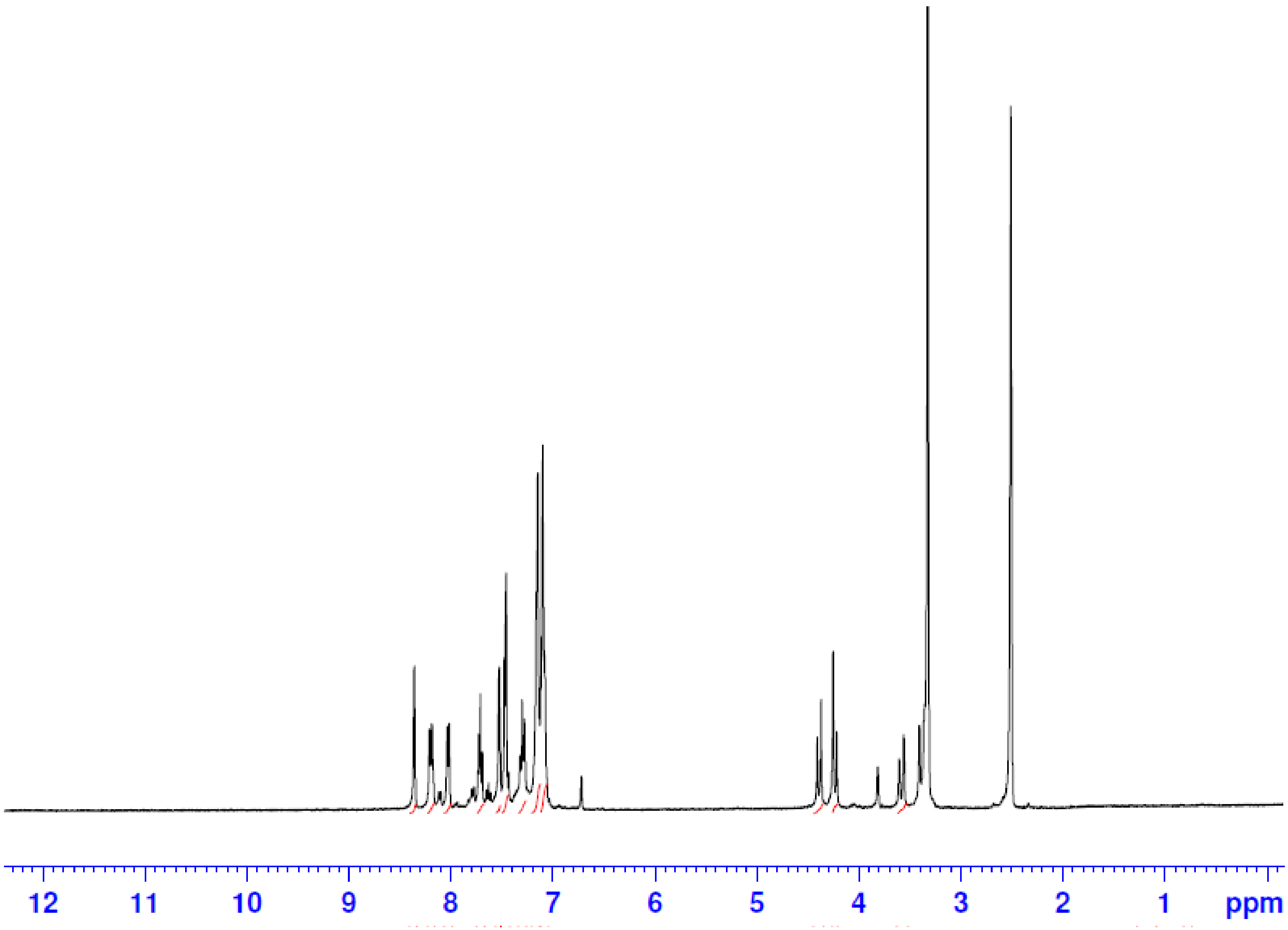

^{1}H-NMR spectrum of compound 4b

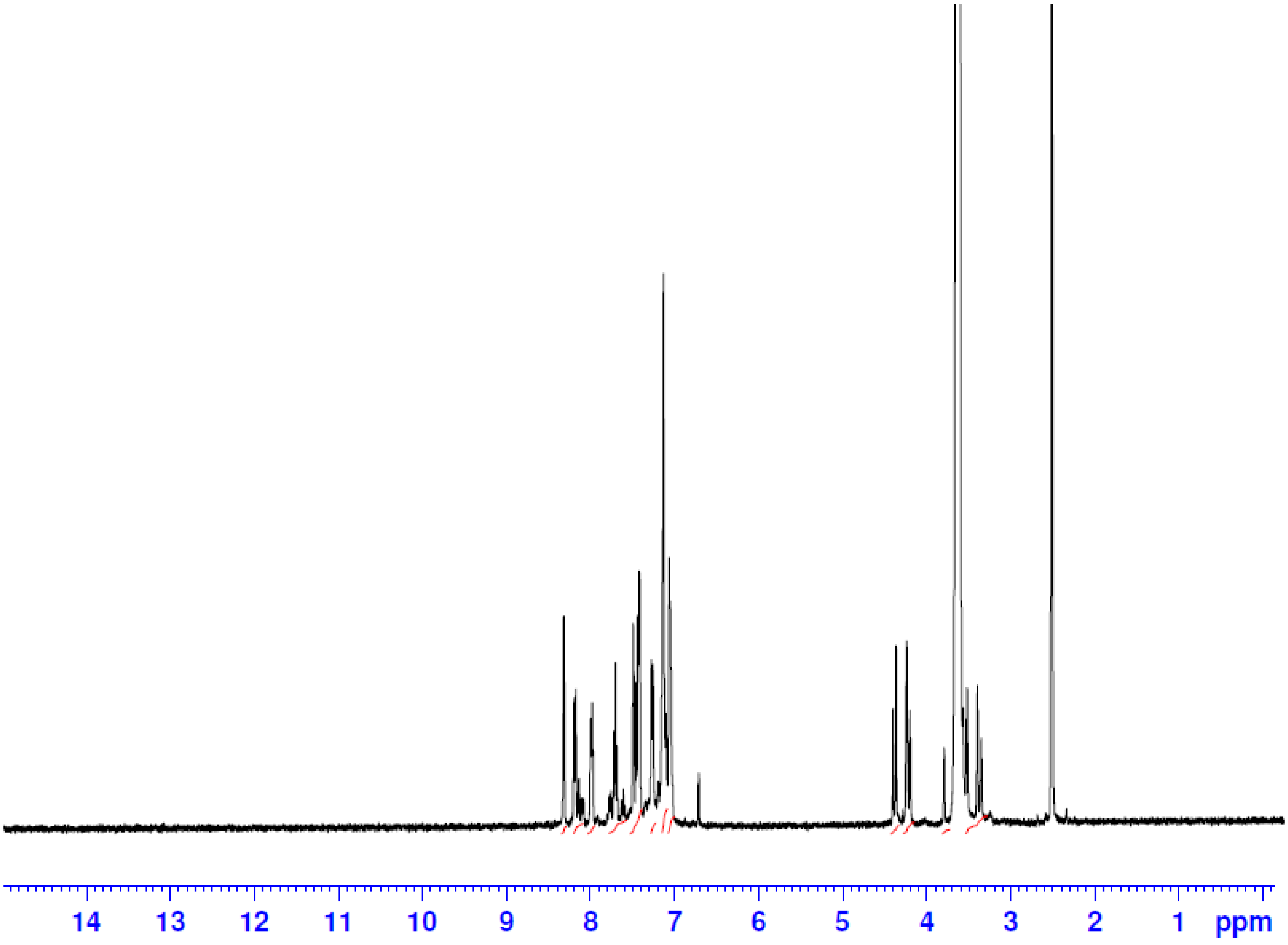

^{1}H-NMR spectrum of compound 4b- D_2O

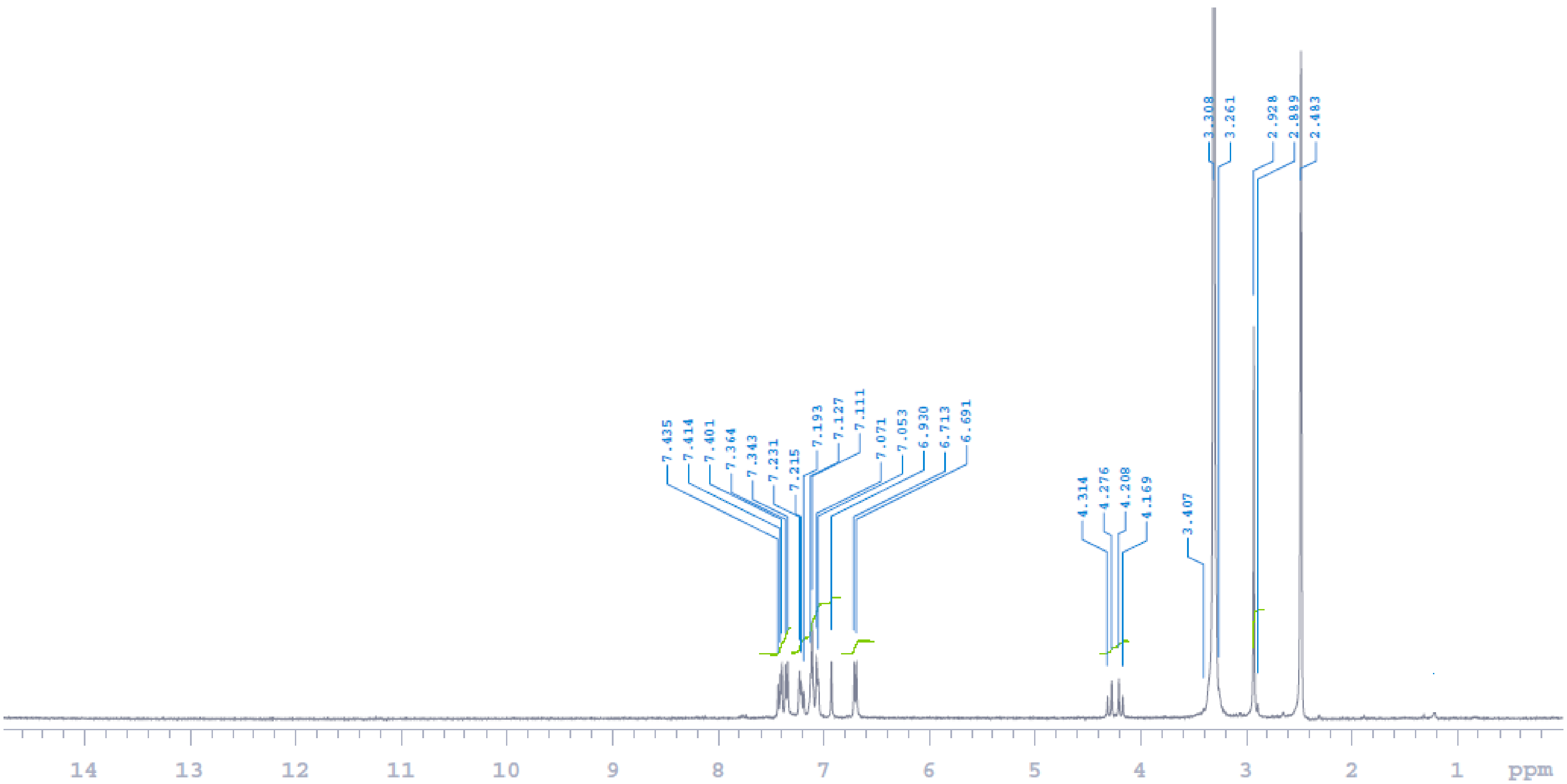

^{1}H-NMR spectrum of compound 4c

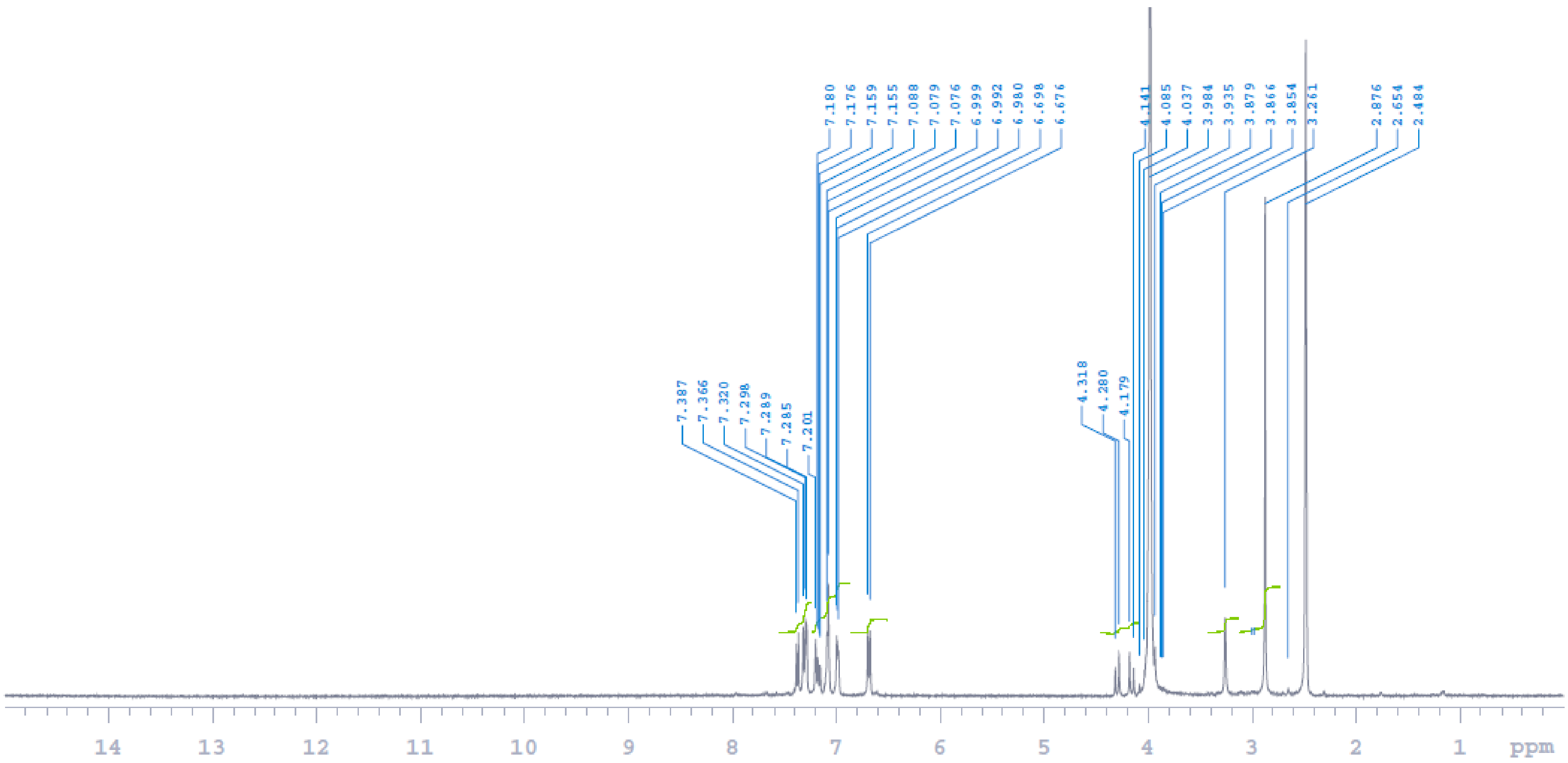

^{1}H-NMR spectrum of compound 4c- D_2O

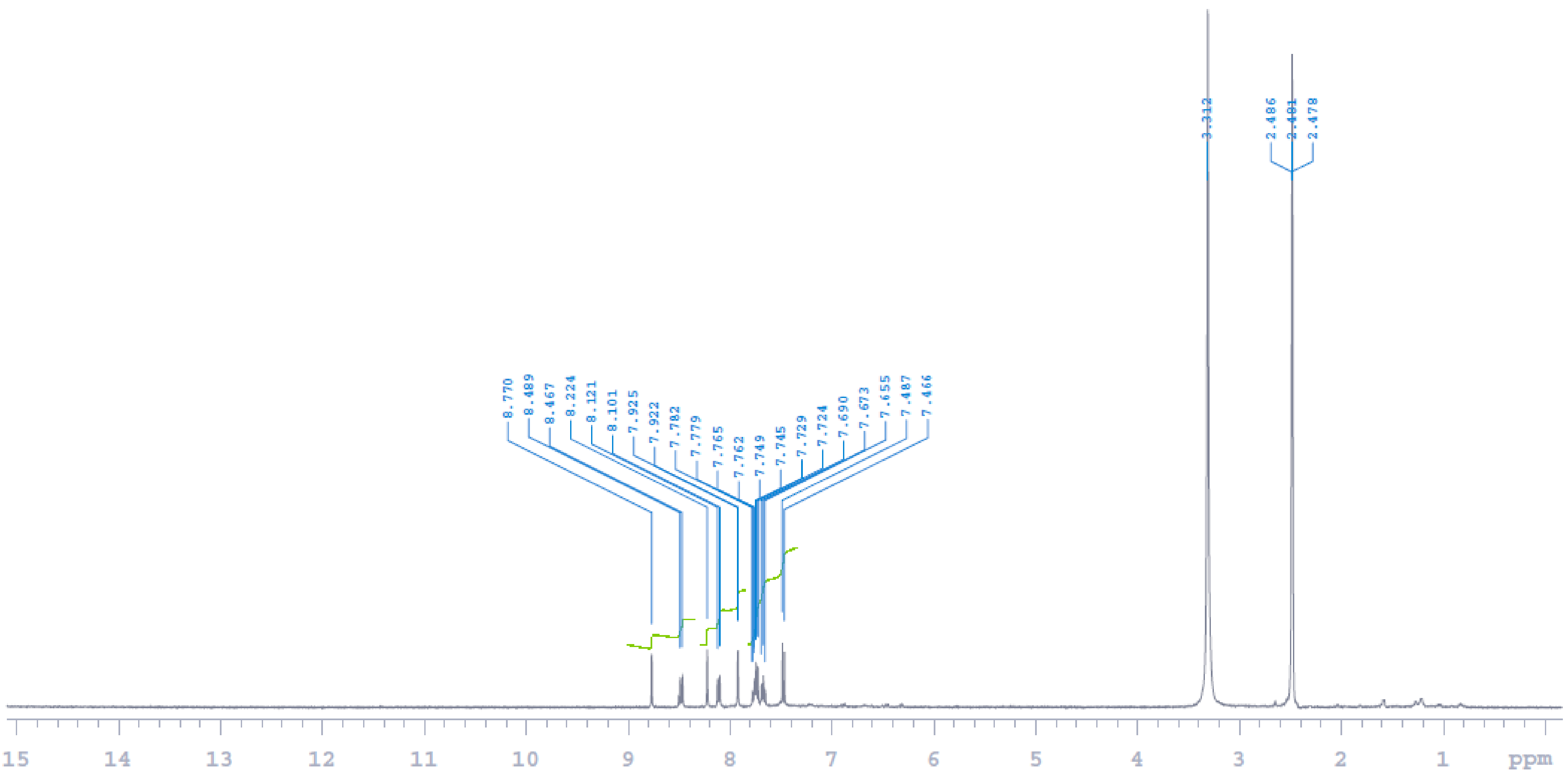

^{1}H-NMR spectrum of compound 5a

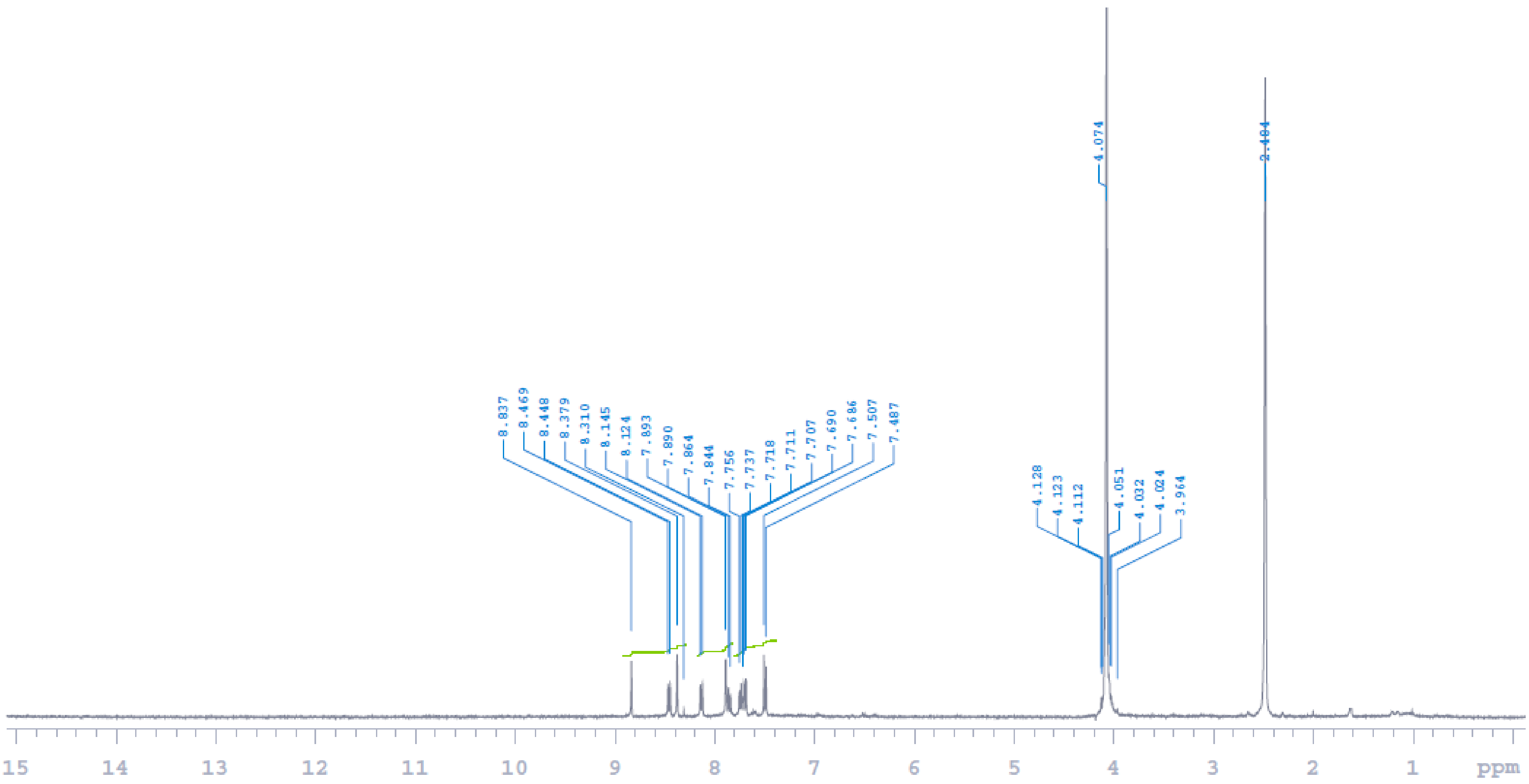

^{1}H-NMR spectrum of compound 5a- D_2O

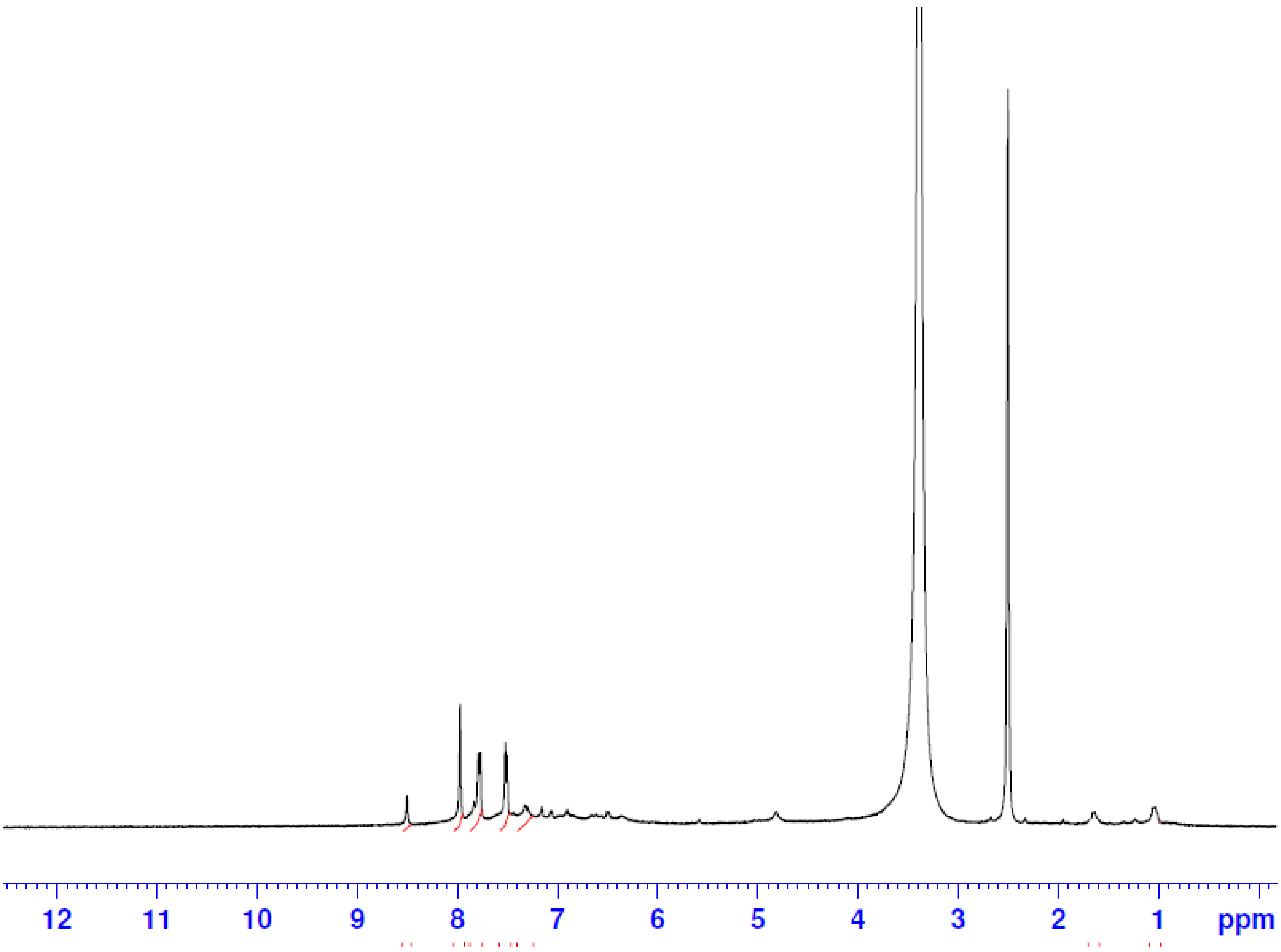

^{1}H-NMR spectrum of compound 5b

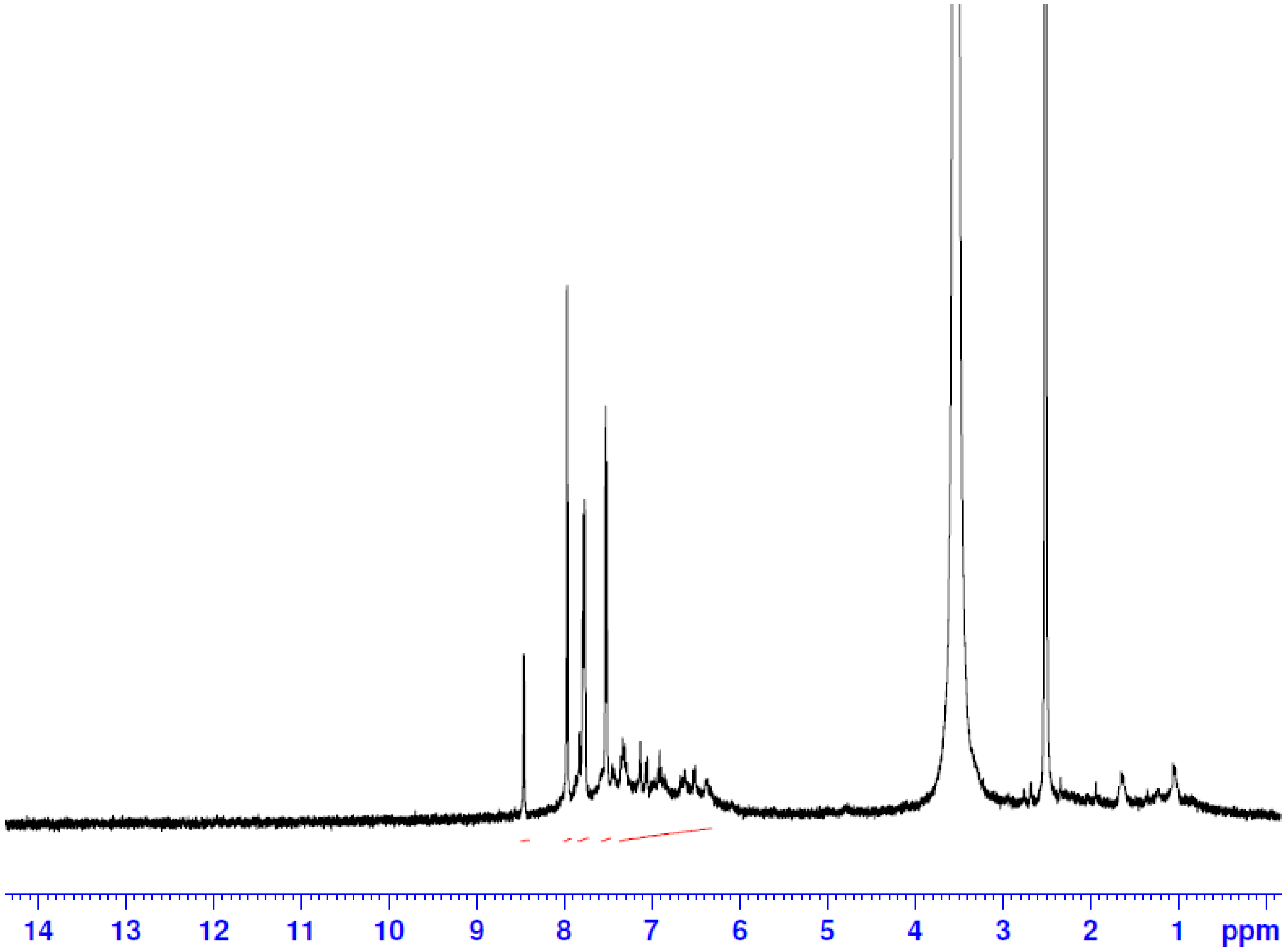

^{1}H-NMR spectrum of compound 5b- D_2O

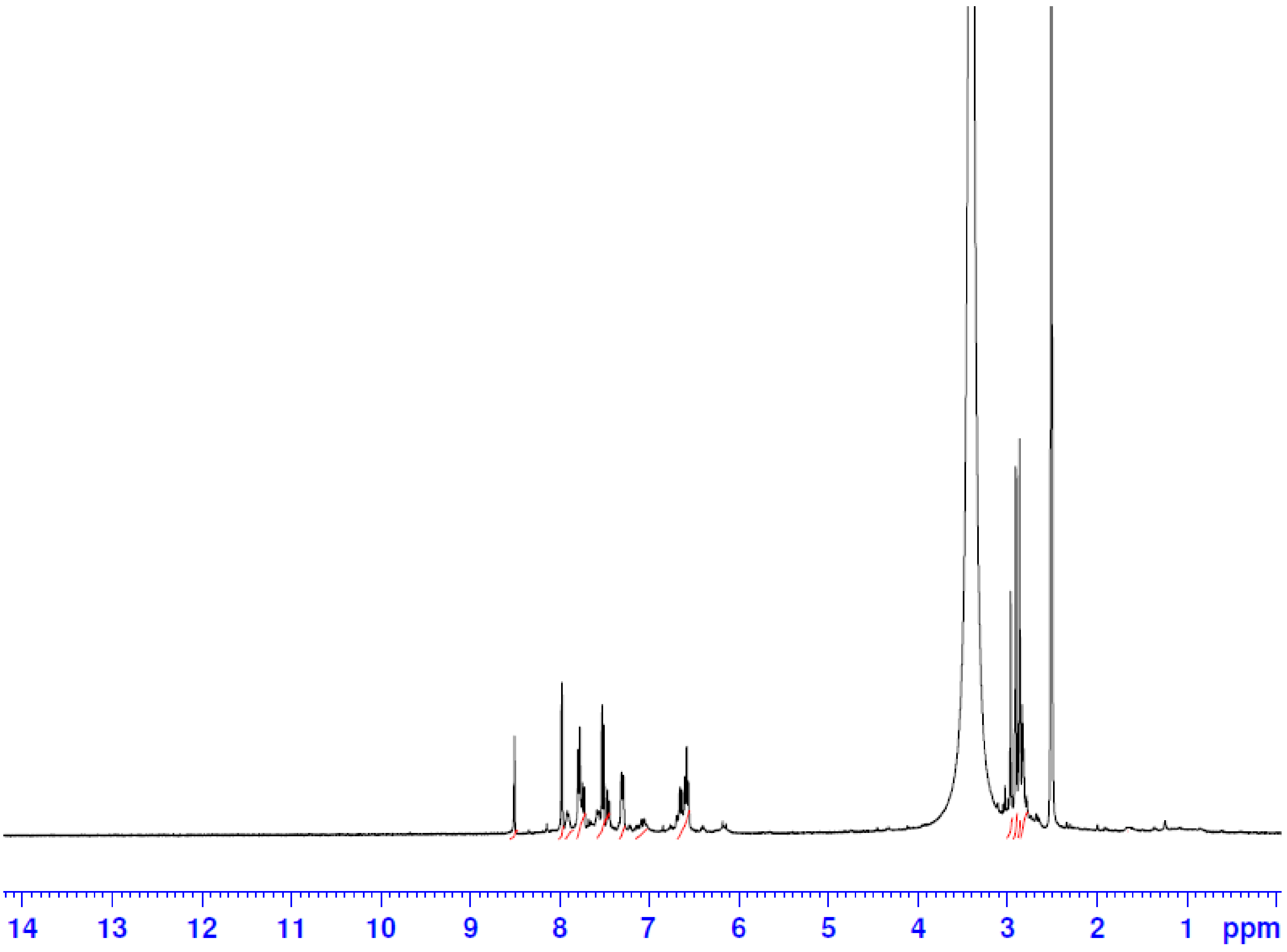

^{1}H-NMR spectrum of compound 5c

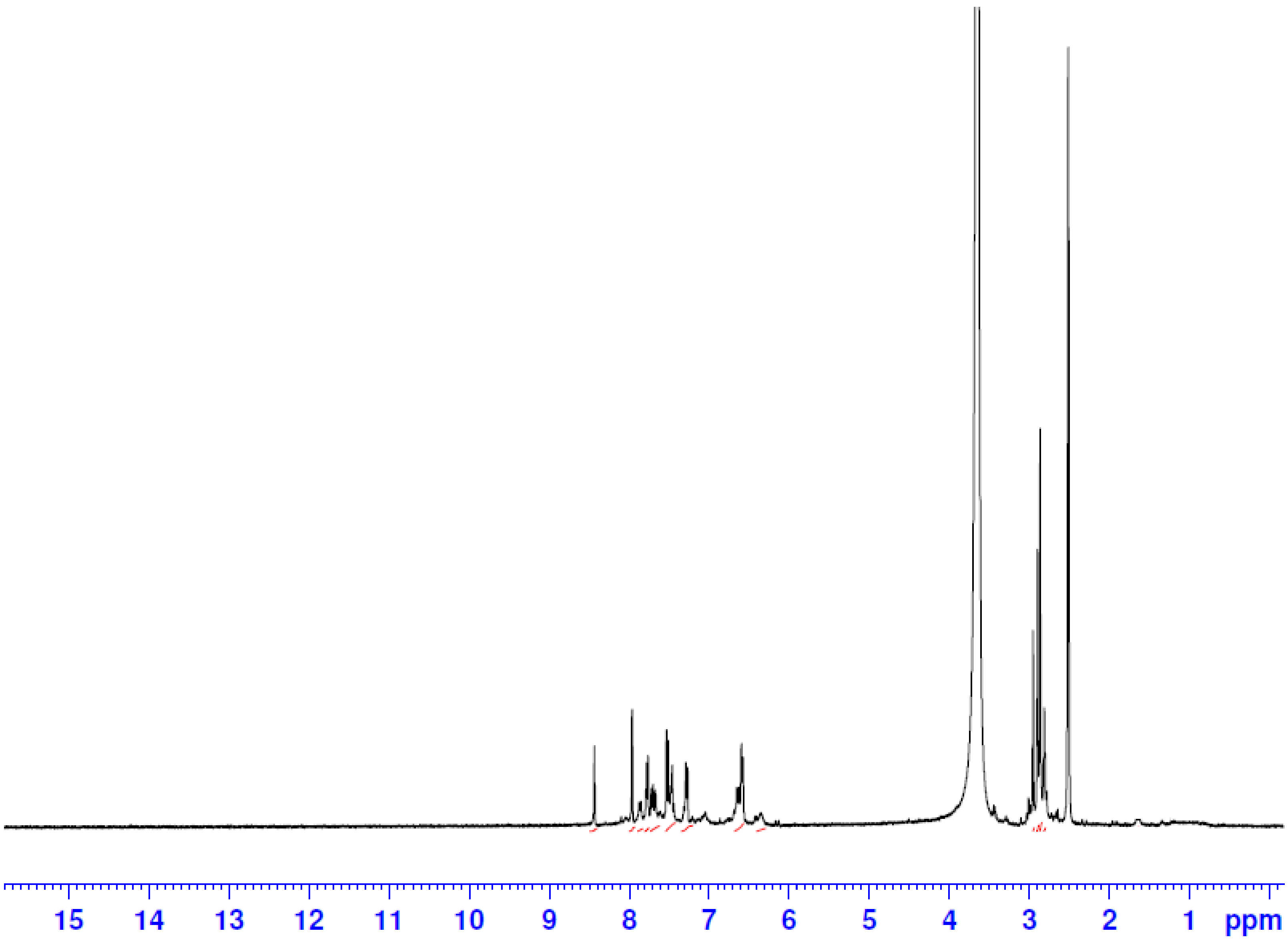

^{1}H-NMR spectrum of compound 5c- D_2O

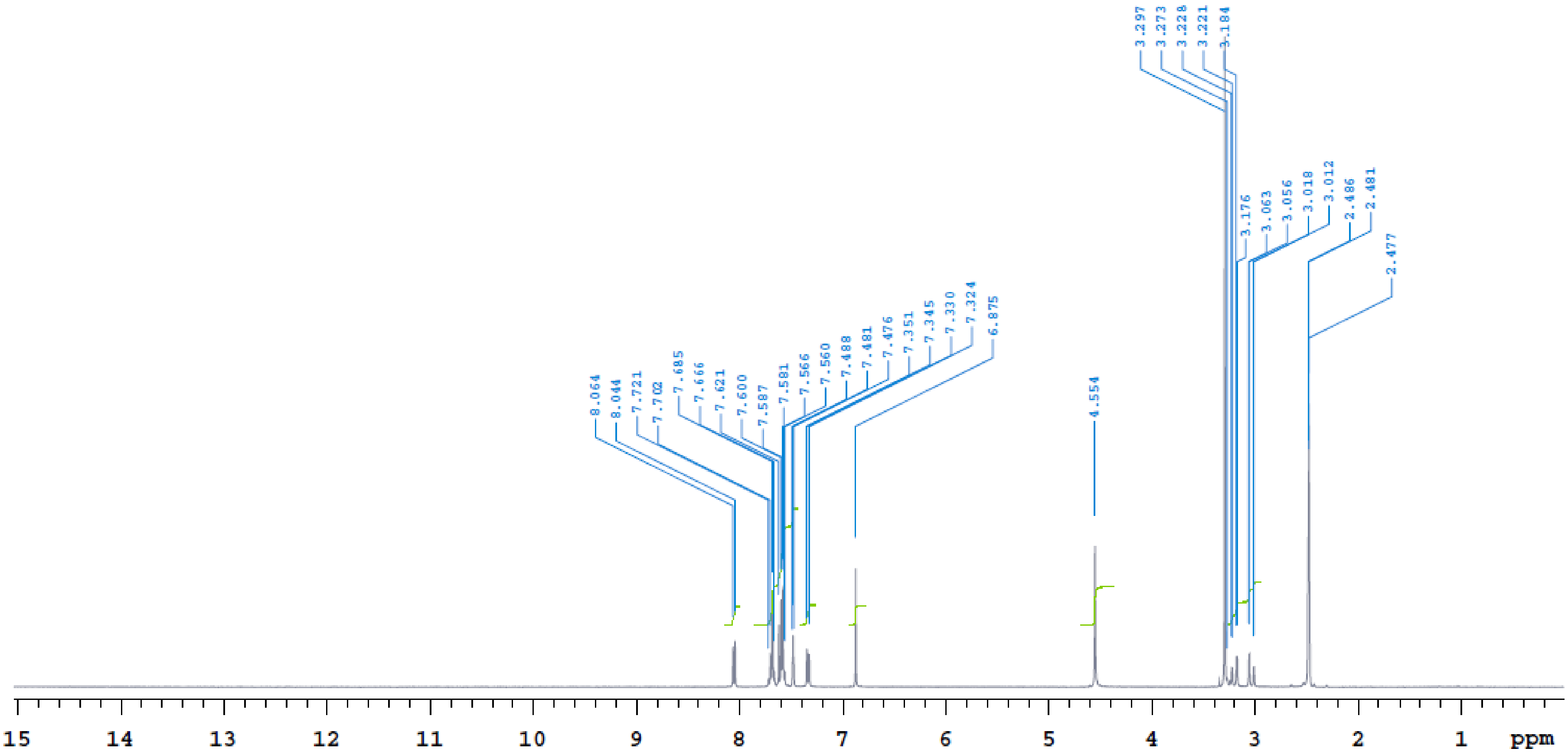

^{1}H-NMR spectrum of compound 6a

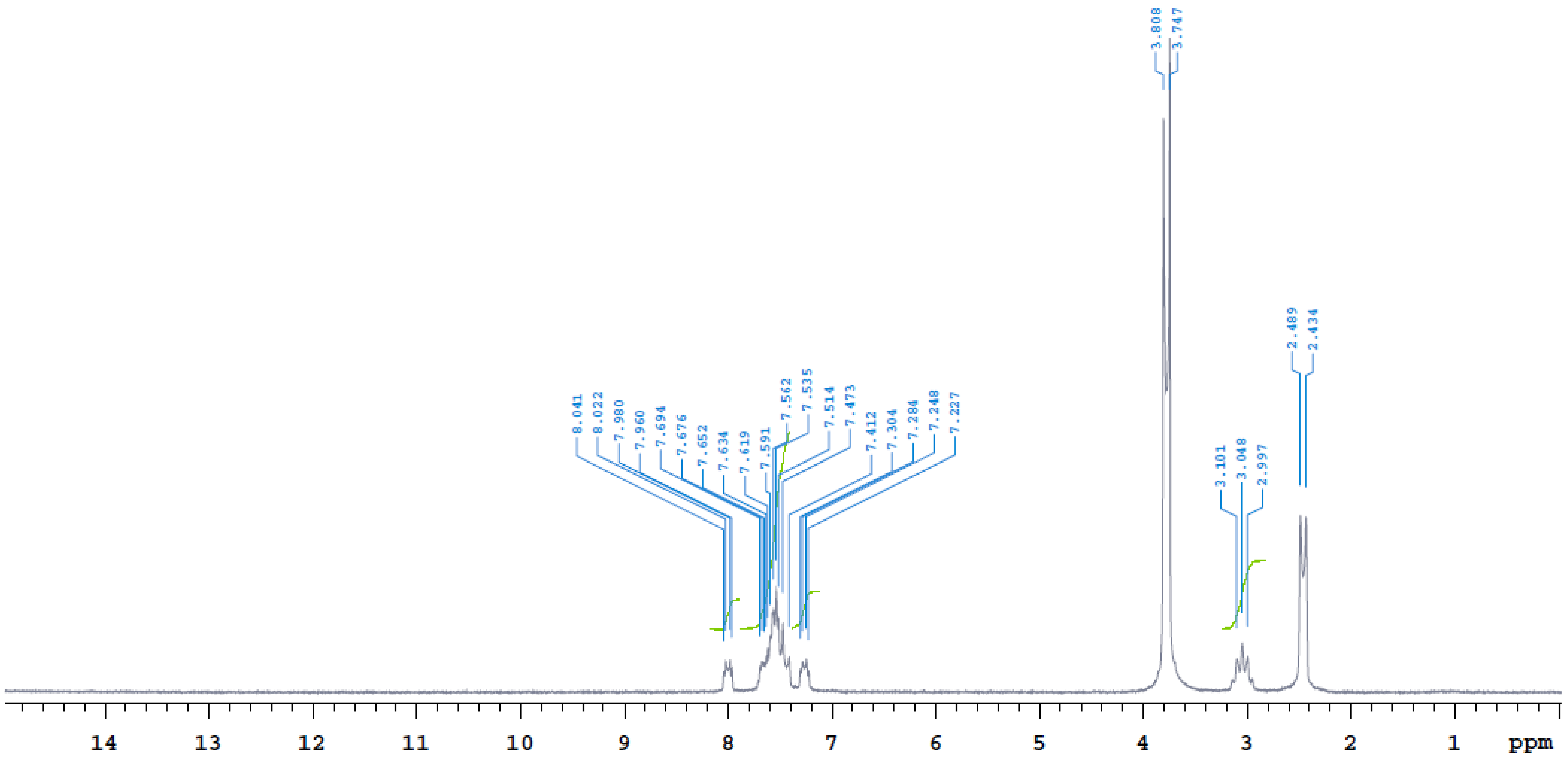

^{1}H-NMR spectrum of compound 6a- D_2O

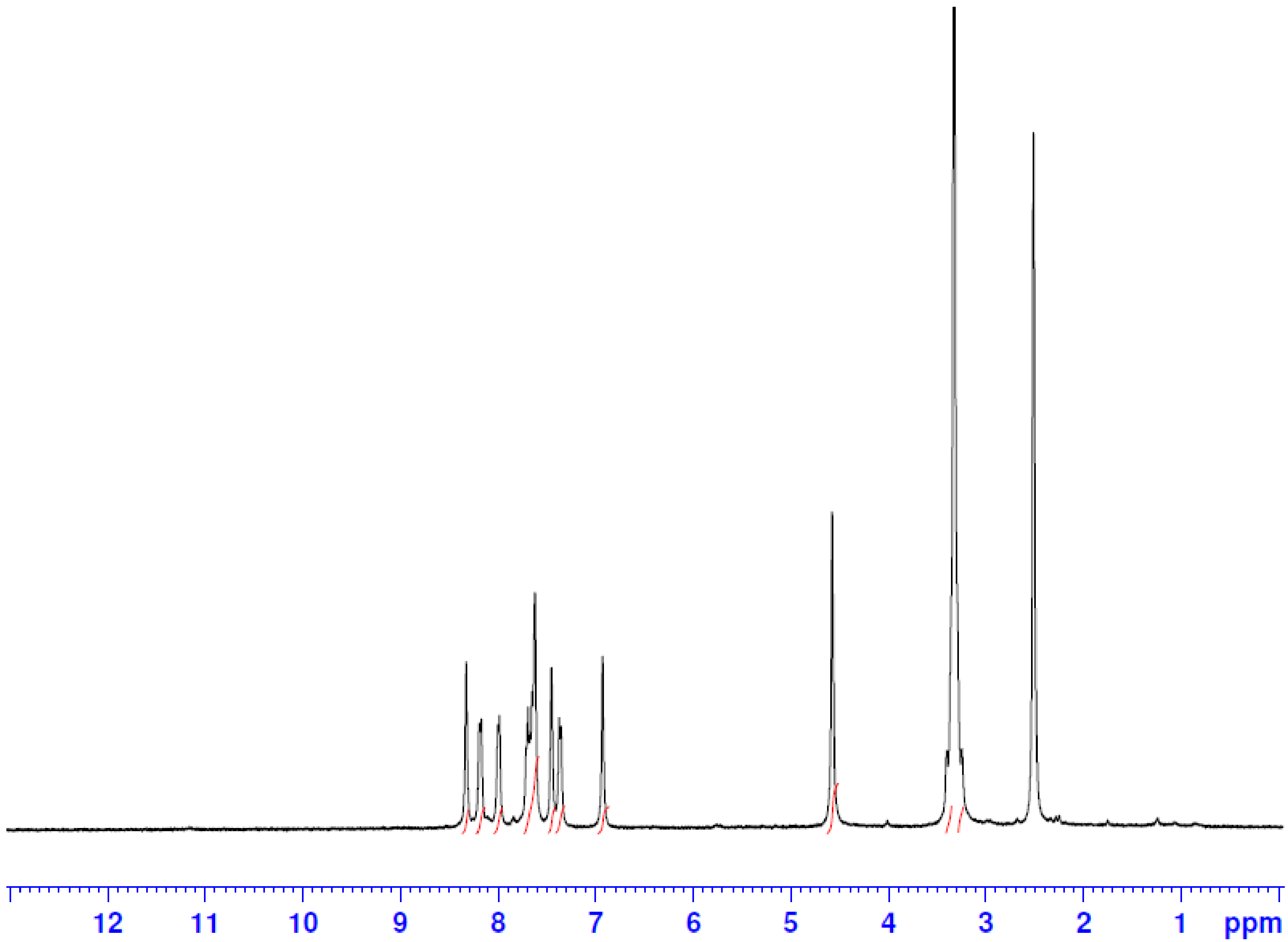

^{1}H-NMR spectrum of compound 6b

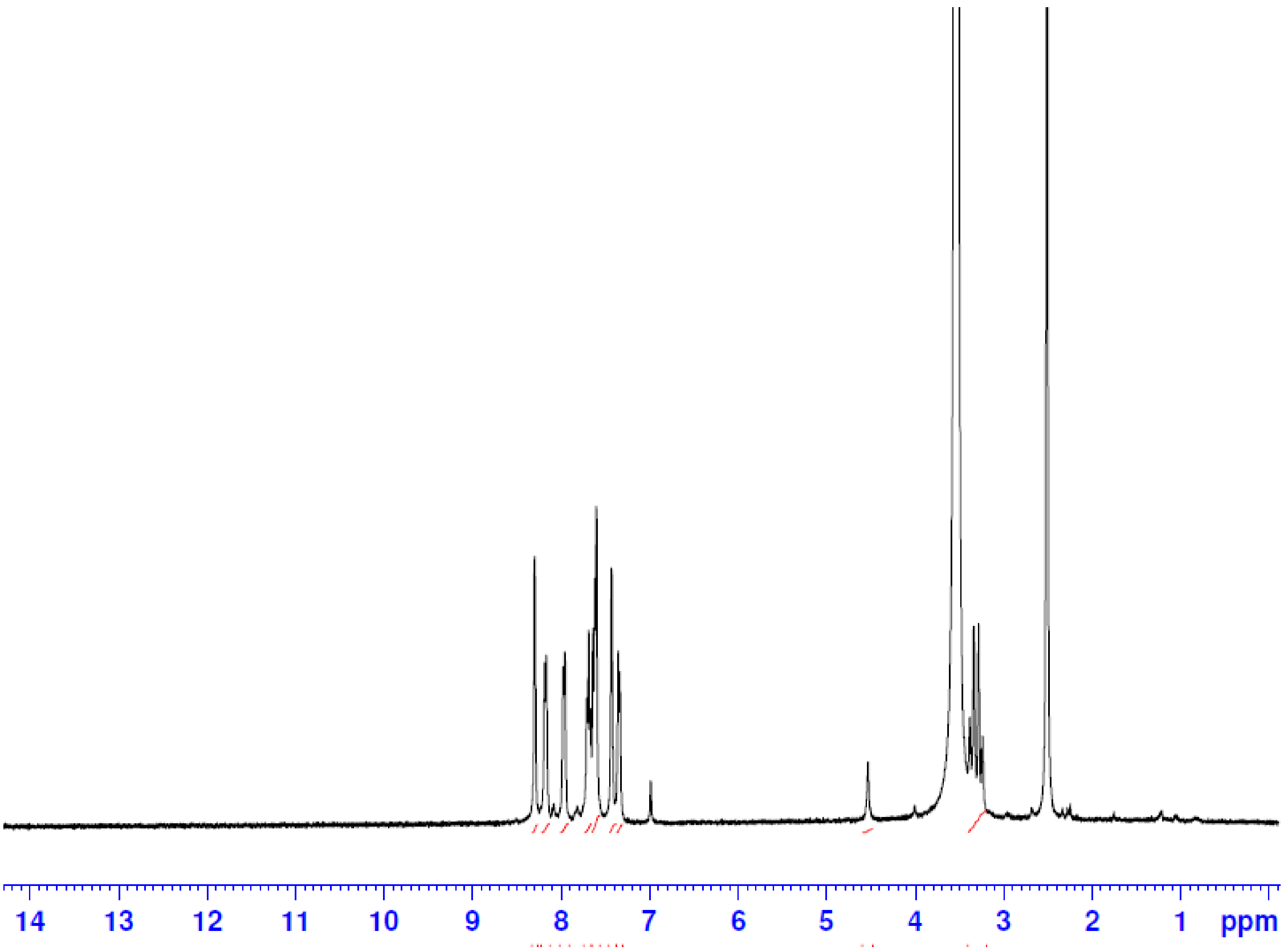

^{1}H-NMR spectrum of compound 6b- D_2O

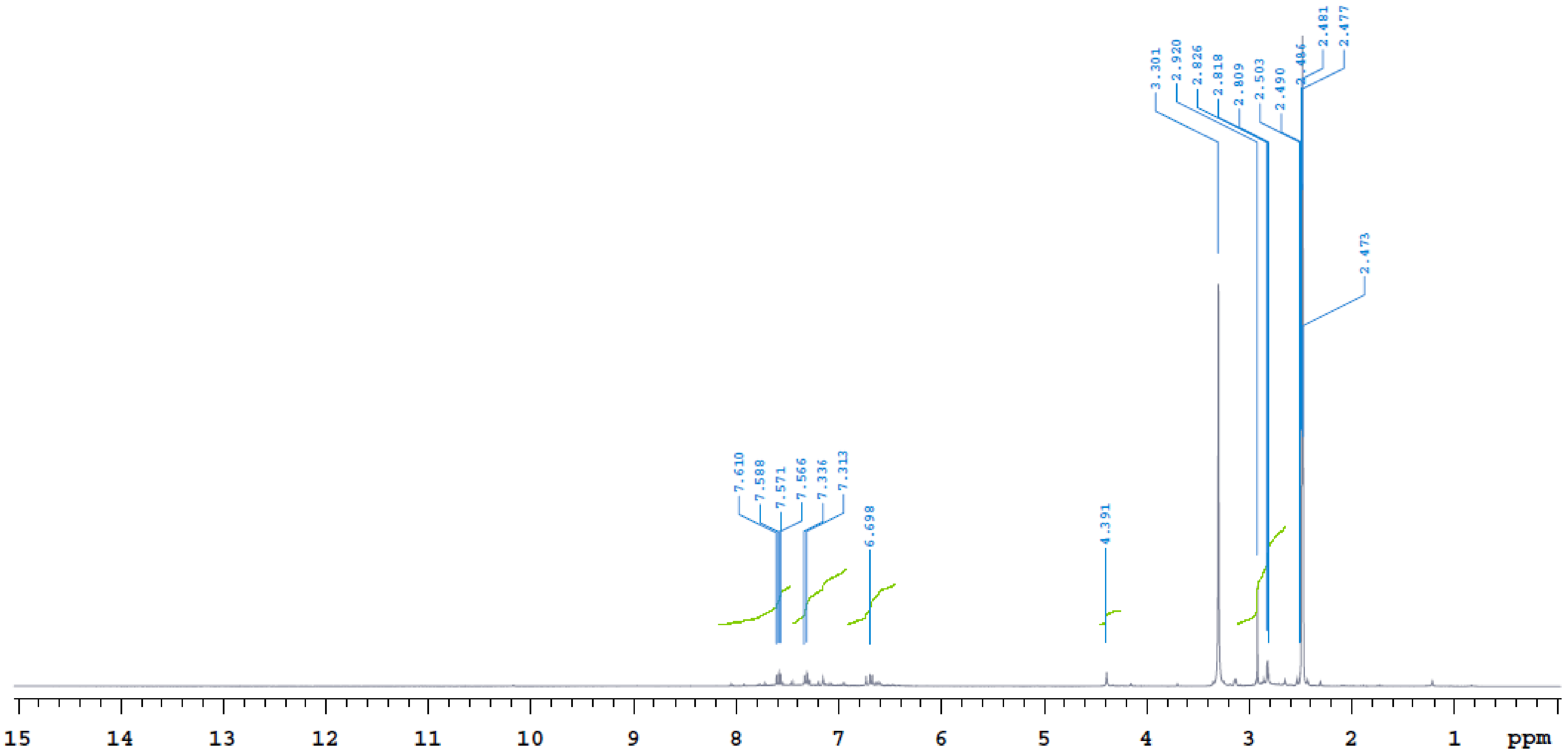

^{1}H-NMR spectrum of compound 6c

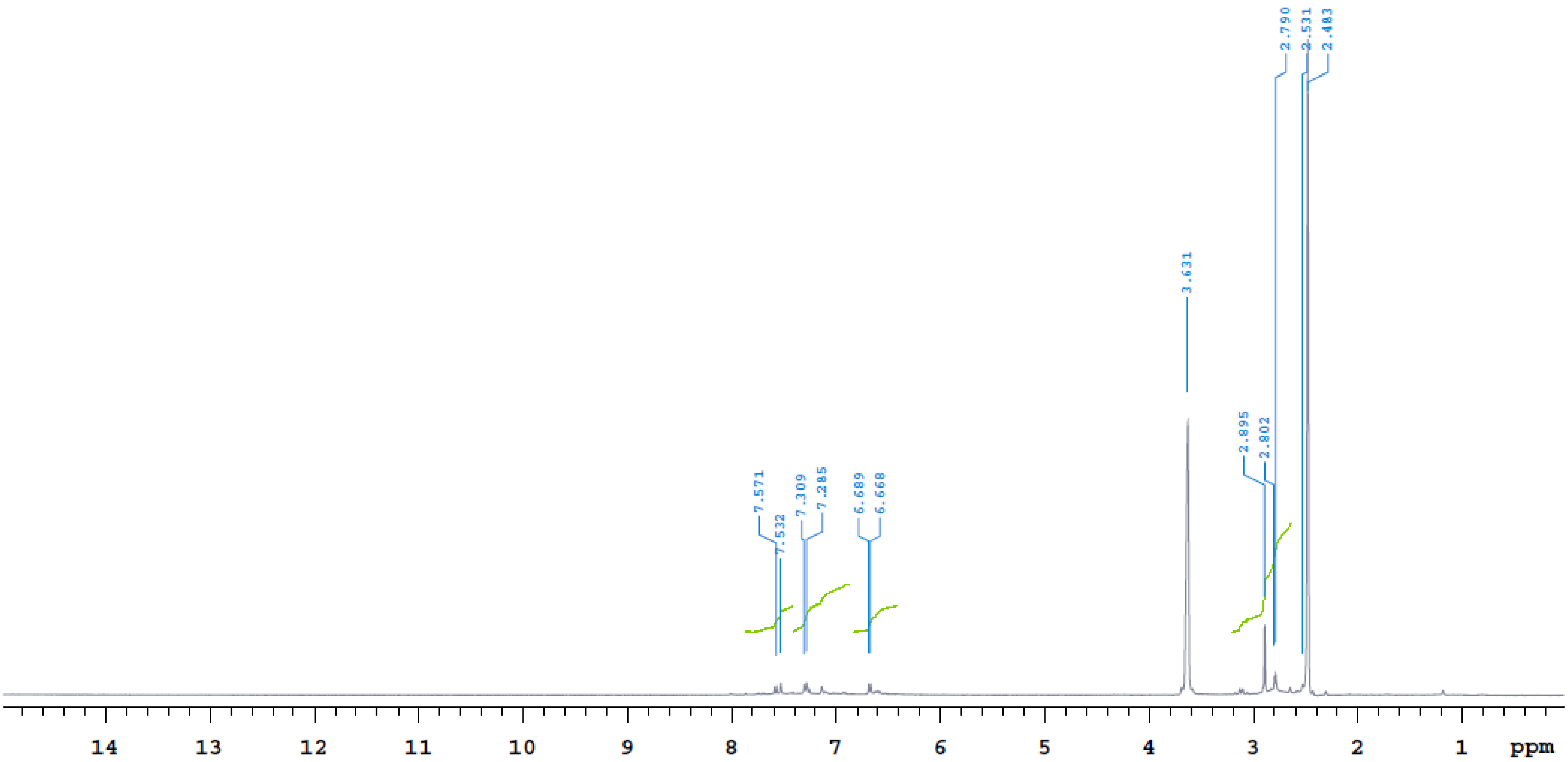

^{1}H-NMR spectrum of compound 6c- D_2O

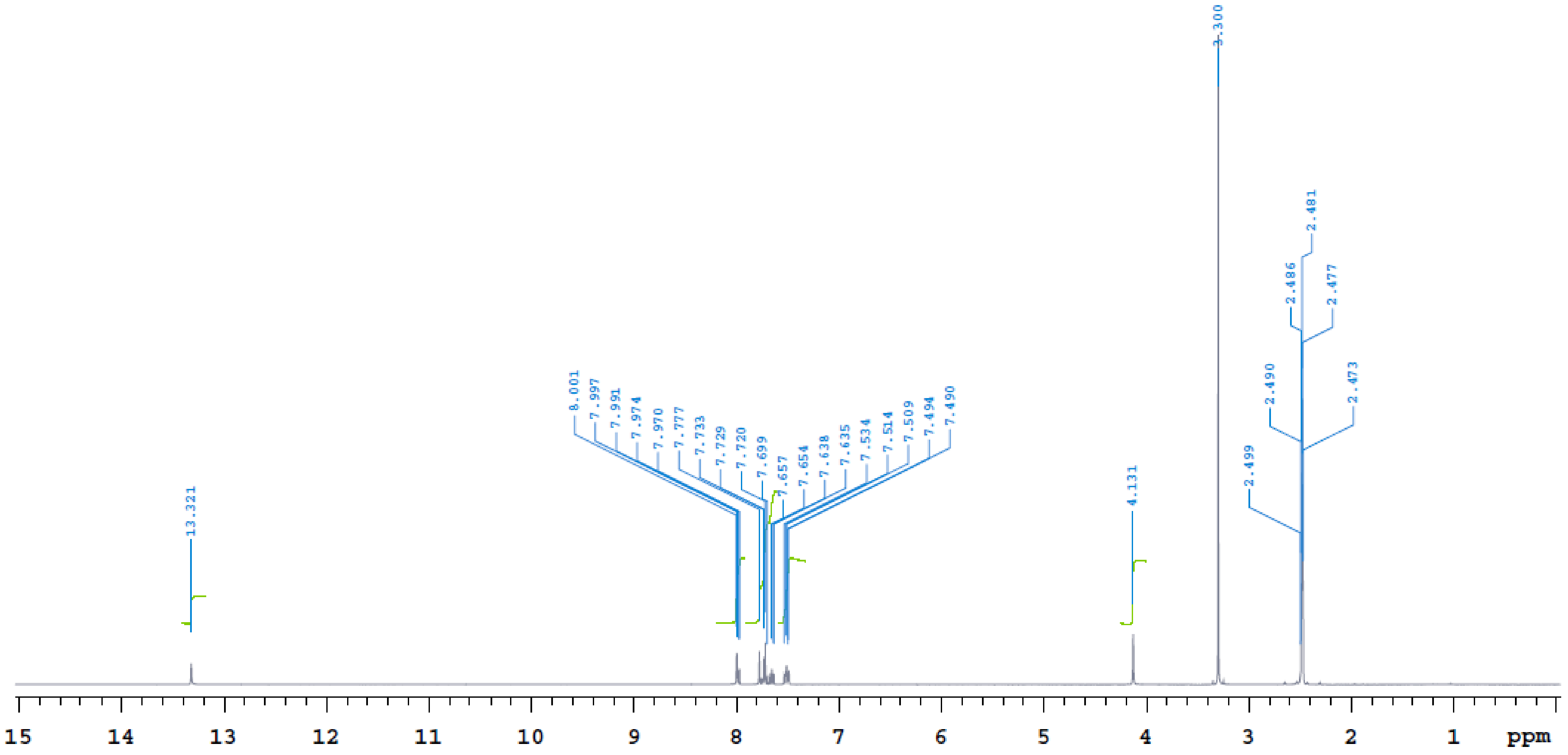

^{1}H-NMR spectrum of compound 7a

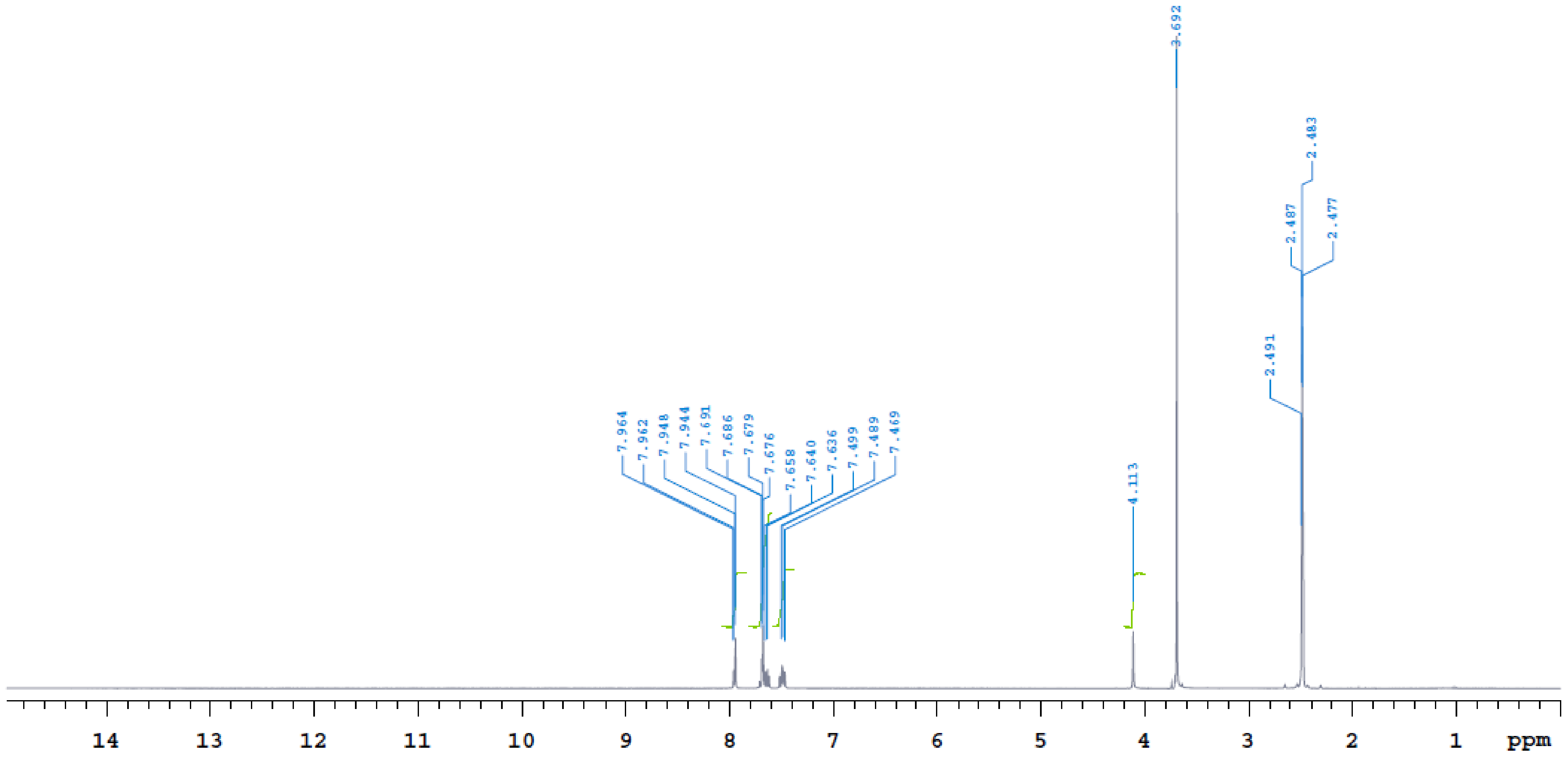

^{1}H-NMR spectrum of compound 7a D_2O

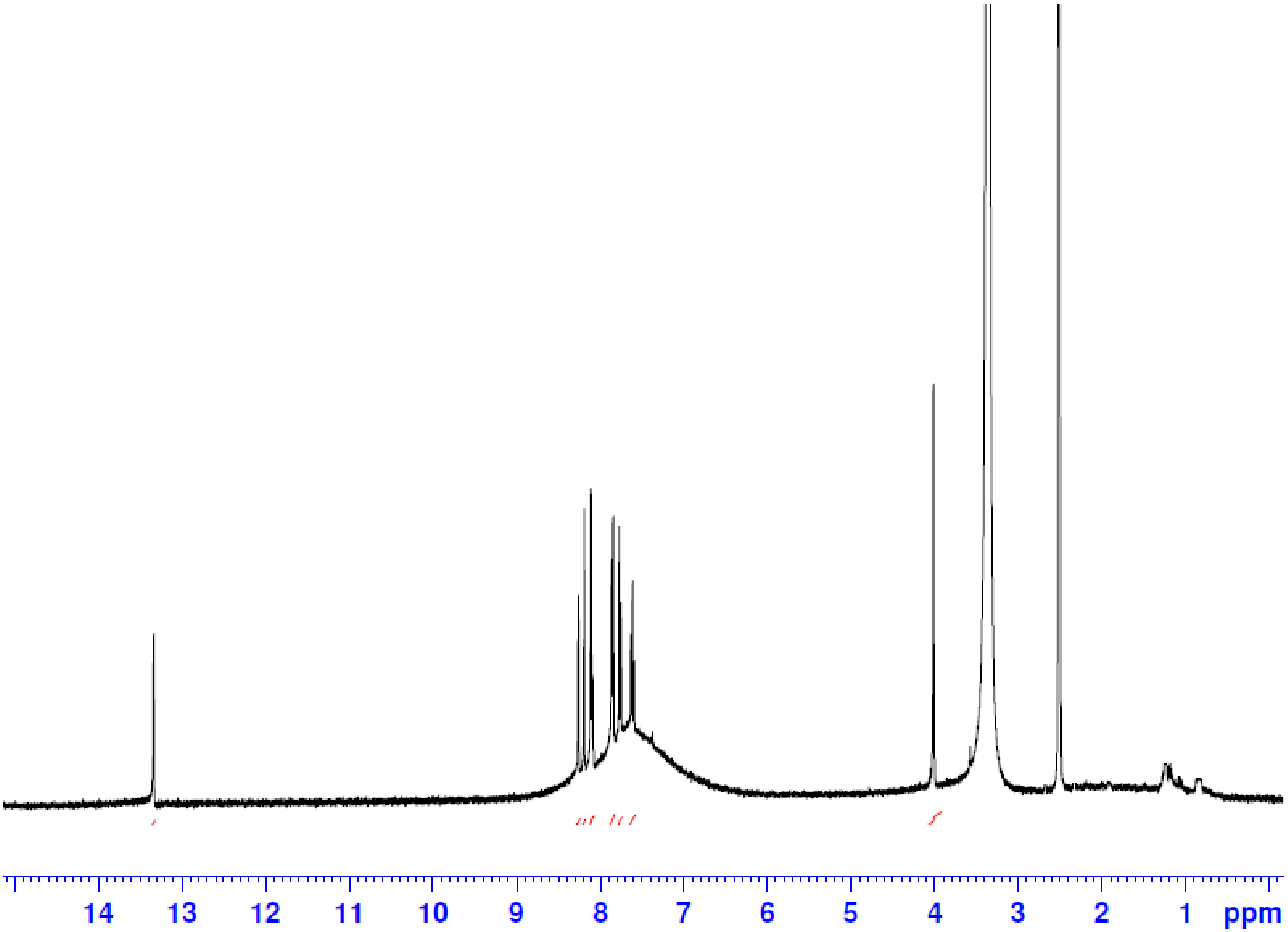

^{1}H-NMR spectrum of compound 7b

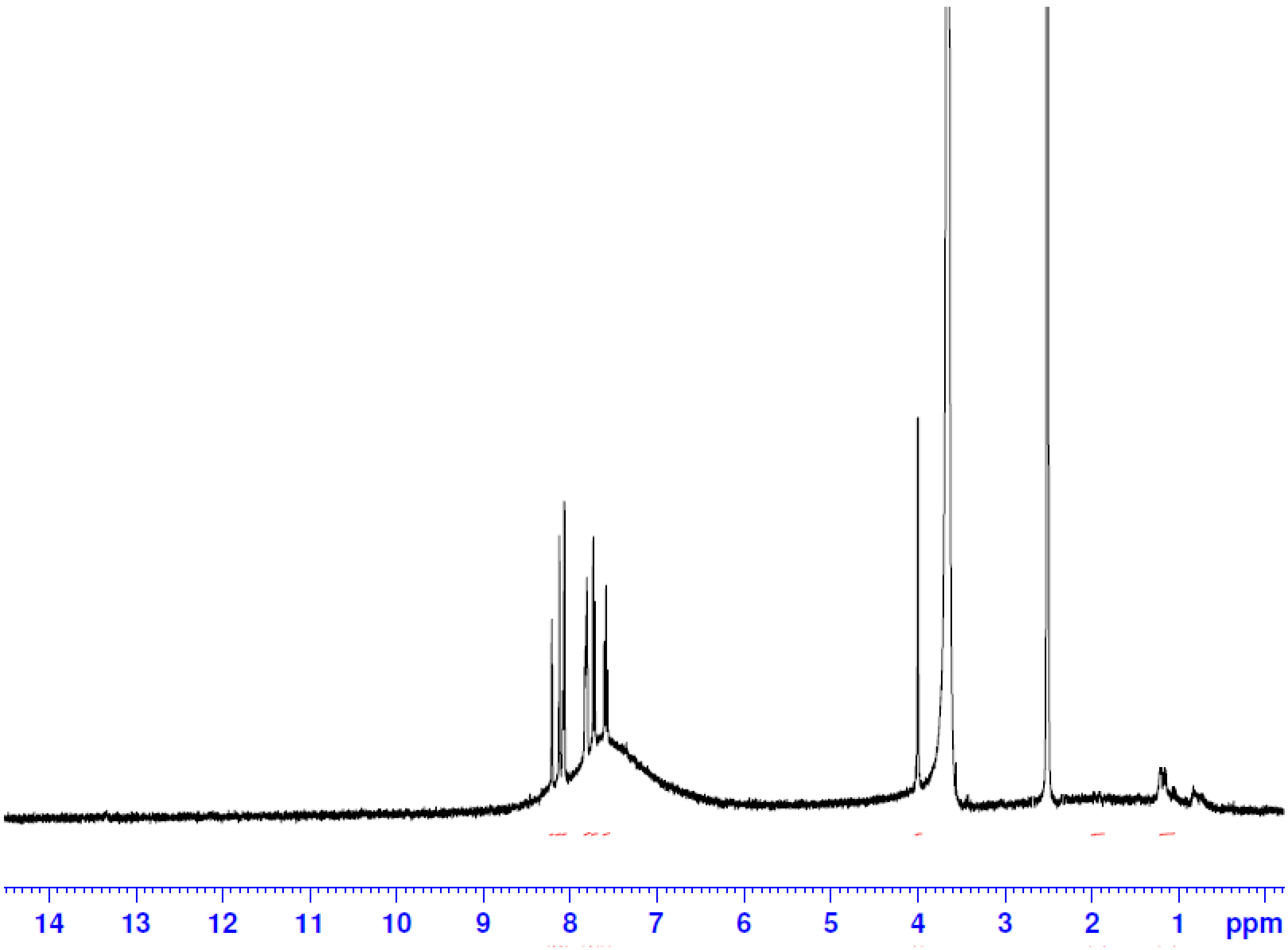

^{1}H-NMR spectrum of compound 7b D_2O

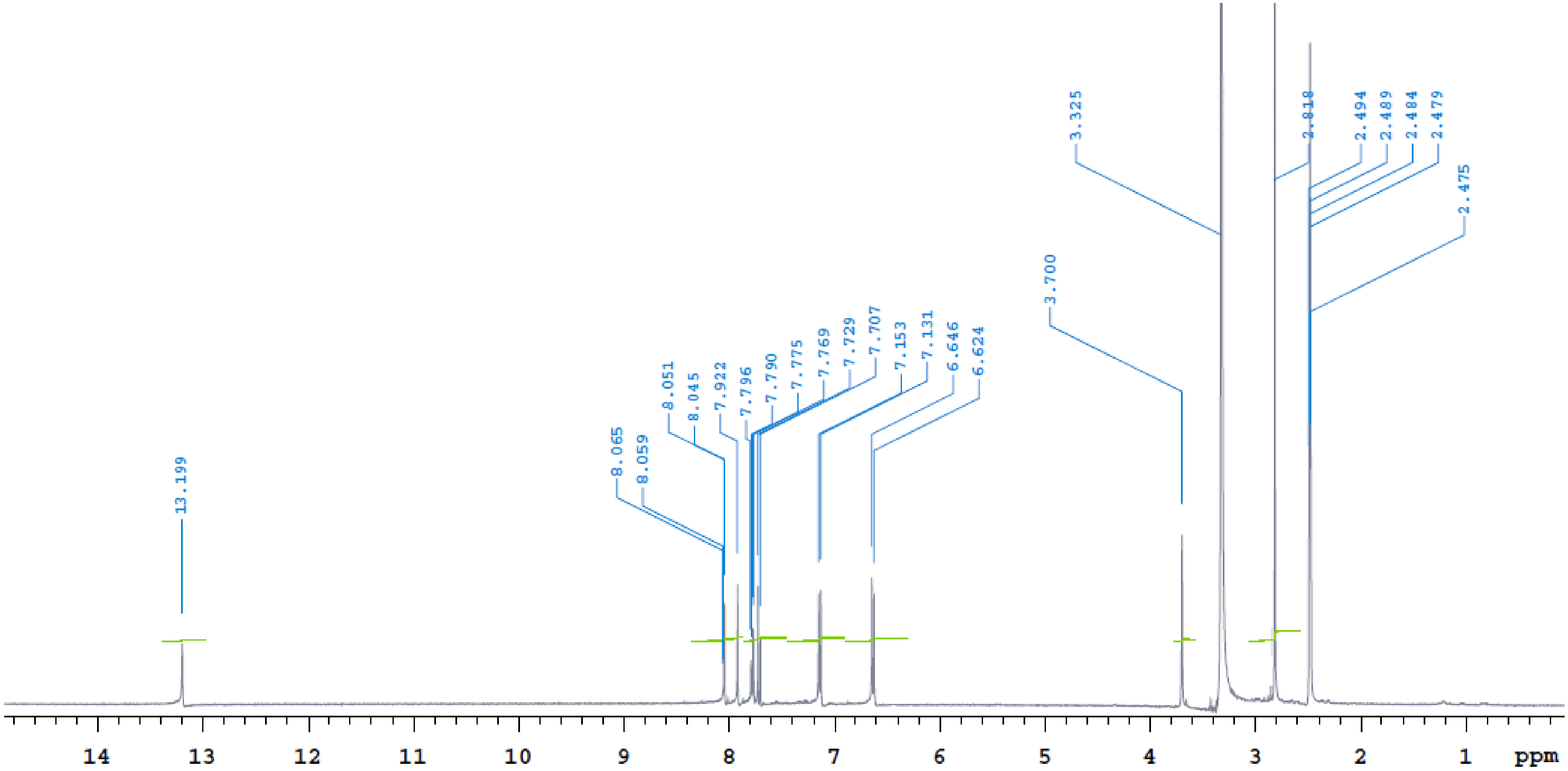

^{1}H-NMR spectrum of compound 7c

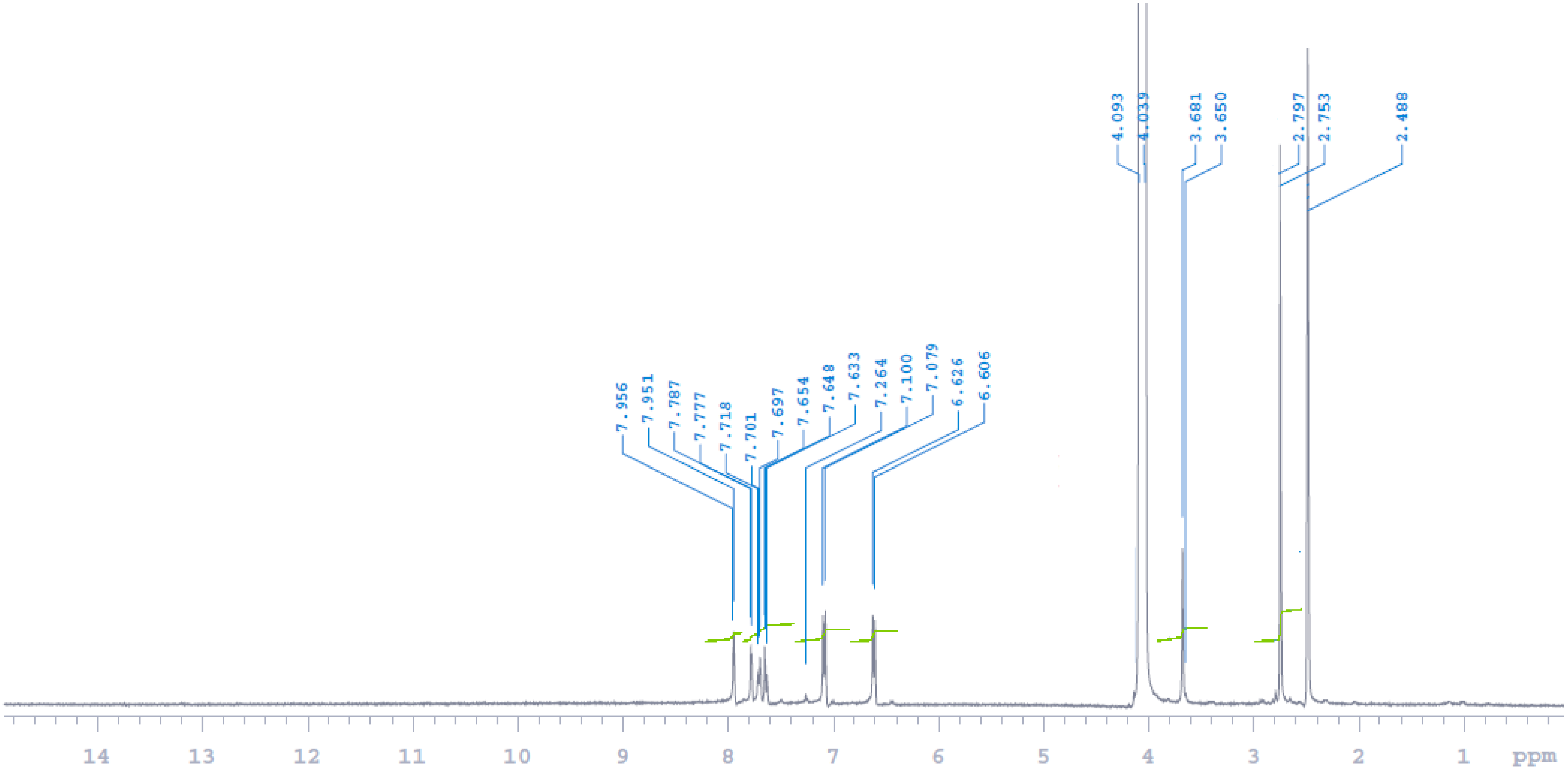

<u>^{1}H-NMR spectrum of compound 7c D_2O</u>

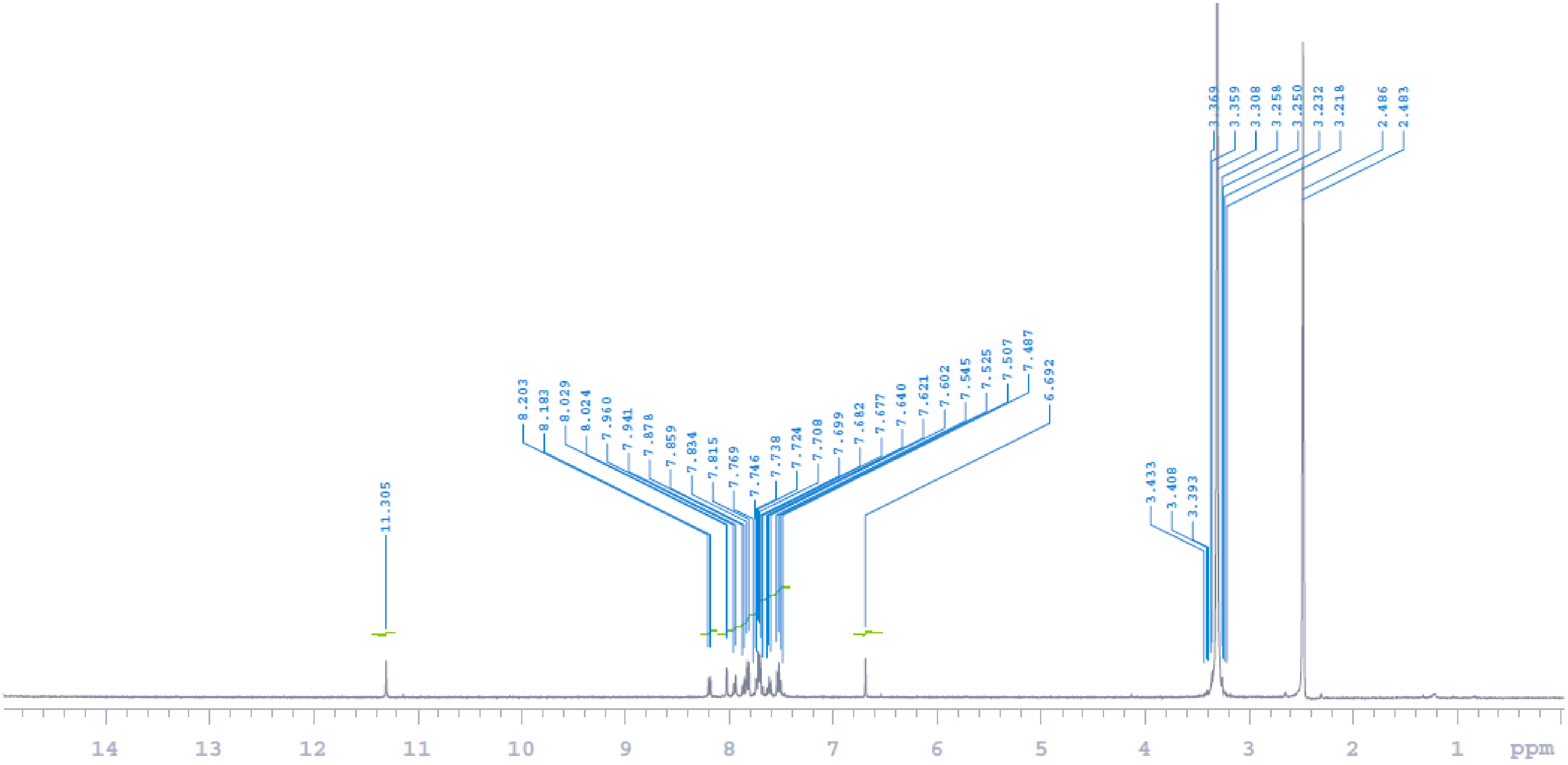

^{1}H-NMR spectrum of compound 8a

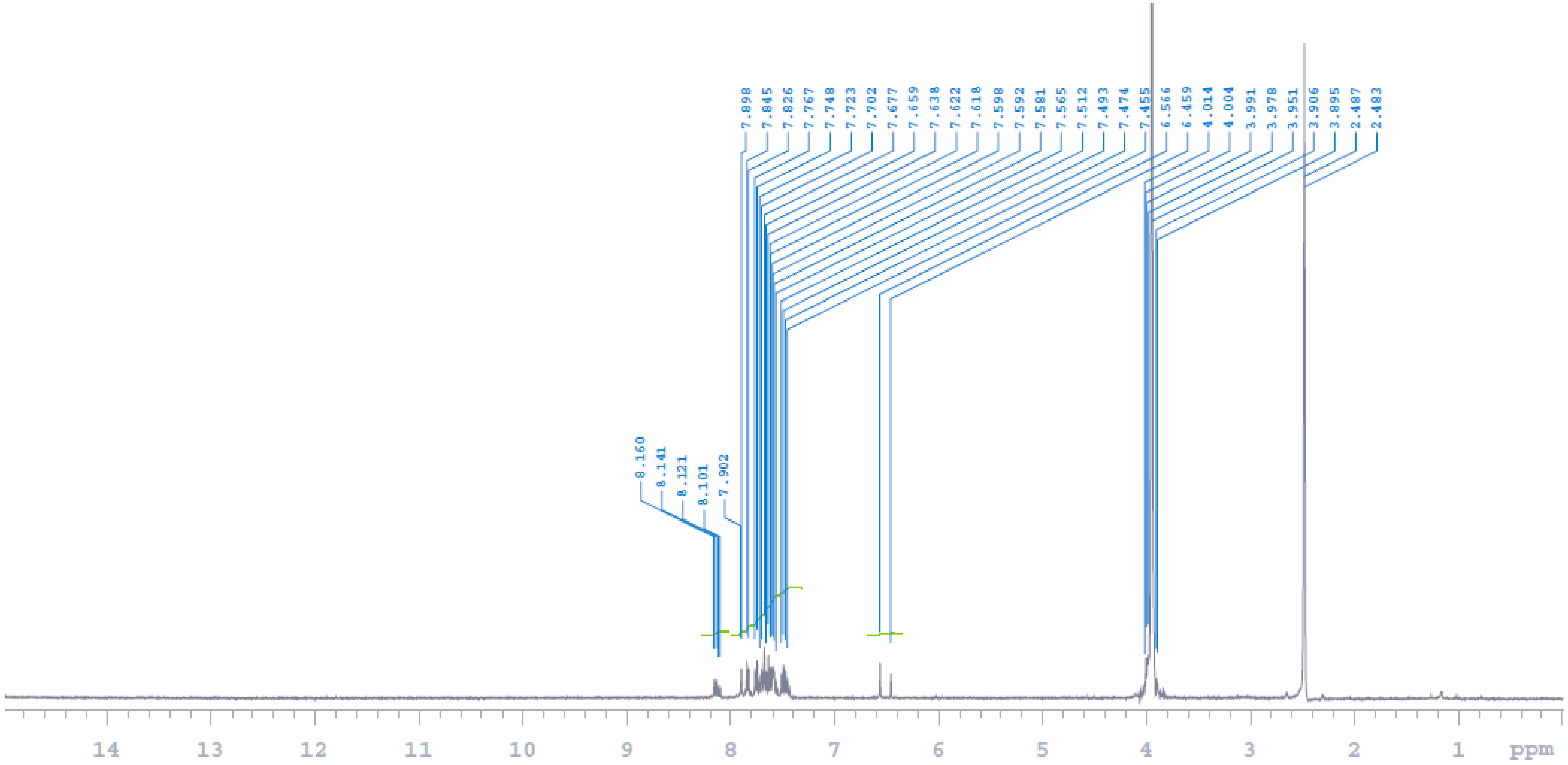

^{1}H-NMR spectrum of compound 8a D_2O

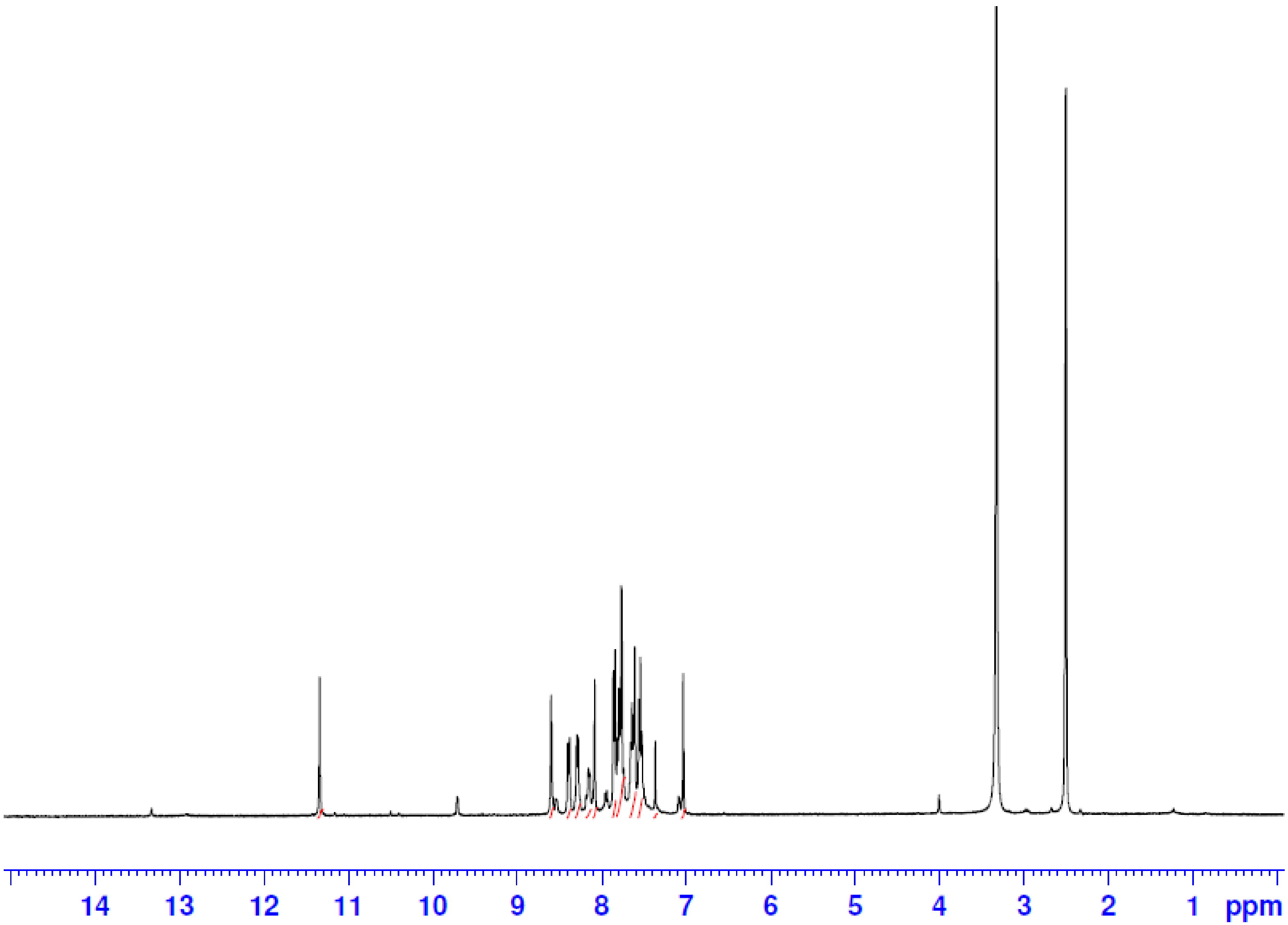

^{1}H-NMR spectrum of compound 8b

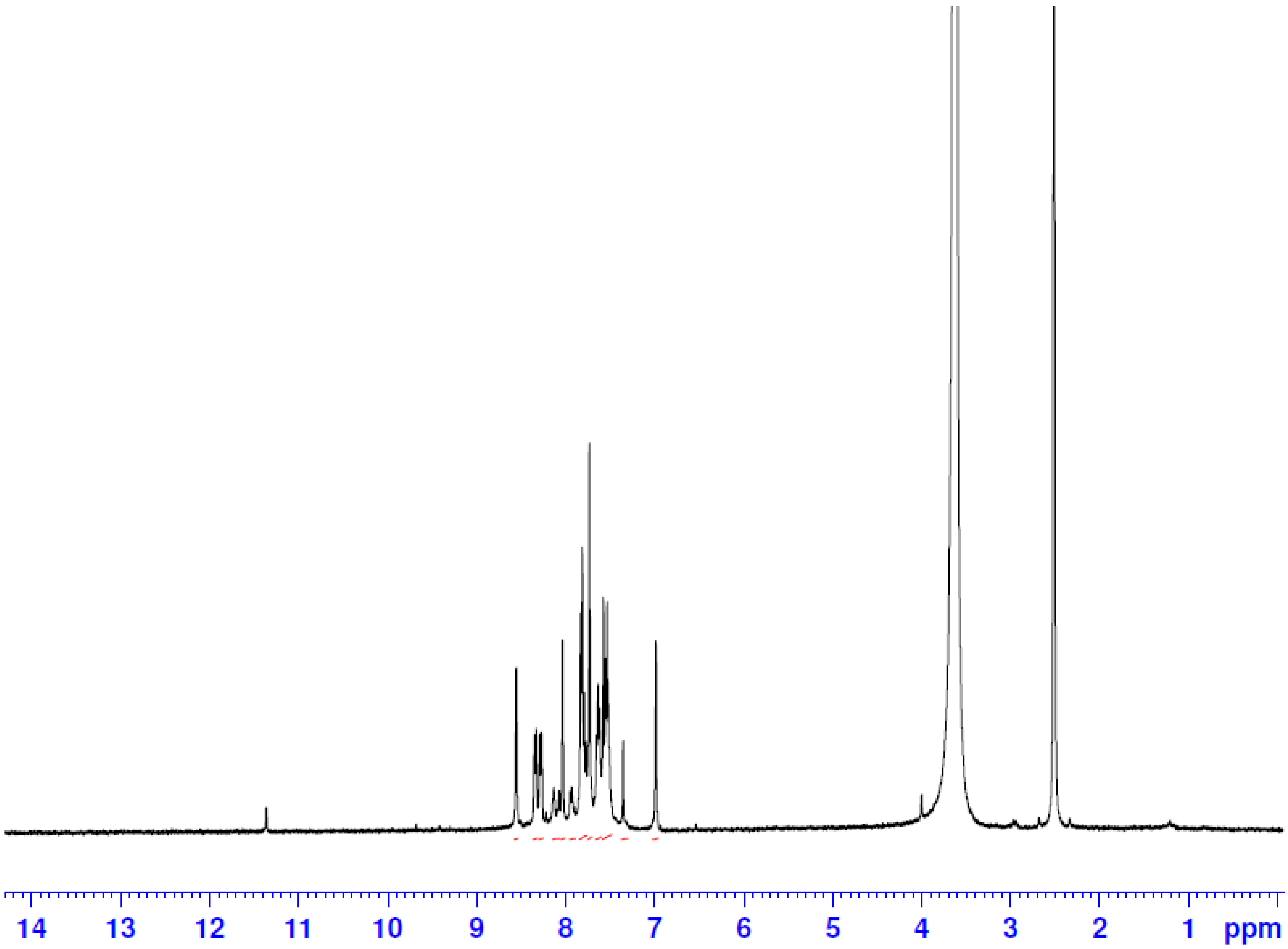

^{1}H-NMR spectrum of compound 8b D_2O

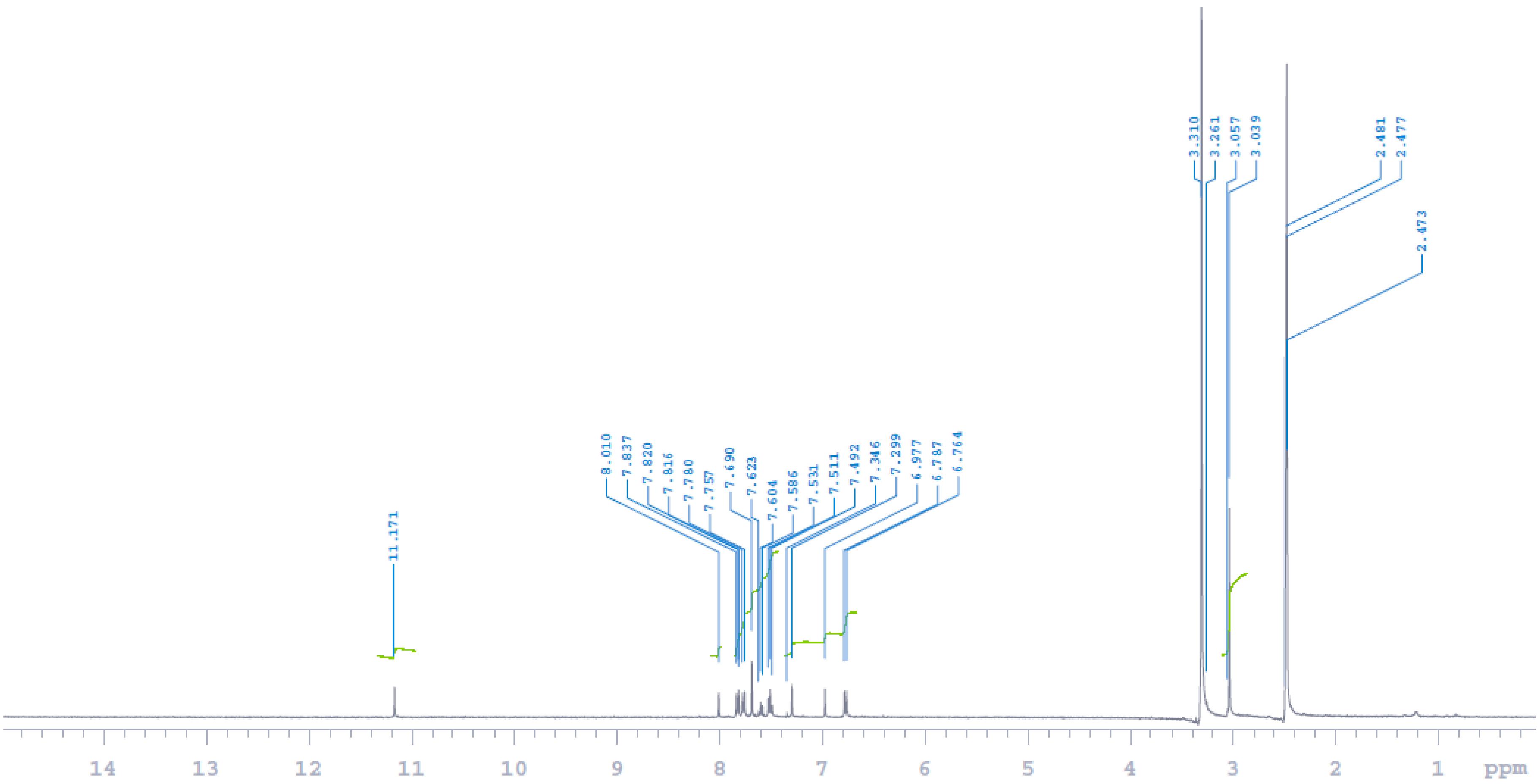

^{1}H-NMR spectrum of compound 8c

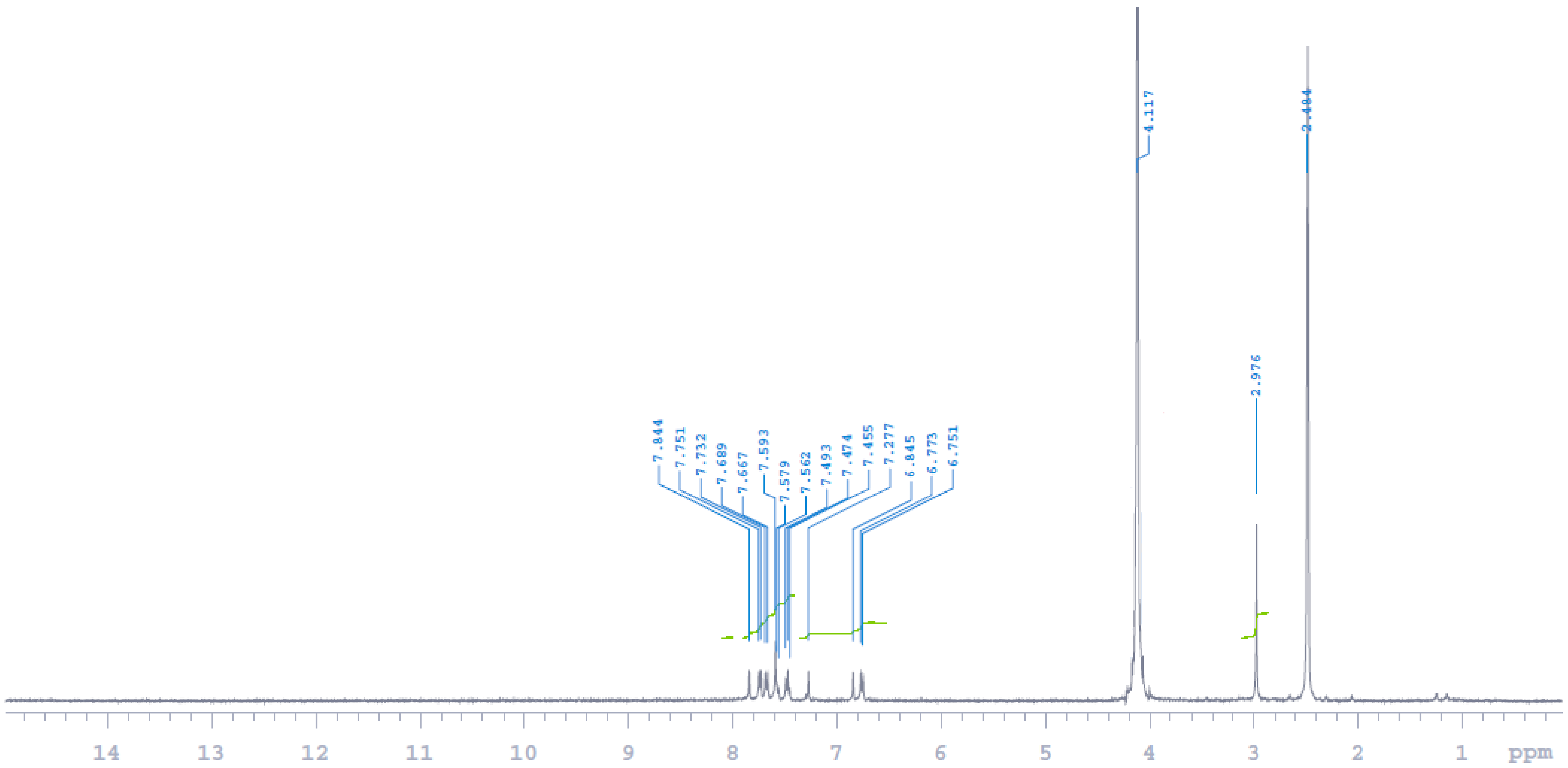

^{1}H-NMR spectrum of compound 8c D_2O

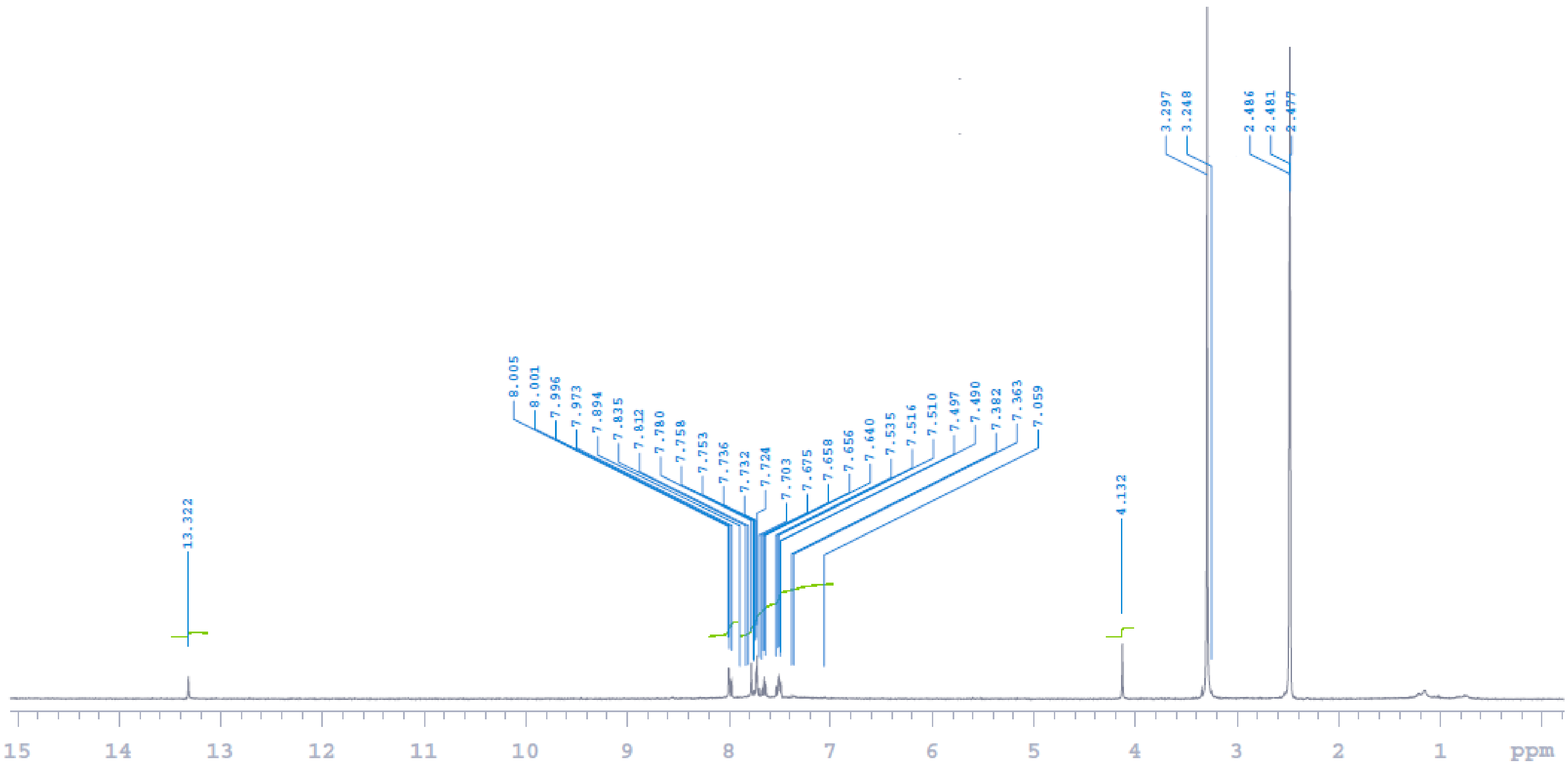

^{1}H-NMR spectrum of compound 9a

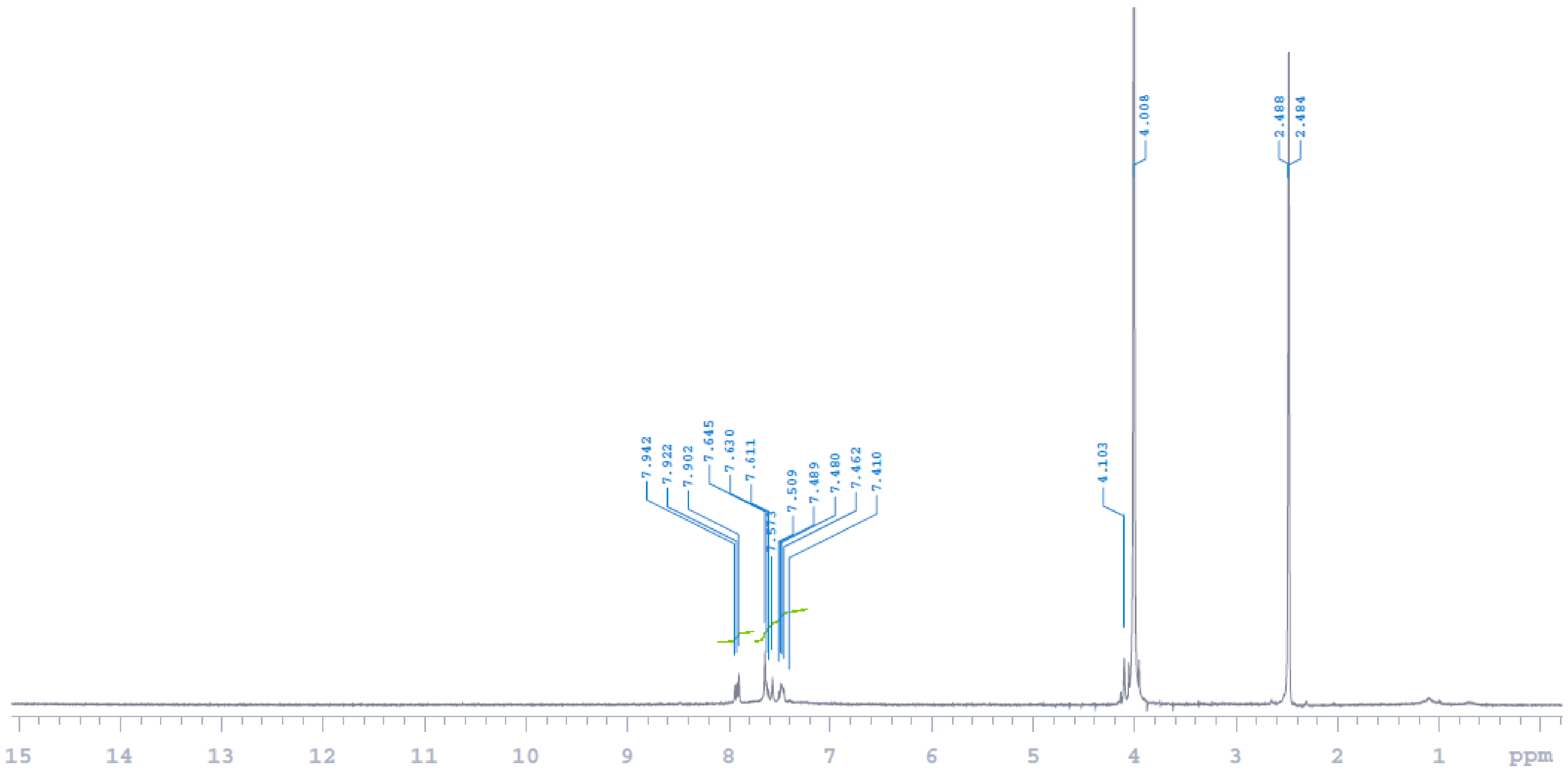

^{1}H-NMR spectrum of compound 9a D_2O

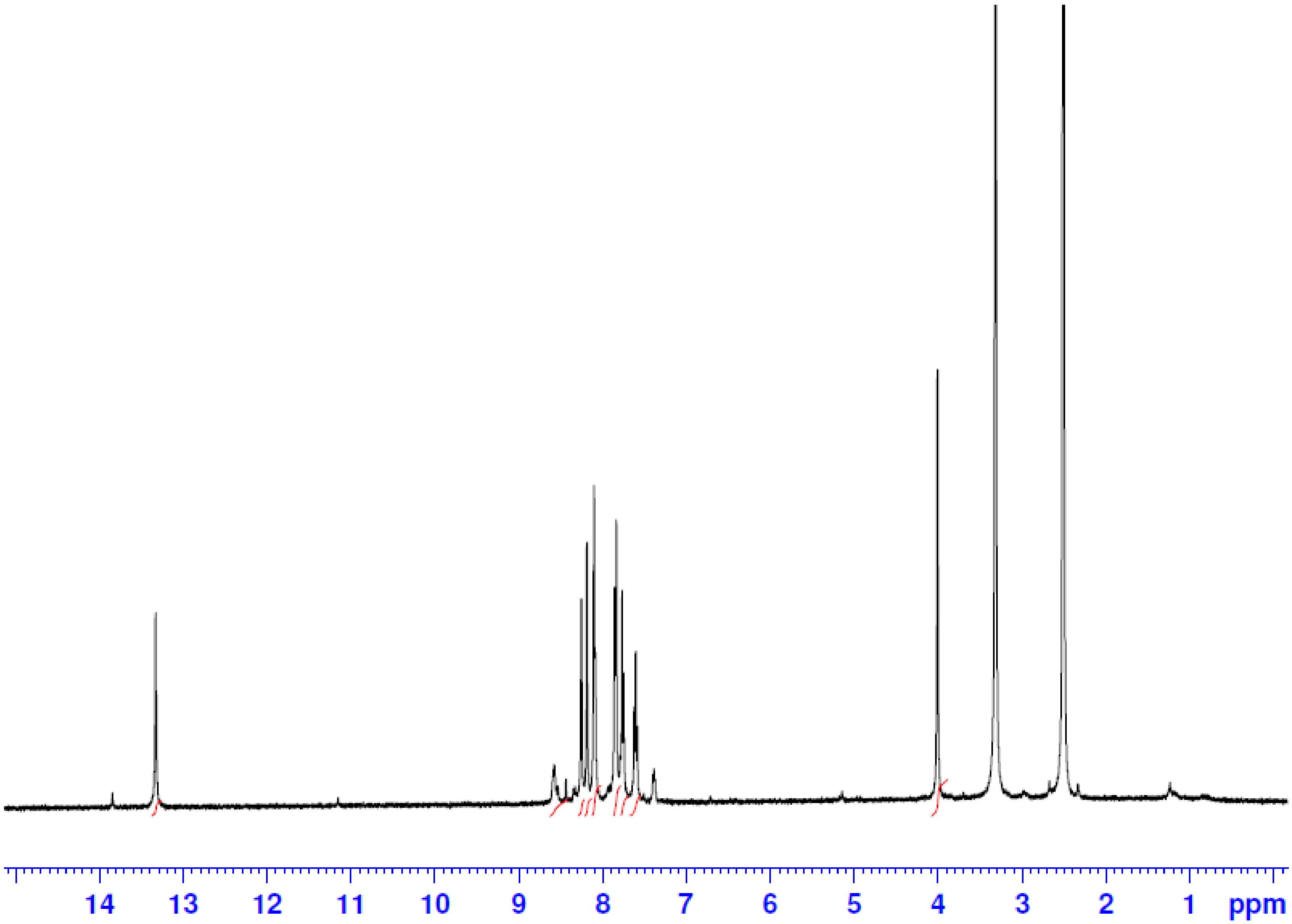

^{1}H-NMR spectrum of compound 9b

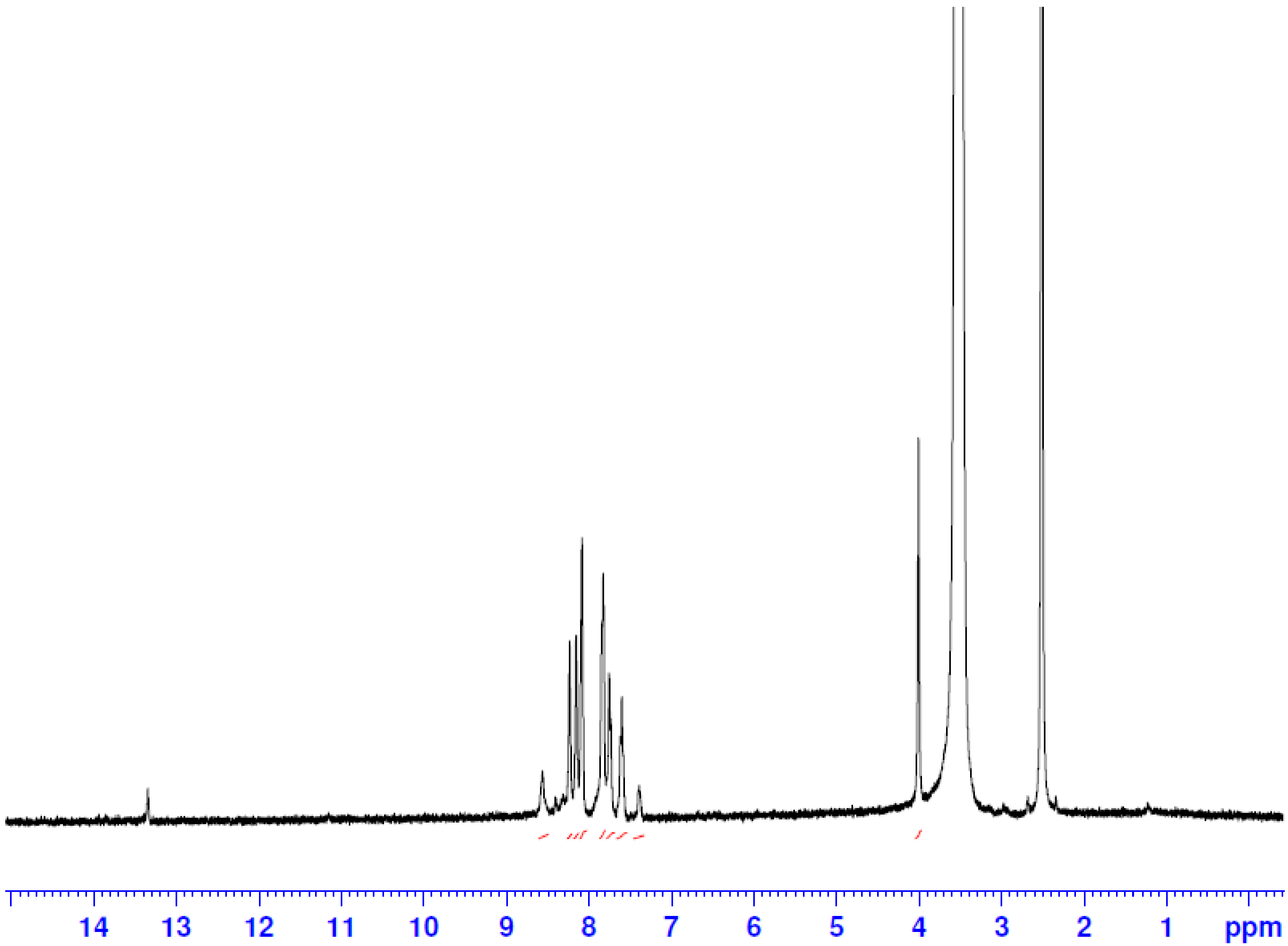

^{1}H-NMR spectrum of compound 9b D_2O

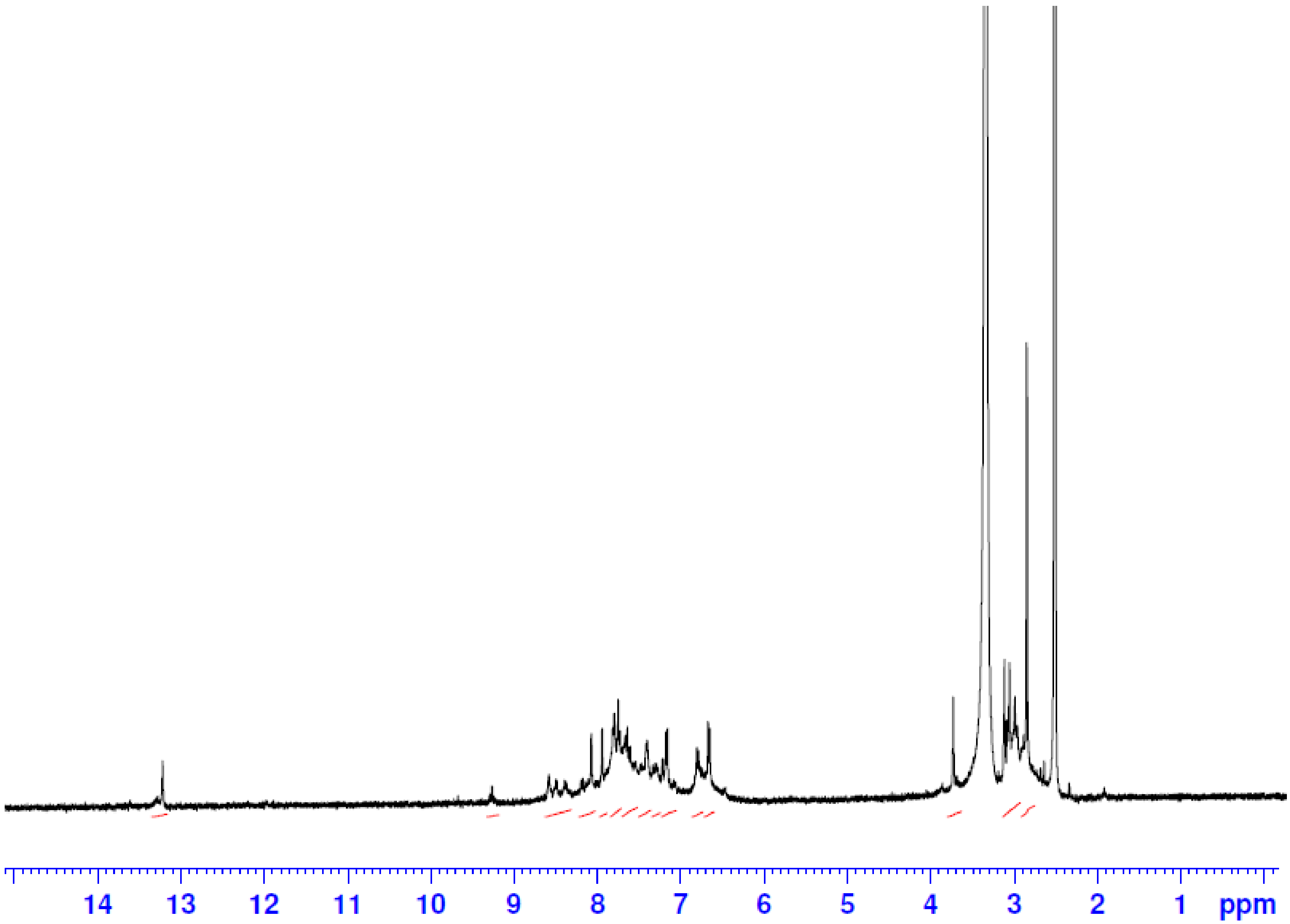

^{1}H-NMR spectrum of compound 9c

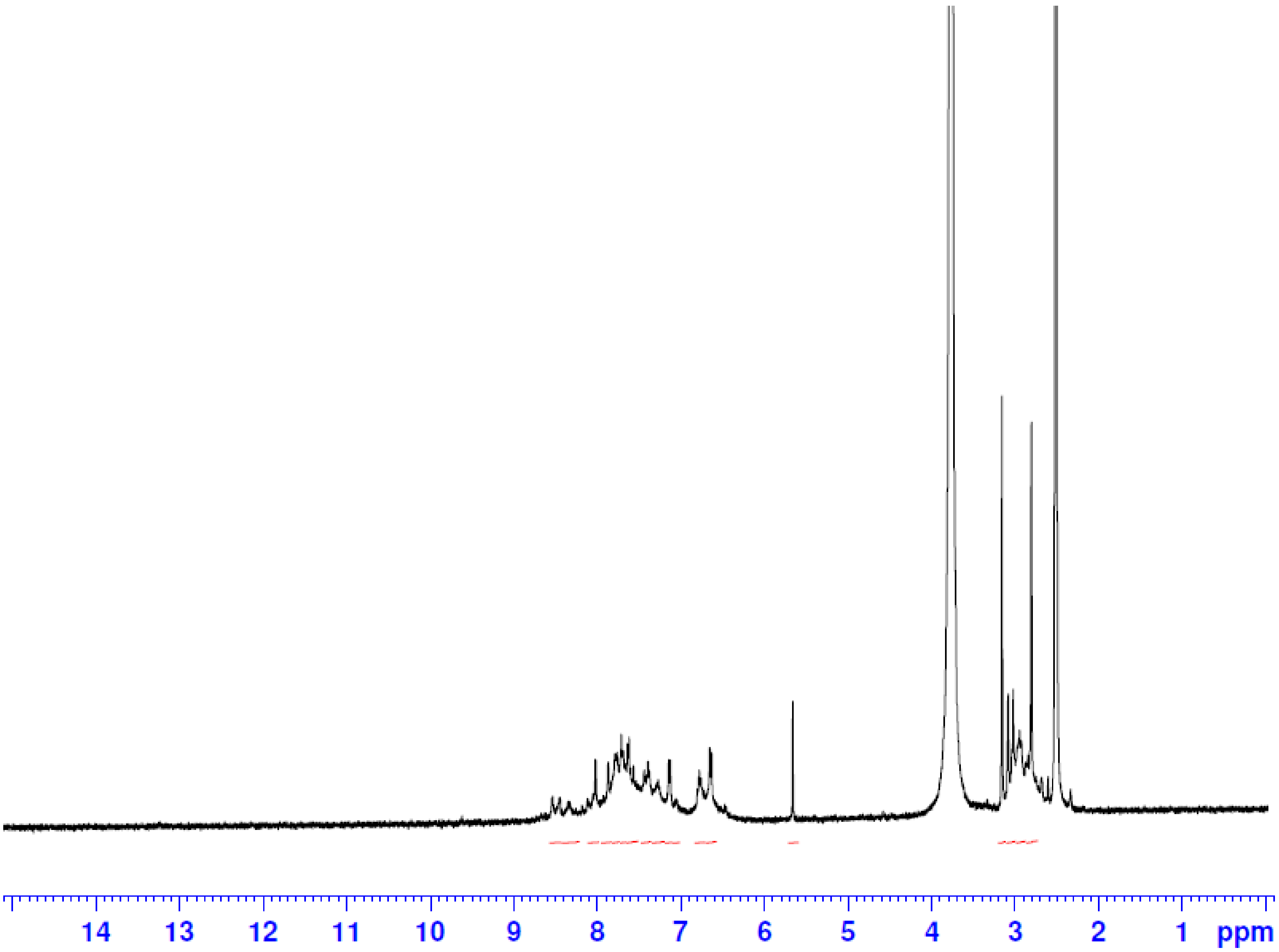

^{1}H-NMR spectrum of compound 9c D_2O
